# Supplementary material for: EBV miRNAs BART11 and BART17-3p promote immune escape through the enhancer-mediated transcription of PD-L1
Source: Nat Commun. 2022 Feb 14;13:866. doi: 10.1038/s41467-022-28479-2 (PMC8844414; doi:10.1038/s41467-022-28479-2)
Supplement: Supplementary file 1 — Supplementary Information [file 41467_2022_28479_MOESM1_ESM.pdf]

Supplementary Information

Supplementary Fig. 1

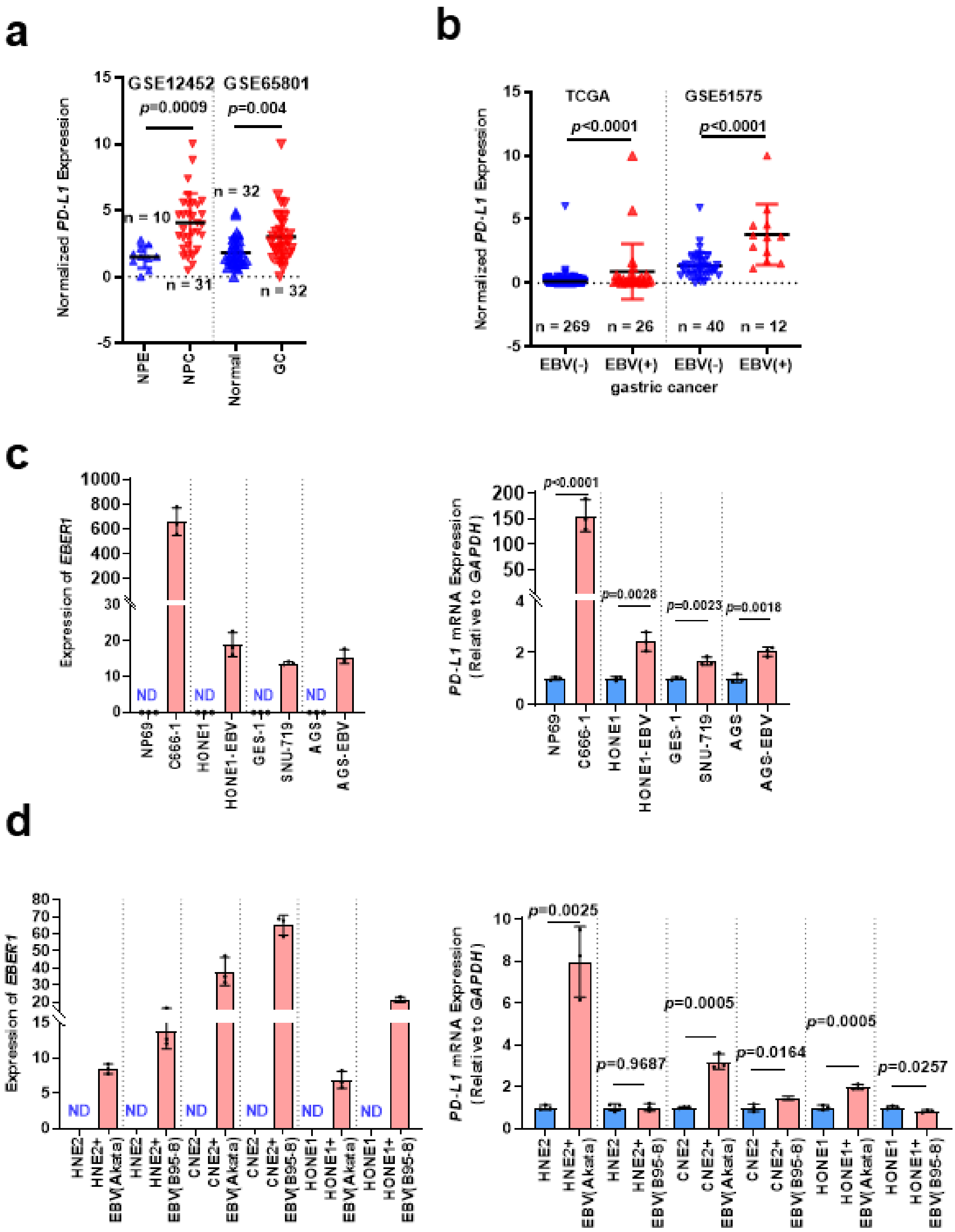

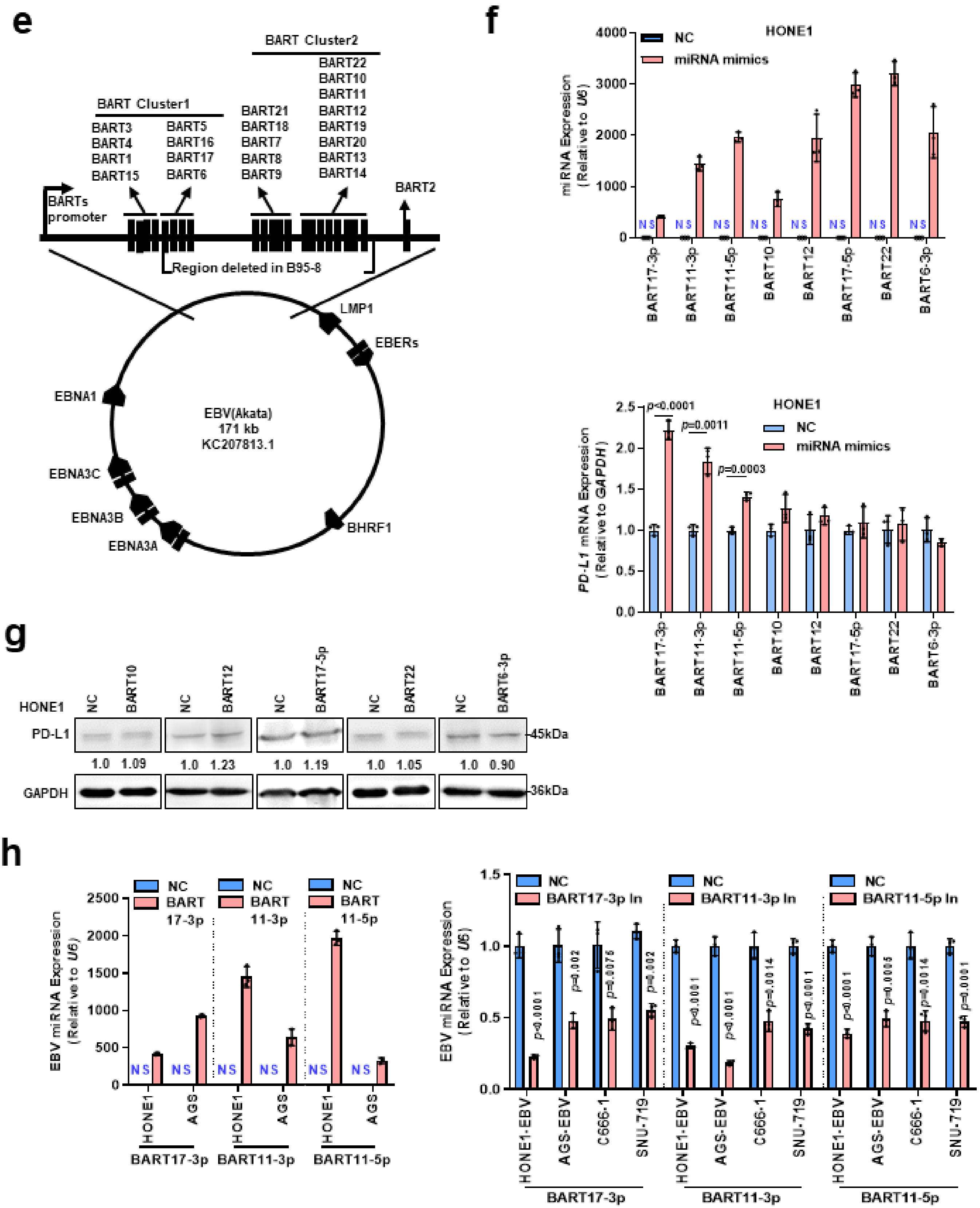
e
BART Cluster2
BART22
BART10
BART11
BART12
BART19
BART20
BART13
BART14
BART Cluster1
BART3
BART4
BART1
BART15
BART5
BART16
BART17
BART6
BART21
BART18
BART7
BART8
BART9
BART2
BARTs promoter
Region deleted in B95-8
LMP1
EBERs
EBNA1
EBV(Akata)
171 kb
KC207813.1
EBNA3C
EBNA3B
EBNA3A
BHRF1
f
HONE1
NC
miRNA mimics
miRNA Expression (Relative to U6)
NS
BART17-3p
BART11-3p
BART11-5p
BART10
BART12
BART17-5p
BART22
BART6-3p
p<0.0001
p=0.0011
p=0.0003
PD-L1 mRNA Expression (Relative to GAPDH)
g
HONE1
NC
BART10
BART12
BART17-5p
BART22
BART6-3p
PD-L1
45kDa
1.0 1.09
1.0 1.23
1.0 1.19
1.0 1.05
1.0 0.90
GAPDH
36kDa
h
NC
BART 17-3p
BART 11-3p
BART 11-5p
EBV miRNA Expression (Relative to U6)
HONE1
AGS
BART17-3p
BART11-3p
BART11-5p
BART17-3p In
BART11-3p In
BART11-5p In
HONE1-EBV
AGS-EBV
C666-1
SNU-719

Supplementary Fig. 1 EBV-miR-BART11 and EBV-miR-BART17-3p upregulate PD-L1 expression in NPC and GC.

a *PD-L1* expression was analyzed in 31 NPCs and 10 NPEs of GSE12452 and 32 GC tissues and 32 normal gastric mucosa tissues of GSE65801. NPC, nasopharyngeal carcinoma; NPE, nasopharyngeal epithelial; GC, gastric adenocarcinoma.

b *PD-L1* expression was positively associated with EBV infection in GC samples based on data from the TCGA database and GSE51575.

c *EBER1* and *PD-L1* expression was analyzed via qRT-PCR in NP69 and C666-1, HONE1 and HONE1-EBV, GES-1, SNU-719, AGS, and AGS-EBV cells. *GAPDH* was used as an internal control.

d *EBER1* and *PD-L1* expression was analyzed via qRT-PCR in HNE2, CNE2 and HONE1 cells which were infected with EBV virions derived from Akata or B95-8 cell lines. *GAPDH* was used as an internal control.

e Schematic diagram of the BART clusters in EBV derived from Akata and the differences with EBV derived from B95-8 cells.

f qRT-PCR analysis of *PD-L1* mRNA expression in HONE1 cells after transfection with different EBV miRNA (EBV-miR-BART17-3p, EBV-miR-BART11-3p, EBV-miR-BART11-5p, EBV-miR-BART10, EBV-miR-BART12, EBV-miR-BART17-5p, EBV-miR-BART22, or EBV-miR-BART6-3p) mimics. *GAPDH* and *U6* were used as an internal control. n = 3 biologically independent samples.

g Western blotting to quantify the protein levels of PD-L1 in HONE1 cells transfected with EBV-miR-BART10, EBV-miR-BART12, EBV-miR-BART17-5p, EBV-miR-BART22, or EBV-miR-BART6-3p mimics. GAPDH was used as an internal control.

h qRT-PCR analysis of EBV-miR-BART17-3p, EBV-miR-BART11-3p, and EBV-miR-BART11-5p expression in EBV-negative HONE1 and AGS cells transfected with EBV-miR-BART17-3p, EBV-miR-BART11-3p or EBV-miR-BART11-5p mimics, and EBV-positive HONE1-EBV, AGS-EBV, C666-1, and SNU-719 cells transfected with EBV-miR-BART17-3p, EBV-miR-BART11-3p, or EBV-miR-BART11-5p inhibitors. *U6* was used as an internal control. n = 3 biologically independent samples.

Data are presented as mean ± s.d, *p* values are calculated by unpaired two-sided *t*-test in a-d, f, h. Source data are provided as a Source Data file.

Supplementary Fig. 2

a

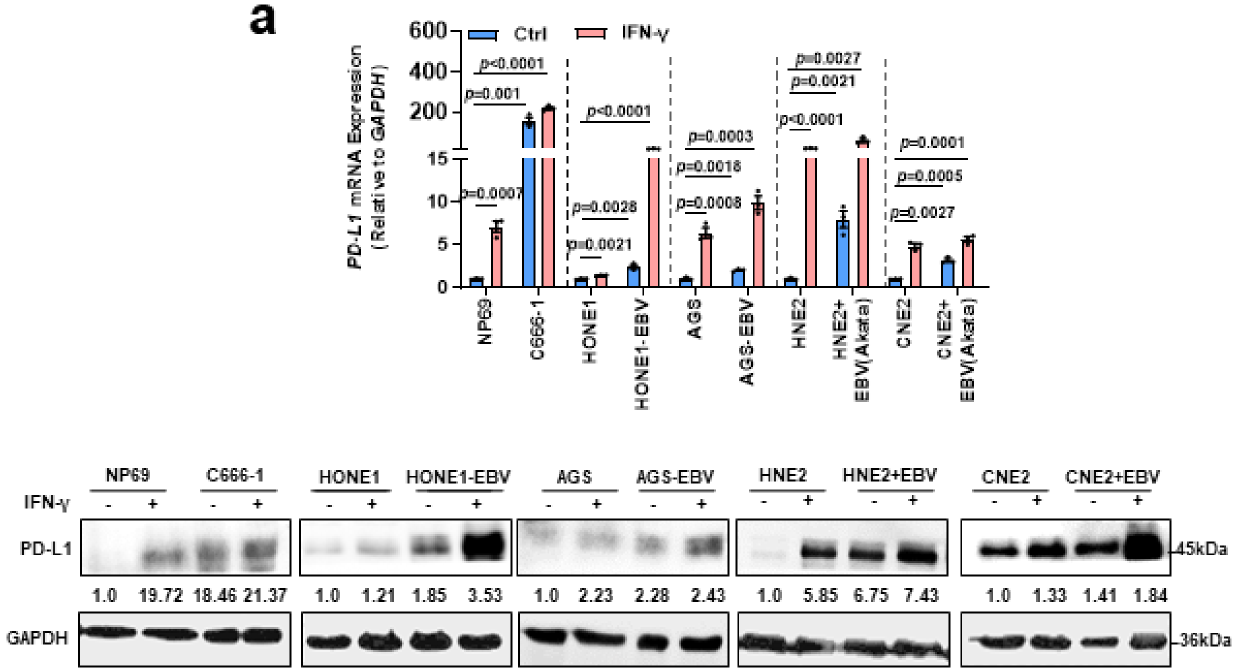

b

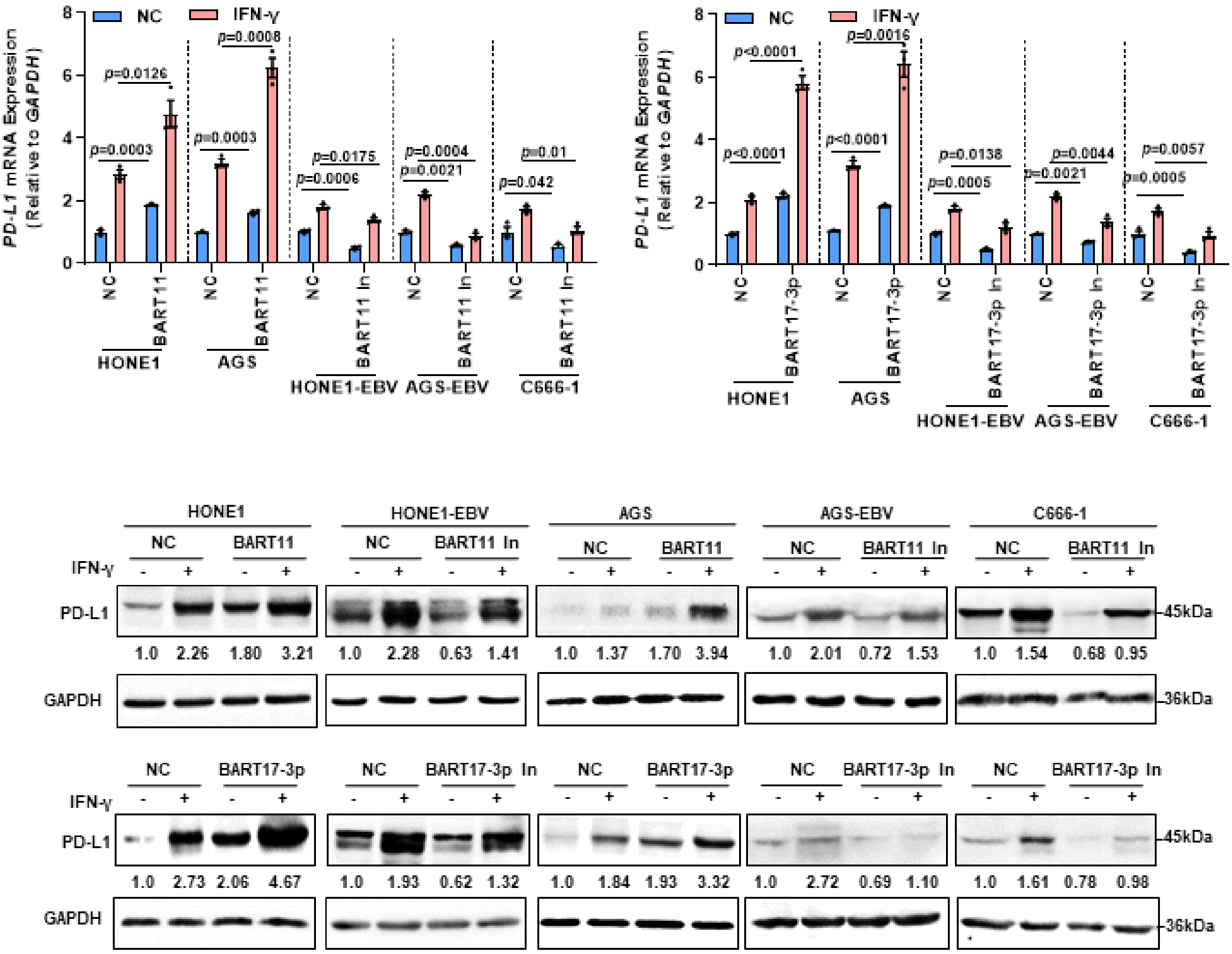

Supplementary Fig. 2 EBV further enhanced the effect of IFN-γ in upregulating PD-L1 expression.

a qRT-PCR and western blotting were performed to quantify PD-L1 expression after IFN-γ stimulation, respectively, in EBV-negative immortalized NPE cell line NP69 and EBV-positive NPC cell line C666-1, NPC cells HONE1 and HONE1-EBV stably transfected with EBV (Akata-derived), GC cells AGS and AGS-EBV stably transfected with EBV (Akata-derived), NPC cells HNE2, CNE2 and HNE2, CNE2 infected with EBV derived from Akata or B95-8. *GAPDH* was used as an internal control. n = 3 biologically independent samples.

b qRT-PCR and western blotting were used to detect PD-L1 expression after IFN-γ stimulation in HONE1 and AGS cells, respectively, transfected with EBV-miR-BART11 or EBV-miR-BART17-3p mimics, and EBV-positive HONE1-EBV, AGS-EBV, C666-1, and SNU-719 transfected with EBV-miR-BART11 or EBV-miR-BART17-3p inhibitors. *GAPDH* was used as an internal control. n = 3 biologically independent samples.

Data are presented as mean ± s.d, *p* values are calculated by unpaired two-sided *t*-test in a, b. Source data are provided as a Source Data file.

Supplementary Fig. 3

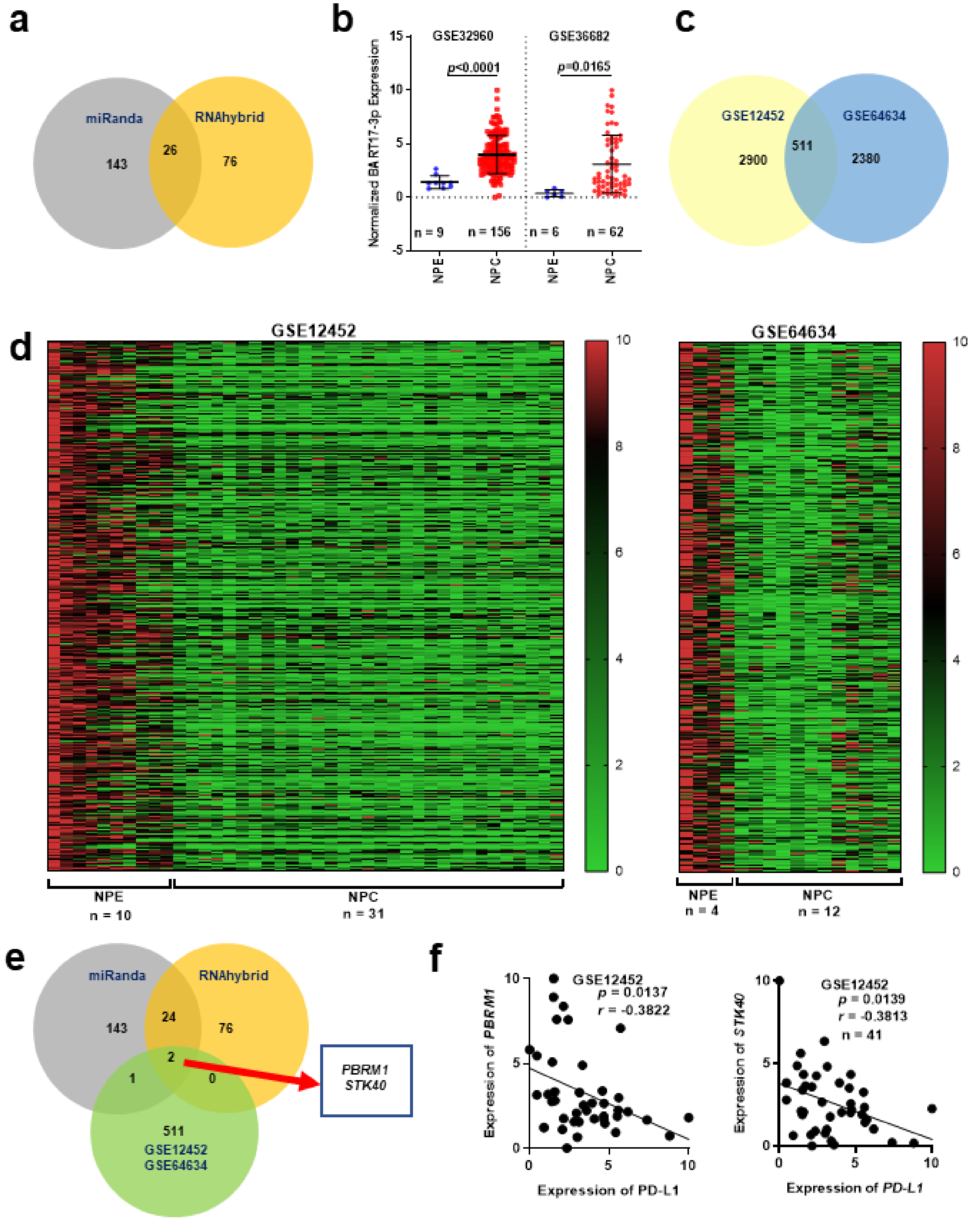

Supplementary Fig. 3 Bioinformatics strategies for screening the downstream genes targeted by EBV-miR-BART17-3p.

a A total of 26 potential targets of EBV-miR-BART17-3p were predicted by miRNA databases such as miRanda and RNAhybrid.

b EBV-miR-BART17-3p expression was high in 156 NPCs compared to 9 NPEs of GSE32960 and in 62 NPCs compared to 6 NPEs of GSE36682.

c Screening of 511 significantly downregulated genes in NPCs in GSE12452 and GSE64634.

d The heatmap of 511 genes that were simultaneously downregulated in GSE12452 and GSE64634.

e *PBRM1* and *STK40* were predicted as EBV-miR-BART17-3p's targets by miRanda and RNAhybrid and were also included in the 511 significantly downregulated genes in NPCs analyzed using the GSE12452 and GSE64634 datasets.

f The correlation of *PBRM1 or STK40* with *PD-L1* expression was analyzed based on the data from GSE12452.

Data are presented as mean ± s.d, *p* values are calculated by unpaired two-sided *t*-test in b. f are calculated by *linear regression*. Source data are provided as a Source Data file.

Supplementary Fig. 4

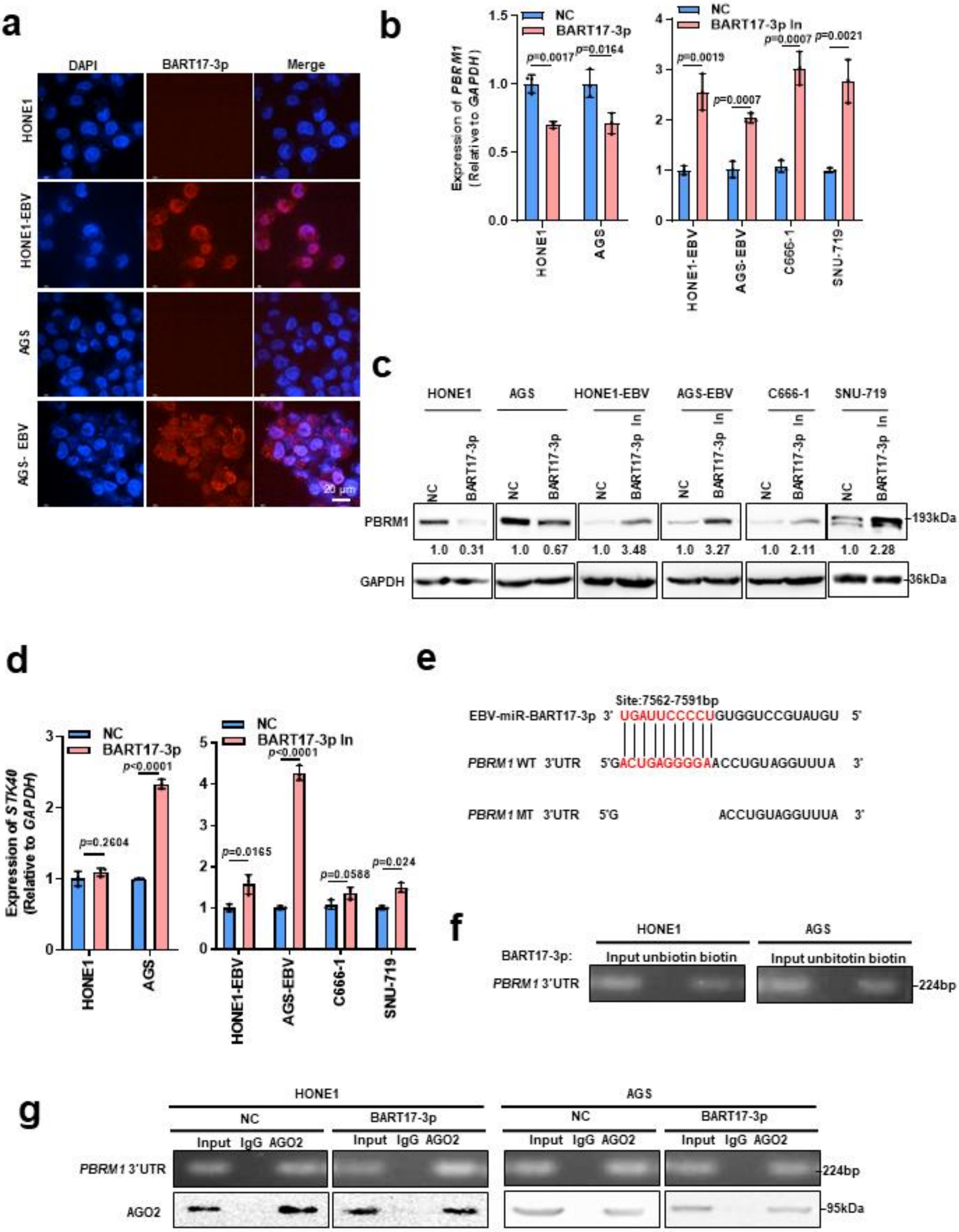

Supplementary Fig. 4 EBV-miBART17-3p directly targets *PBRM1*.

a Using the digoxigenin-labeled probes for EBV-miR-BART17-3p, RNA FISH was performed to detect the expression of EBV-miR-BART17-3p in HONE1, HONE1-EBV, AGS, and AGS-EBV cells. DAPI-stained nucleus: blue, EBV-miR-BART17-3p: red, merge: DAPI and EBV-miR-BART17-3p signal superimposed image, magnification: 600×, scale = 20 μm.

b qRT-PCR analysis of the *PBRM1* mRNA expression in EBV-negative HONE1 and AGS cells transfected with EBV-miR-BART17-3p mimics, EBV-positive HONE1-EBV, AGS-EBV, C666-1, and SNU-719 cells transfected with EBV-miR-BART17-3p inhibitors. *GAPDH* was used as an internal control. n = 3 biologically independent samples.

c Western blotting was performed to quantify the PBRM1 expression in EBV-negative HONE1 and AGS cells transfected with EBV-miR-BART17-3p mimics, and EBV-positive HONE1-EBV, AGS-EBV, C666-1, and SNU-719 cells transfected with EBV-miR-BART17-3p inhibitors. GAPDH was used as an internal control.

d qRT-PCR analysis of *STK40* mRNA in EBV-negative HONE1 and AGS cells transfected with EBV-miR-BART17-3p mimics, and EBV-positive HONE1-EBV, AGS-EBV, C666-1, and SNU-719 cells transfected with EBV-miR-BART17-3p inhibitors. *GAPDH* was used as an internal control. n = 3 biologically independent samples.

e The binding site of EBV-miR-BART17-3p in the *PBRM1* 3′UTR region. The wild-type (*PBRM1*-WT) and mutant (*PBRM1*-MT) are indicated.

f RNA pull-down was performed after DNA electrophoresis of qRT-PCR products to verify the binding effect of EBV-miR-BART17-3p on the 3′UTR of *PBRM1* in HONE1 and AGS cells transfected with the biotin-labeled or unlabeled EBV-miR-BART17-3p probes.

g After transfection of EBV-miR-BART17-3p mimics or negative control into EBV-negative HONE1 and AGS cells, the anti-AGO2 antibody was used for RIP experiments and DNA electrophoresis of qRT-PCR products was performed to identify whether EBV-miR-BART17-3p showed binding to the *PBRM1* 3′UTR *via* AGO2.

Data are presented as mean ± s.d, *p* values are calculated by unpaired two-sided *t*-test in b, d. Source data are provided as a Source Data file.

Supplementary Fig. 5

a

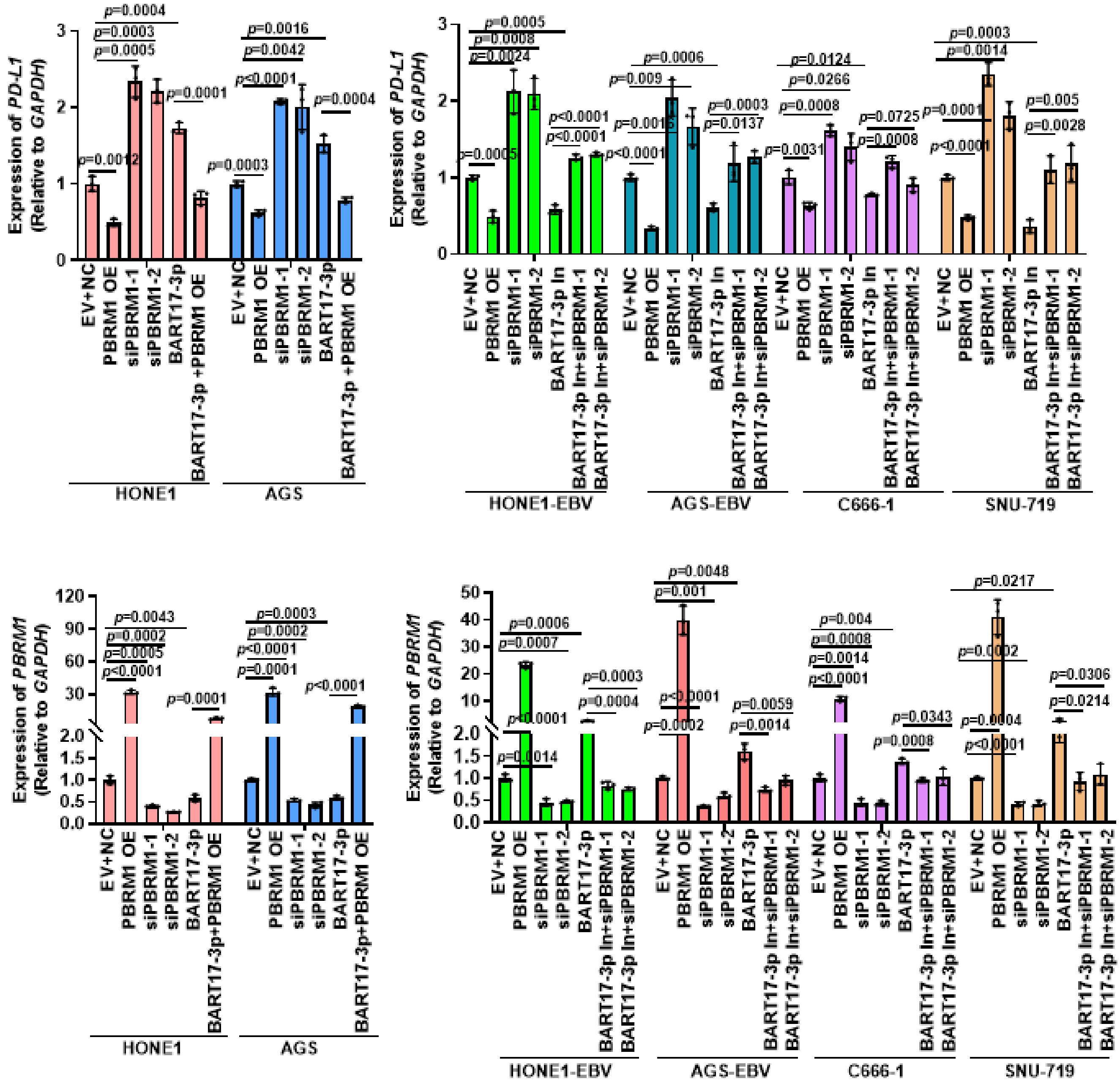

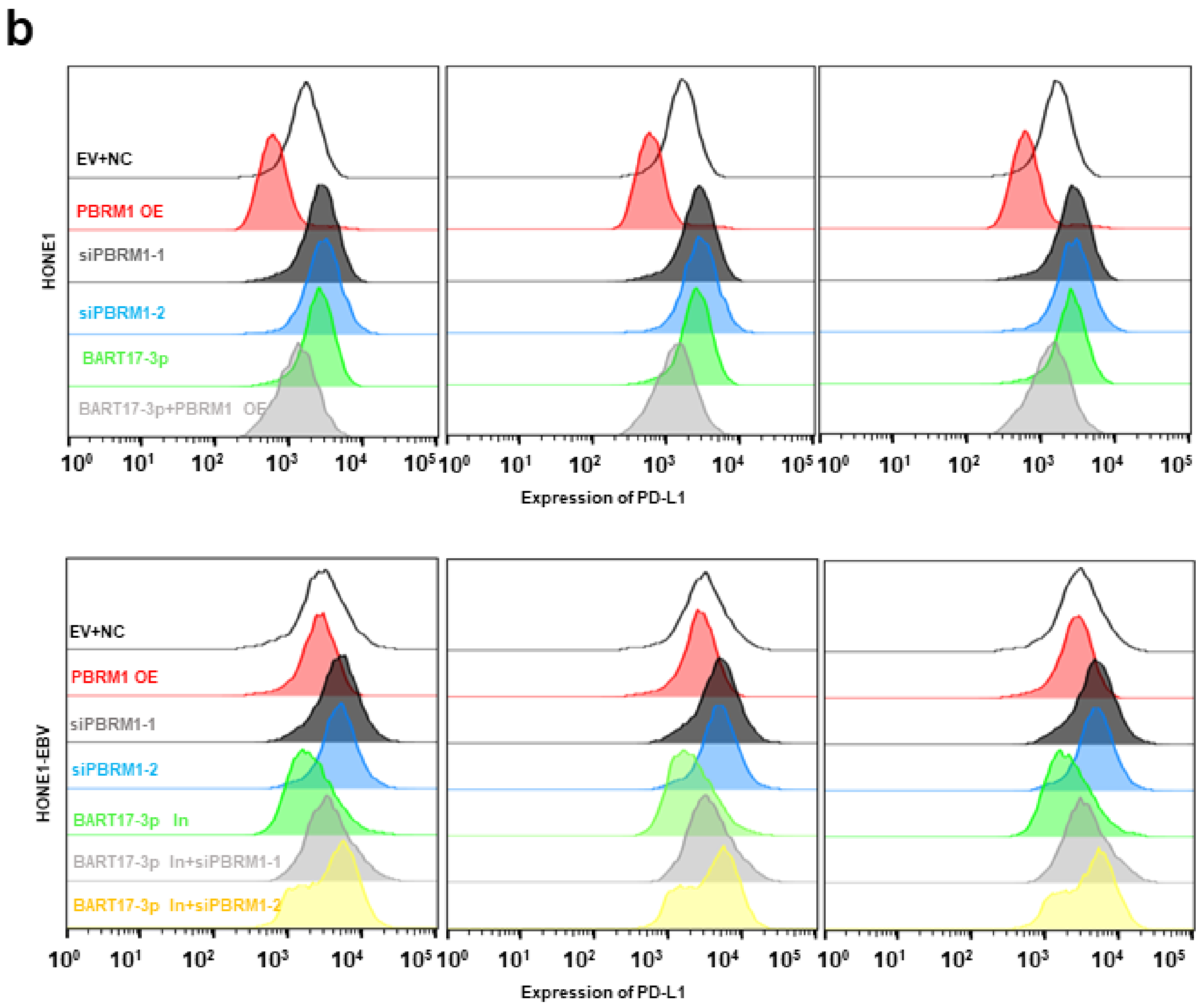

Supplementary Fig. 5 EBV-miR-BART17-3p upregulates PD-L1 expression by targeting *PBRM1*.

a qRT-PCR analysis of EBV-miR-BART17-3p regulation of the *PD-L1* mRNA *via* PBRM1 in HONE1, AGS, HONE1-EBV, AGS-EBV, C666-1, and SNU-719 cells transfected with the PBRM1 overexpression vector, siPBRM1, EBV-miR-BART17-3p mimics or inhibitors, or co-transfected with EBV-miR-BART17-3p mimics and the PBRM1 overexpression vector, or EBV-miR-BART17-3p inhibitors and siPBRM1. *GAPDH* was used as an internal control. n = 3 biologically independent samples.

b Flow cytometric analysis of PD-L1 expression in HONE1 and HONE1-EBV cells transfected with the PBRM1 overexpression vector, siPBRM1, EBV-miR-BART17-3p mimics or inhibitors, or co-transfected with EBV-miR-BART17-3p mimics and the PBRM1 overexpression vector, or EBV-miR-BART17-3p

inhibitors and siPBRM1. n = 3 biologically independent samples, and the statistical results are shown in Fig. 2f.

Data are presented as mean ± s.d, p values are calculated by unpaired two-sided t-test in a. Source data are provided as a Source Data file.

Supplementary Fig. 6

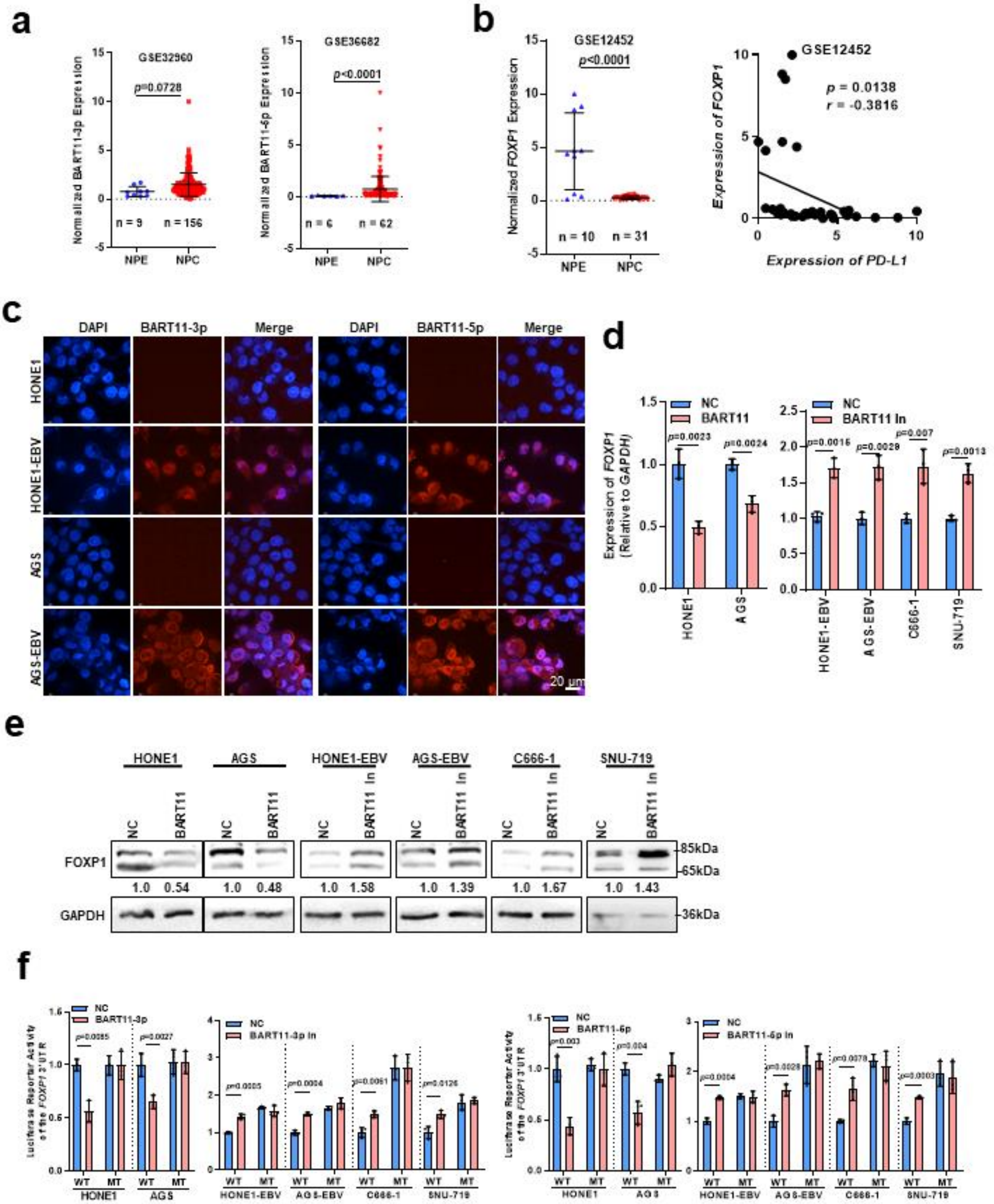

Supplementary Fig. 6 EBV-miBART11 directly targets *FOXP1*.

a EBV-miR-BART11-3p was highly expressed in 156 NPCs compared with the 9 NPE tissue samples from GSE32960, and EBV-miR-BART11-5p expression was high in 62 NPCs compared to 6 NPEs of GSE36682.

b *FOXP1* expression and the correlation between *FOXP1* and *PD-L1* expression was analyzed in 31 NPCs and 10 NPEs from GSE12452.

c Using the digoxigenin-labeled probe for EBV-miR-BART11-3p and EBV-miR-BART11-5p, RNA FISH was performed to detect the expression of EBV-miR-BART11-3p and EBV-miR-BART11-5p in HONE1, HONE1-EBV, AGS, and AGS-EBV cells. DAPI-stained nucleus: blue, EBV-miR-BART11-3p or EBV-miR-BART11-5p: red, merge: DAPI and EBV-miR-BART11-3p or EBV-miR-BART11-5p signal superimposed image, magnification: 600×, scale = 20 μm.

d qRT-PCR analysis of *FOXP1* mRNA in EBV-negative HONE1 and AGS cells transfected with EBV-miR-BART11 mimics, and EBV-positive HONE1-EBV, AGS-EBV, C666-1, and SNU-719 cells transfected with EBV-miR-BART11 inhibitors. *GAPDH* was used as an internal control. n = 3 biologically independent samples.

e Western blotting quantification of the FOXP1 protein in EBV-negative HONE1 and AGS cells transfected with EBV-miR-BART11 mimics, and EBV-positive HONE1-EBV, AGS-EBV, C666-1, and SNU-719 cells transfected with EBV-miR-BART11 inhibitors. GAPDH was used as an internal control.

f The luciferase reporter activity was measured in HONE1, AGS, HONE-EBV, AGS-EBV, C666-1, and SNU-719 cells were co-transfected with the *FOXP1*-WT vector or the *FOXP1*-MT vectors and EBV-miR-BART11-3p, EBV-miR-BART11-5p mimics or inhibitors. n = 3 biologically independent samples.

Data are presented as mean ± s.d, *p* values are calculated by unpaired two-sided *t*-test in a, b(left), d, f. b(right) are calculated by *linear regression*. Source data are provided as a Source Data file.

Supplementary Fig. 7

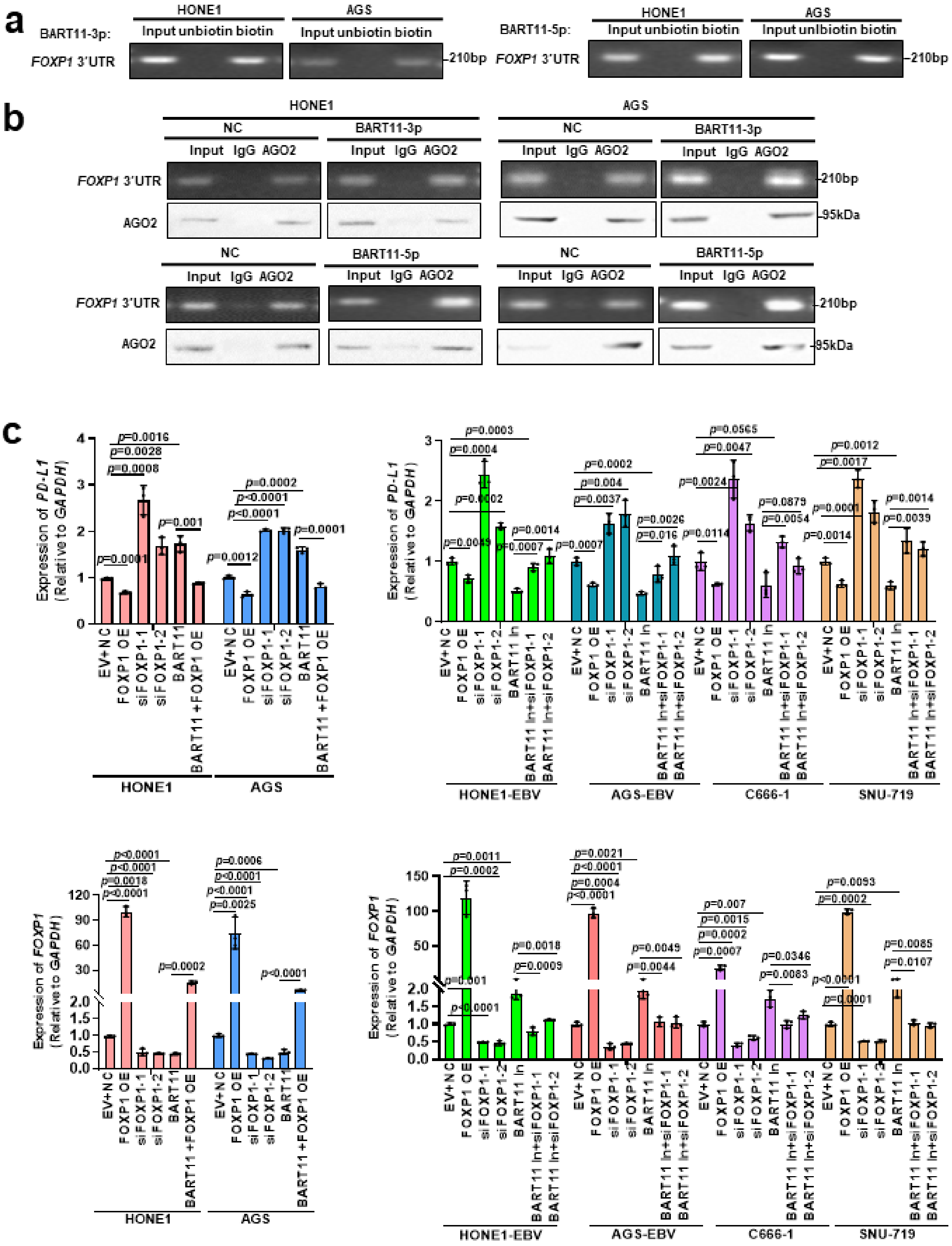

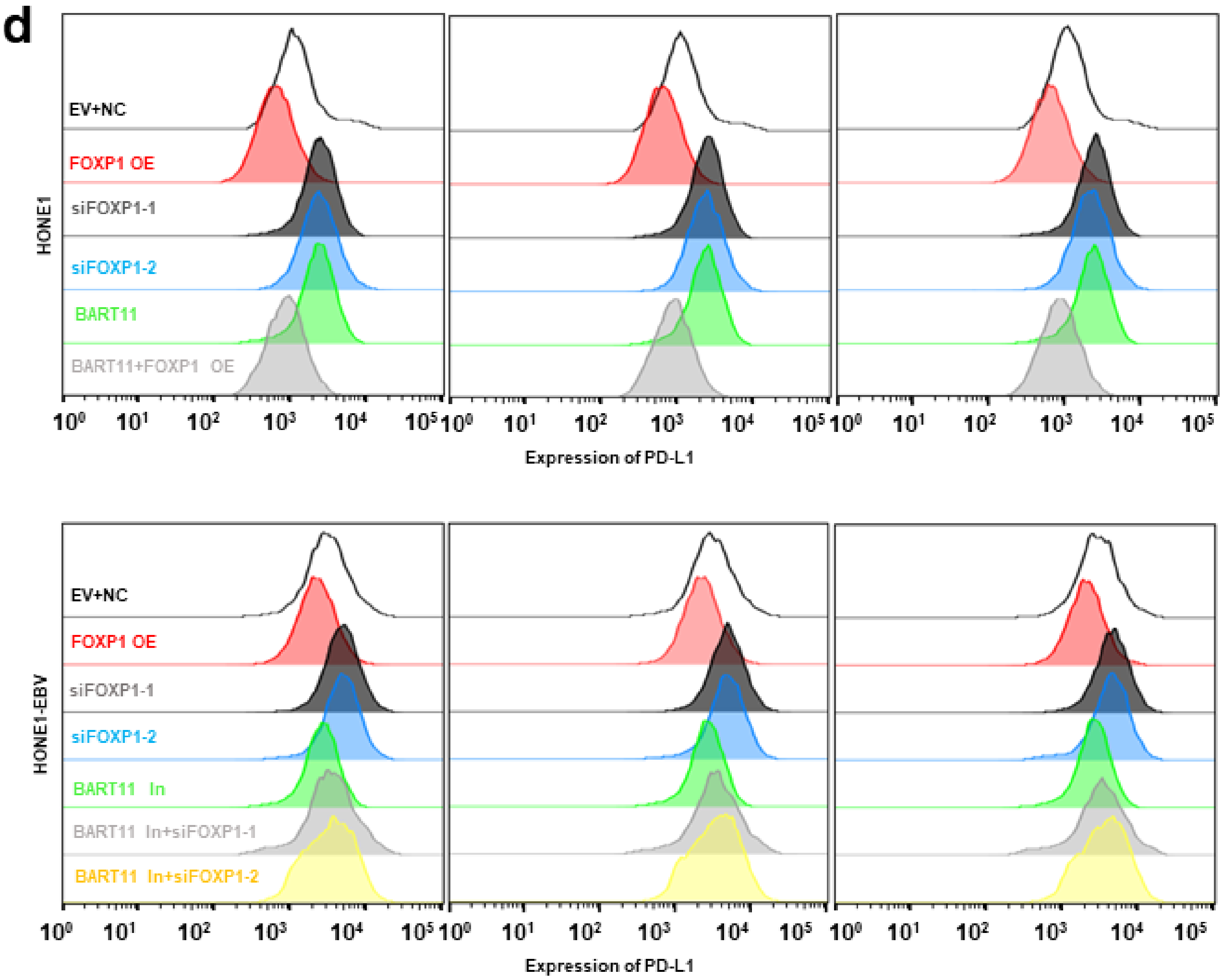

Supplementary Fig. 7 EBV-miR-BART11 upregulates PD-L1 expression by targeting *FOXP1*.

a RNA pull-down and subsequent DNA electrophoresis of qRT-PCR products to identify the binding effect of EBV-miR-BART11-3p and EBV-miR-BART11-5p on the 3′-UTR of *FOXP1* in HONE1 and AGS cells transfected with the biotin-labeled or unlabeled EBV-miR-BART11-3p and EBV-miR-BART11-5p probes.

b After transfecting EBV-miR-BART11-3p, or EBV-miR-BART11-5p mimics or negative control into EBV-negative HONE1 and AGS cells, the anti-AGO2 antibody was used for the RIP experiment and DNA electrophoresis of qRT-PCR products was performed to analyze whether EBV-miR-BART11-3p and EBV-miR-BART11-5p bind the *FOXP1* 3′-UTR *via* AGO2.

c qRT-PCR analysis of EBV-miR-BART11 regulation of *PD-L1* mRNA via FOXP1 in HONE1, AGS, HONE1-EBV, AGS-EBV, C666-1, and SNU-719 cells transfected with the FOXP1 overexpression vector, siFOXP1 EBV-miR-BART11 mimics or inhibitors, or co-transfected with EBV-miR-BART11 mimics

and the FOXP1 overexpression vector, or EBV-miR-BART11 inhibitors and siFOXP1. *GAPDH* was used as an internal control. n = 3 biologically independent samples.

d Flow cytometric analysis of PD-L1 expression in HONE1 and HONE1-EBV cells transfected with the FOXP1 overexpression vector, siFOXP1, EBV-miR-BART11 mimics or inhibitors, or co-transfected with EBV-miR-BART11 mimics and the FOXP1 overexpression vector, or EBV-miR-BART11 inhibitors and siFOXP1. n = 3 biologically independent samples, and the statistical results are shown in Fig. 3e.

Data are presented as mean ± s.d, *p* values are calculated by unpaired two-sided t-test in c. Source data are provided as a Source Data file.

Supplementary Fig. 8

a

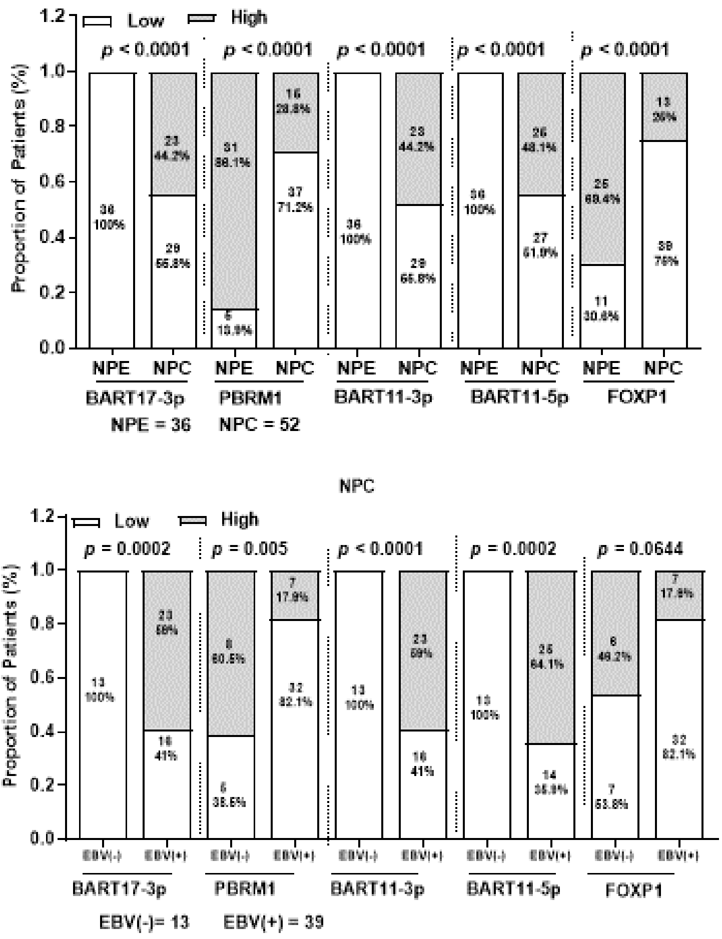

b

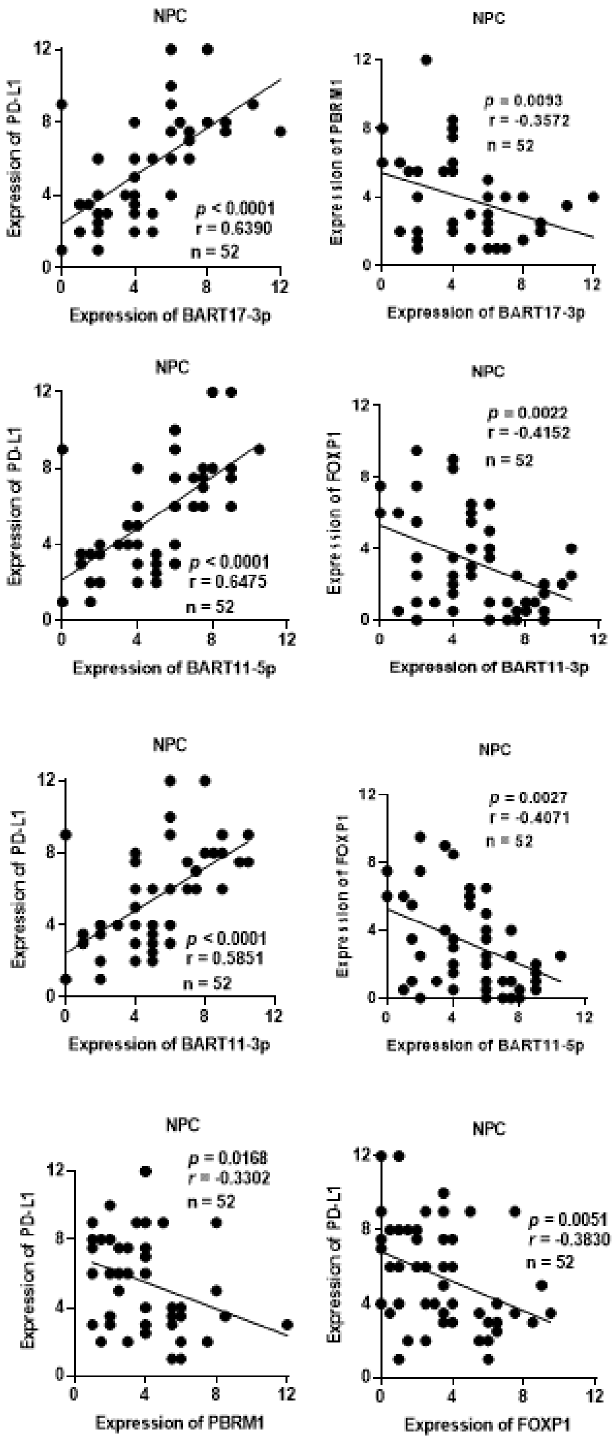

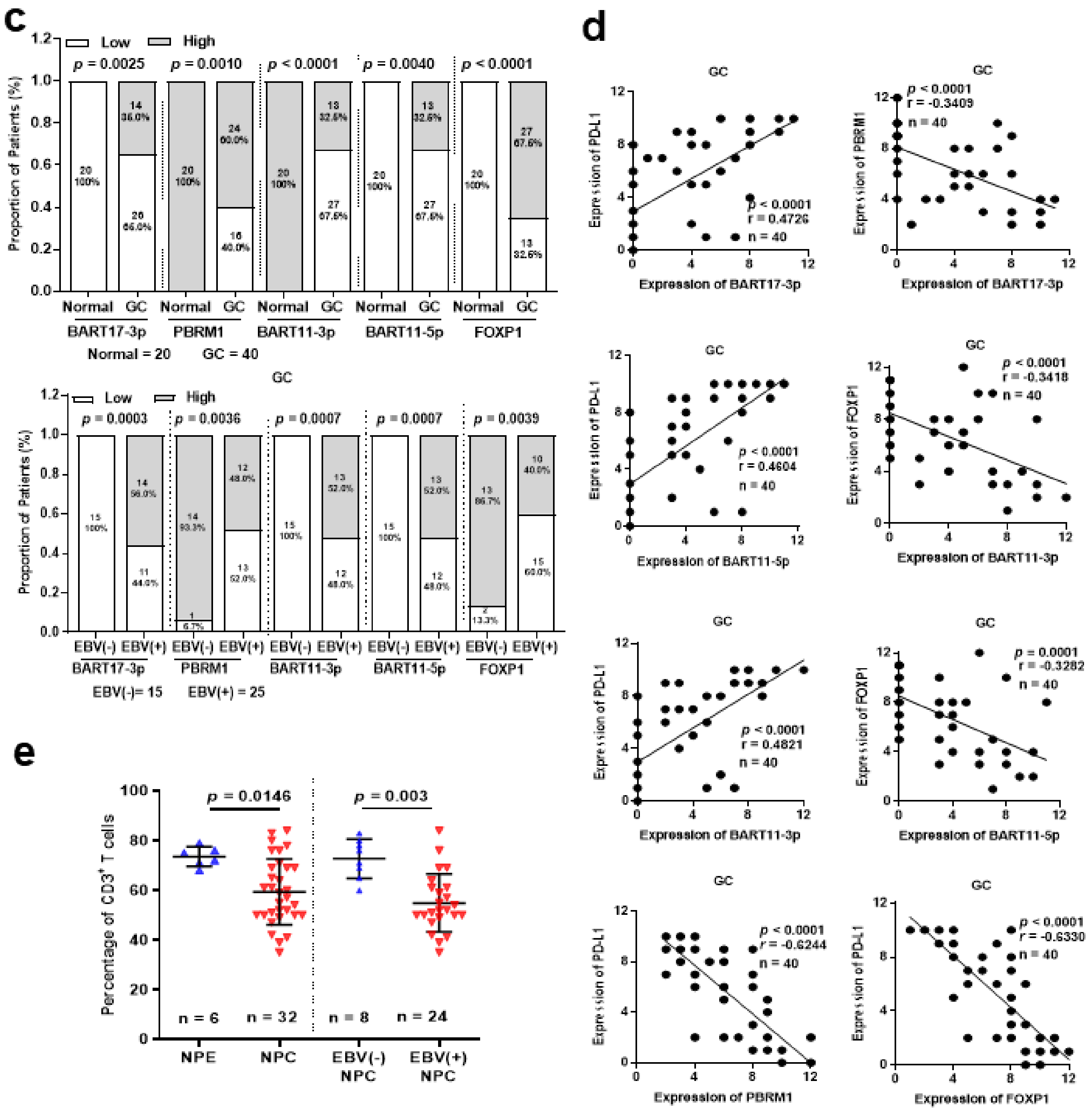

Supplementary Fig. 8 Expression and correlation analysis of EBV-miR-BART17-3p, PBRM1, EBV-miR-BART11-3p, EBV-miR-BART11-5p, and FOXP1 in NPC samples.

a The statistical analysis of EBV-miR-BART17-3p, EBV-miR-BART11-3p, EBV-miR-BART11-5p, FOXP1, and PBRM1 expression in 52 NPCs (13 EBV-negative and 39 EBV-positive) and 36 NPE samples and the correlation with EBV.

b The correlation analysis between EBV-miR-BART17-3p, EBV-miR-BART11-3p, EBV-miR-BART11-5p, PBRM1, FOXP1, and PD-L1 was performed based on the ISH or IHC data in NPC.

c The statistical analysis of EBV-miR-BART17-3p, EBV-miR-BART11-3p, EBV-miR-BART11-5p, FOXP1, and PBRM1 expression in 40 GCs (15 EBV-negative and 25 EBV-positive) and 20 normal gastric samples and the correlation with EBV.

d The correlation analysis between EBV-miR-BART17-3p, EBV-miR-BART11-3p, EBV-miR-BART11-5p, PBRM1, FOXP1, and PD-L1 was performed based on the ISH or IHC data in gastric adenocarcinoma.

e Flow cytometric analysis to detect the percentage of $CD3^+$ T cells in whole blood cells in 32 NPCs (8 EBV-negative and 24 EBV-positive) and samples from 36 normal people.

a, c are calculated by *F*- test. b, d are calculated by *linear regression*. e are calculated by unpaired two-sided *t*-test. Source data are provided as a Source Data file.

Supplementary Fig. 9

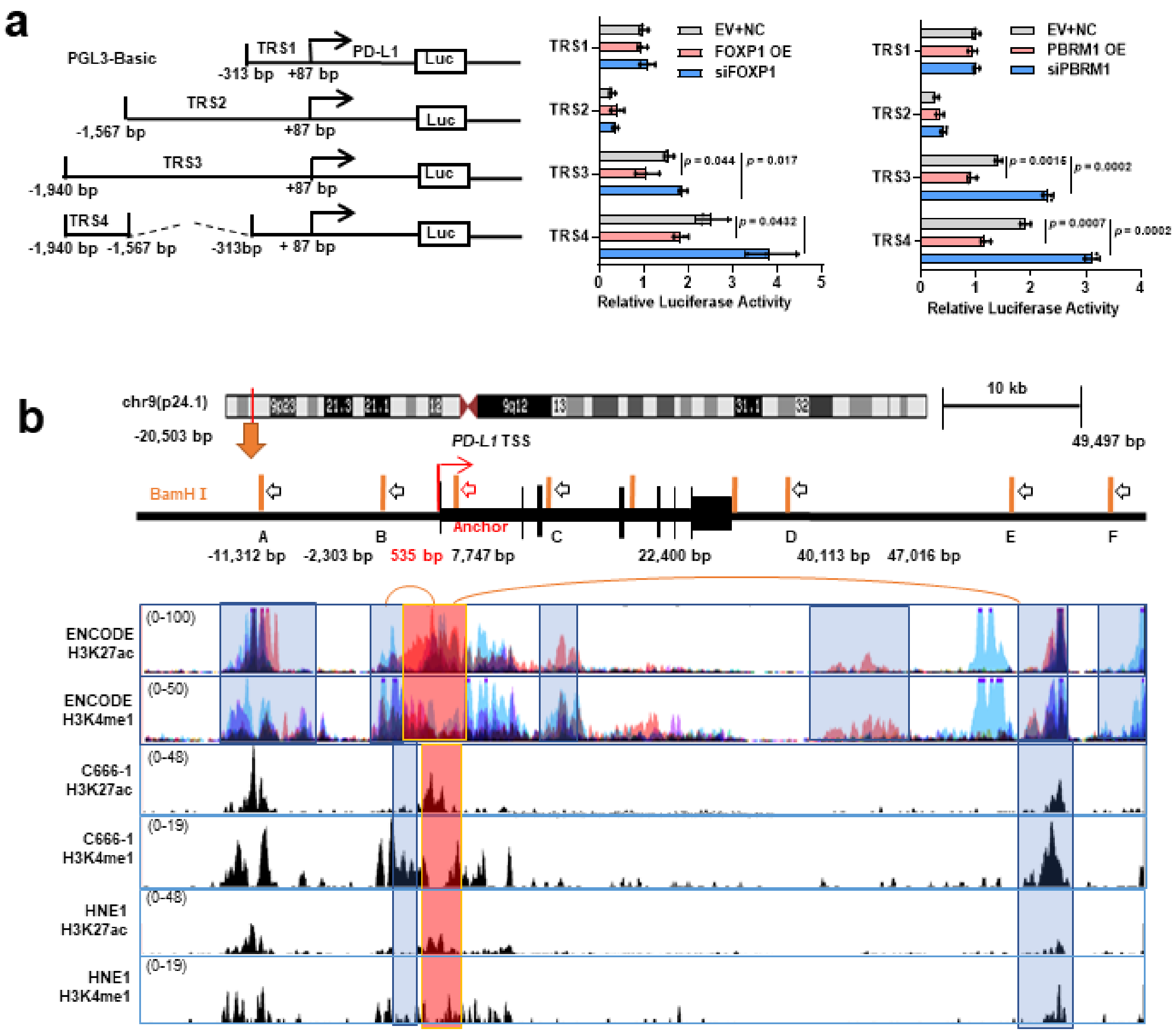

Supplementary Fig. 9 FOXP1 and PBRM1 do not inhibit *PD-L1* transcription by binding to its promoter.

a A series of luciferase reporter gene vectors were constructed based on the *PD-L1* transcription regulatory sequences (TRS) (-1,940 to +87 bp). The TRS activity was analyzed in HONE1 cells co-transfected with the corresponding TRS vector, the FOXP1 or PBRM1 overexpression vector, or siRNAs. n = 3 biologically independent samples.

b Prediction and primer design of the enhancer regions of *PD-L1* (spanning -20,503 to +49,497 bp). The H3K27ac and H3K4me1 modifications in the ENCODE database and EBV negative NPC cell line

HNE1, EBV positive cell line C666-1 were shown using the UCSC browser. Based on the transcription start site (TSS), the 3C-anchor primers (red arrow) were designed according to the BamH I restriction site closest to the *PD-L1* promoter (red area).

Data are presented as mean ± s.d, and calculated by unpaired two-sided *t*-test. Source data are provided as a Source Data file.

Supplementary Fig. 10

a

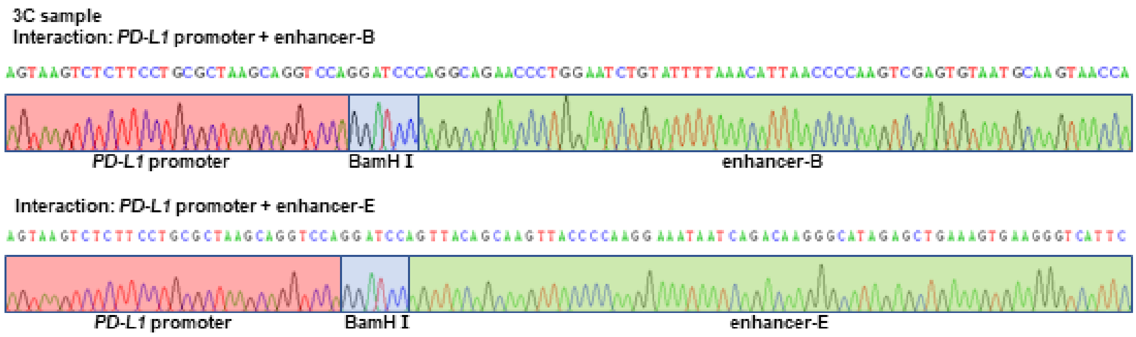

b

FOXP1 binding motif

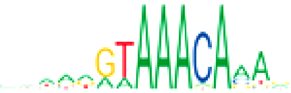

FOXP1 binding site	Predicted sequence	Relative score	Mutated sequence
B (-1,709 – -1,695 bp)	AAAACAAAAAACAAAA	0.880820836536	CCCCACCCCCACCCC
E (43,285 – 43,296 bp)	ACGAAAACATGA	0.807774638265	CATCCCCACGTC

c

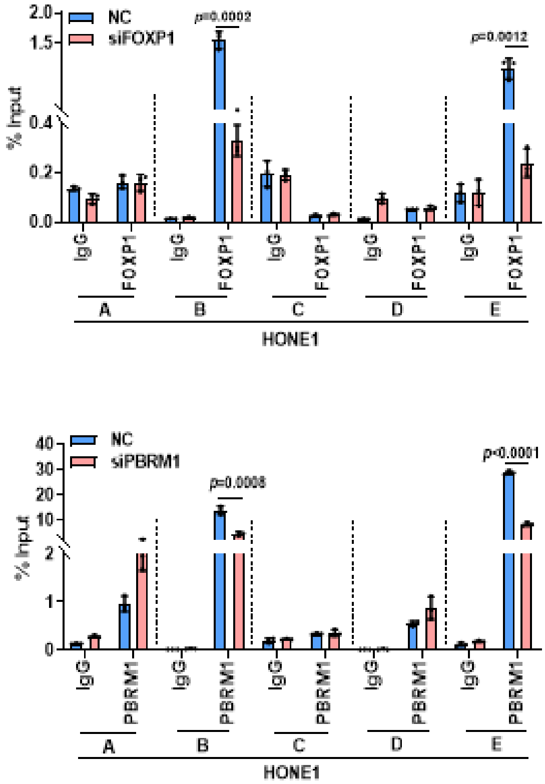

d

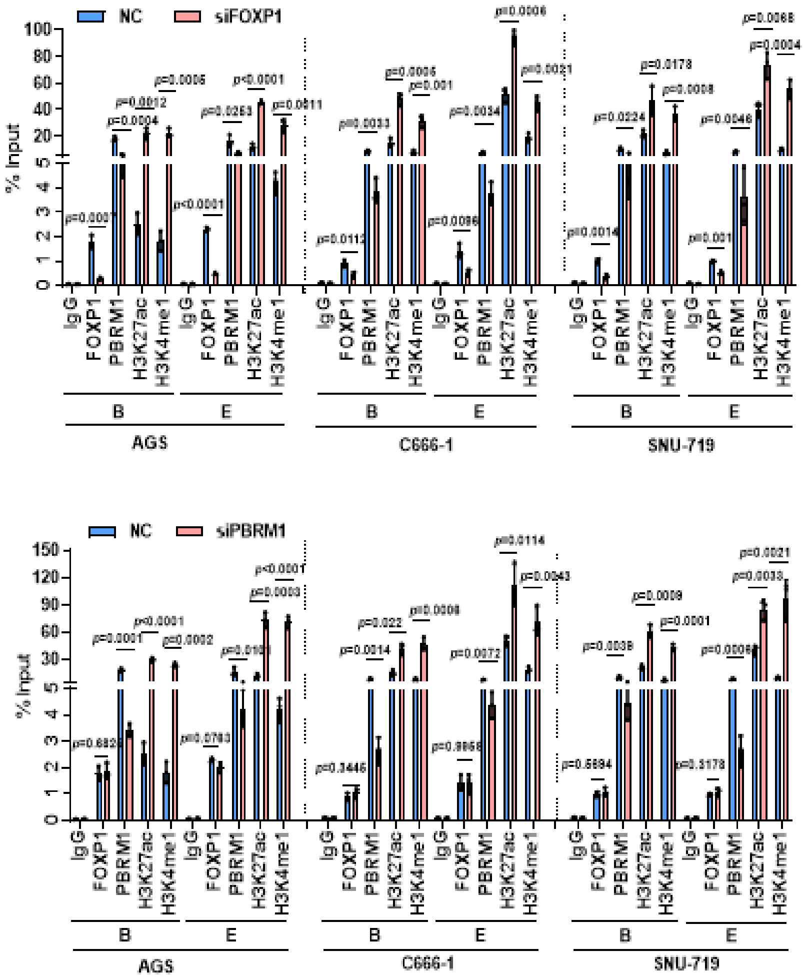

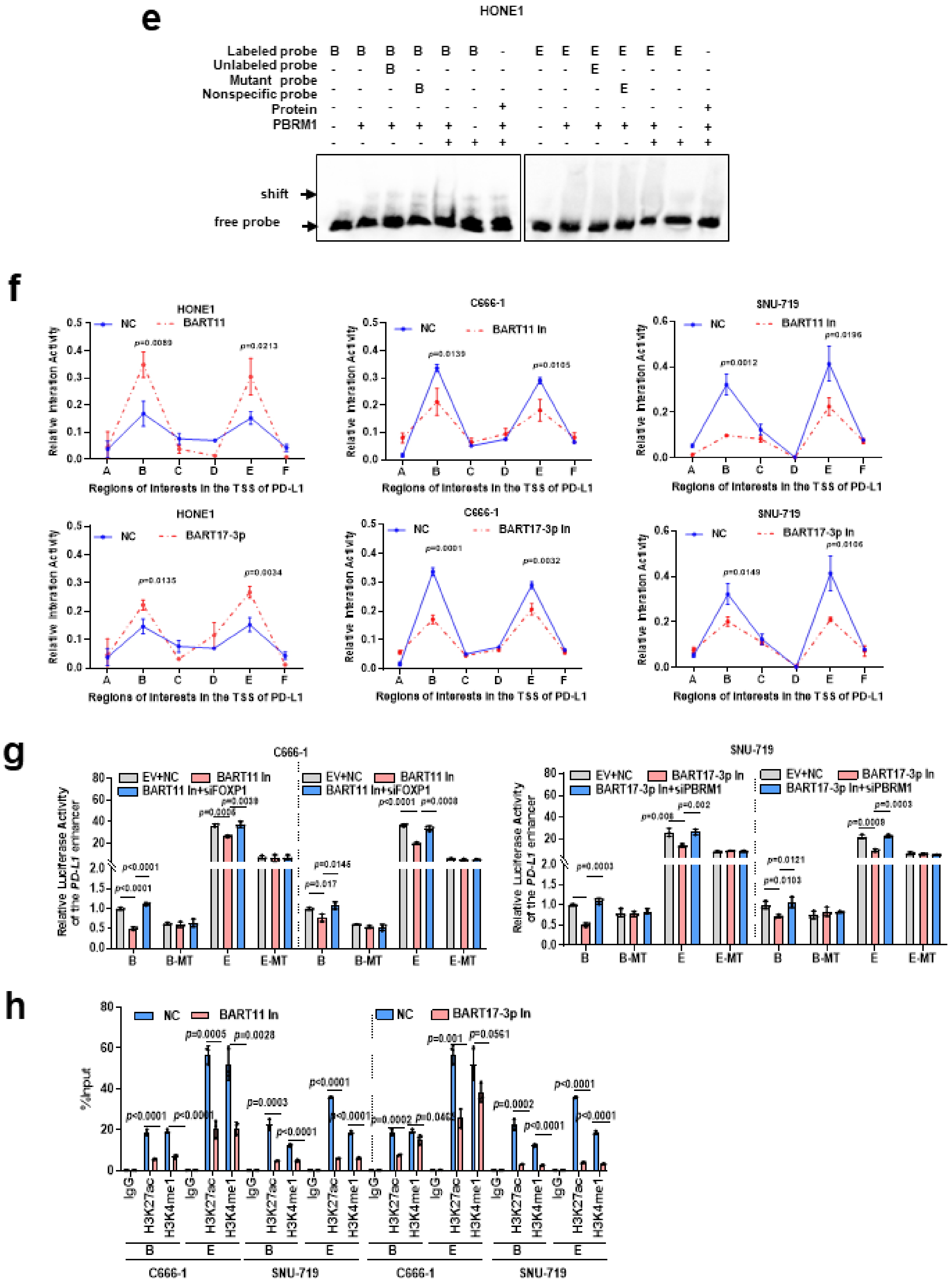

e
HONE1
Labeled probe
Unlabeled probe
Mutant probe
Nonspecific probe
Protein
PBRM1
shift
free probe
f
HONE1
C666-1
SNU-719
NC
BART11
BART11 In
BART17-3p
BART17-3p In
Relative Interation Activity
Regions of Interests in the TSS of PD-L1
g
C666-1
SNU-719
EV+NC
BART11 In
BART11 In+siFOXP1
BART17-3p In
BART17-3p In+siPBRM1
Relative Luciferase Activity of the PD-L1 enhancer
B
B-MT
E
E-MT
h
NC
BART11 In
BART17-3p In
%Input
IgG
H3K27ac
H3K4me1
C666-1
SNU-719

Supplementary Fig. 10 EBV-miR-BART11 and EBV-miR-BART17-3p regulate the *PD-L1* enhancer B and E regions by inhibiting FOXP1 and PBRM1, respectively.

a Sequencing verification of samples from the 3C experiments. Remote interactions were identified between the *PD-L1* enhancer regions B, E, and the *PD-L1* promoter regions. The blue area is the junction of the BamH I restriction site, the red area is the *PD-L1* promoter area, and the green area is the *PD-L1* enhancer area.

b The FOXP1 binding site was predicted in the enhancer B and E of *PD-L1* by JASPAR. The table lists the location of binding sites, sequences, relative scores, and mutation sequences.

c ChIP experiments using the antibodies against FOXP1 or PBRM1 were performed to identify whether knockdown of *FOXP1* or *PBRM1* affected the binding of FOXP1 and PBRM1 in the *PD-L1* enhancers A, B, C, D, and E in HONE1 cells. n = 3 biologically independent samples.

d ChIP experiments using the antibodies against FOXP1, PBRM1, H3K27ac, and H3K4me1 were performed to identify whether knockdown of *FOXP1* or *PBRM1* affected the binding of FOXP1 and PBRM1, and the H3K27ac and H3K4me1 modification in the *PD-L1* enhancers B and E in AGS, C666-1, and SNU-719 cells transfected with *FOXP1* or *PBRM1* siRNA. n = 3 biologically independent samples.

e The EMSA assay was used to detect whether PBRM1 binds to the *PD-L1* enhancers B and E in HONE1. Lane 1: only biotin-labeled probes were added; lane 2: nuclear protein and biotin-labeled region probes were added; lane 3: nuclear protein was added, biotin-labeled region probes and competitively bound unlabeled region probes were added in a ratio of 1:2; lane 4: nuclear protein was added and biotin-labeled probes and mutant unlabeled probes in a ratio of 1:2 were added; lane 5: nuclear protein, biotin-labeled probes and anti-PBRM1 antibodies were added simultaneously; lane 6: biotin-labeled probes and anti-PBRM1 antibody were added; lane 7: nuclear protein, nonspecific probe, and anti- PBRM1 antibody were added simultaneously.

f The 3C experiments were performed in HONE1, C666-1, and SNU-719 cells transfected with EBV-miR-BART11 or EBV-miR-BART17-3p mimics or inhibitors to detect the interaction frequency between the enhancers and the *PD-L1* promoter. The relative interaction frequency was normalized to the closest BamH I digestion site. n = 3 biologically independent samples.

g Luciferase reporter gene assays showed that EBV-miR-BART11 or EBV-miR-BART17-3p affects the reporter activities of the *PD-L1* enhancer B and E regions *via* FOXP1 or PBRM1 in C666-1 and SNU-719 cells co-transfected with the B and E wild-types or the corresponding mutant vectors, and EBV-miR-BART11 or EBV-miR-BART17-3p inhibitors. n = 3 biologically independent samples.

h The modification of H3K27ac and H3K4me1 in C666-1 and SNU-719 cells was analyzed by ChIP experiments after transfection of EBV-miR-BART11 or EBV-miR-BART17-3p mimics or inhibitors. n = 3 biologically independent samples.

Data are presented as mean ± s.d, and calculated by unpaired two-sided *t*-test in c, d, f, h. Source data are provided as a Source Data file.

Supplementary Fig. 11

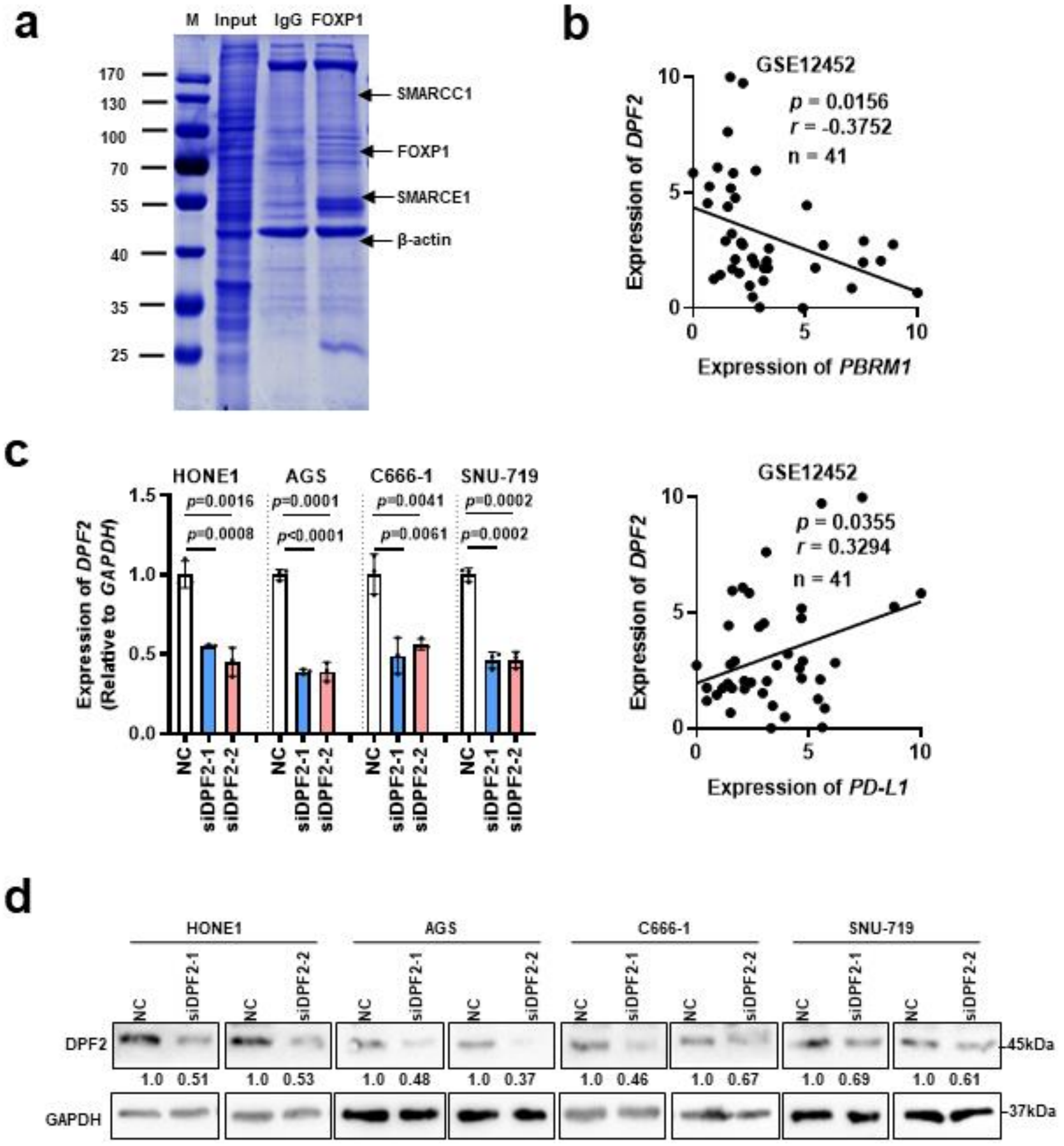

e

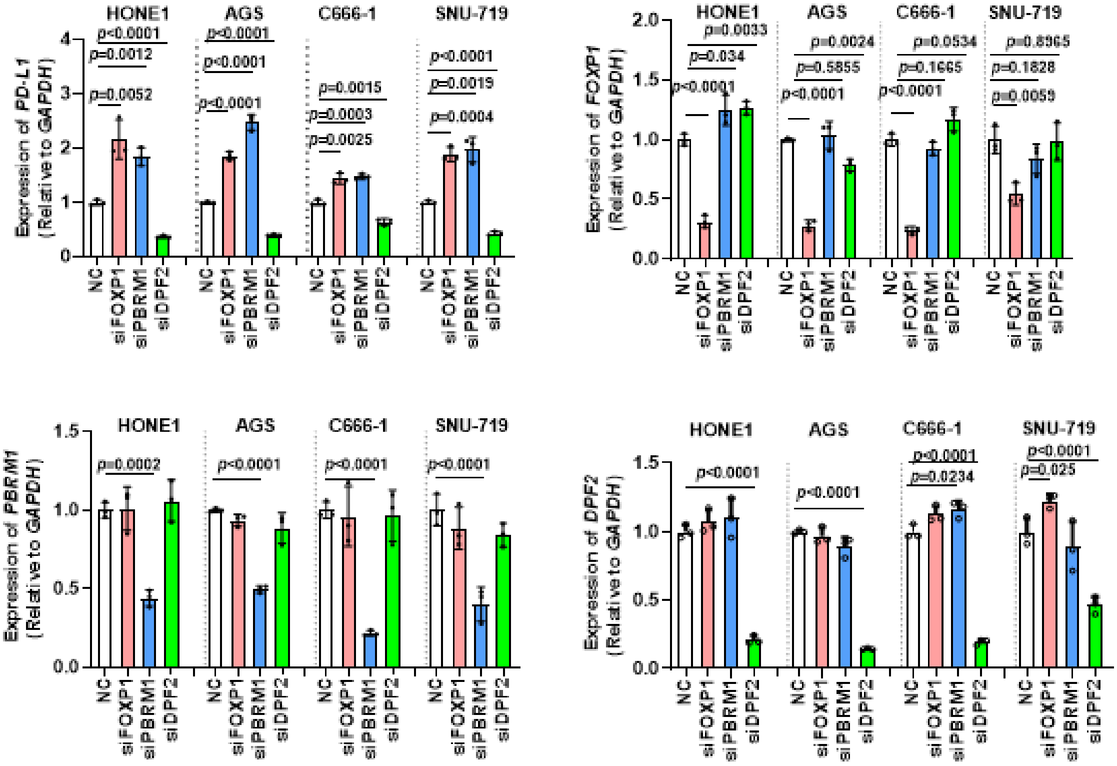

f

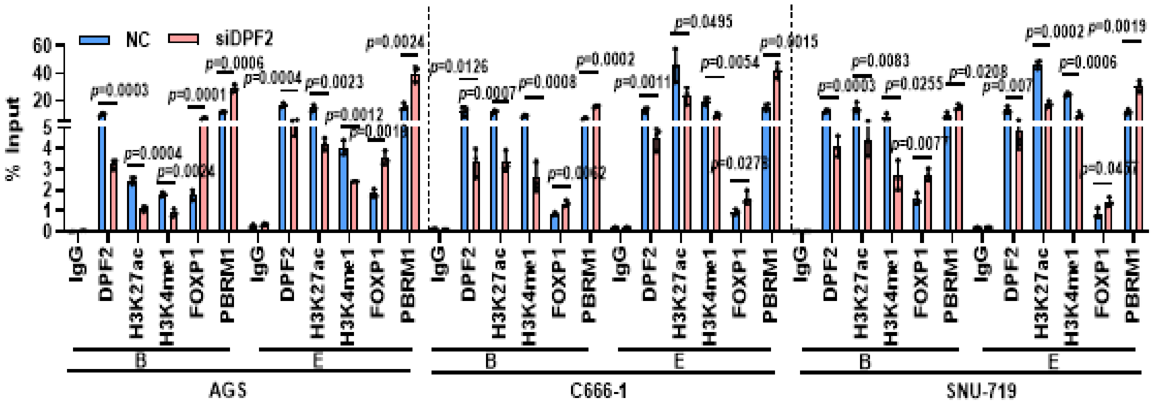

Supplementary Fig. 11 FOXP1 interacts with PBRM1 of the PBAF complex and inhibits PD-L1 expression.

a The immunoprecipitate obtained using anti-FOXP1 antibody was resolved by SDS-PAGE and stained with Coomassie brilliant blue. The molecular weight is indicated on the left side of the figure.

b The correlation between *DPF2* and *PD-L1* expression and between *DPF2* and *PBRM1* expression was analyzed using the data from GSE12452.

c qRT-PCR was used to detect the mRNA for *DPF2* in HONE1, AGS, C666-1, and SNU-719 cells after knockdown of *DPF2*. *GAPDH* was used as an internal control. n = 3 biologically independent samples.

d Western blotting was used to detect the expression for *DPF2* in HONE1, AGS, C666-1, and SNU-719 cells after knockdown of *DPF2*. *GAPDH* was used as an internal.

e qRT-PCR was used to detect the mRNA for *PD-L1*, *FOXP1*, *PBRM1*, and *DPF2* in HONE1, AGS, C666-1, and SNU-719 cells after knockdown of *FOXP1*, *PBRM1*, or *DPF2*. *GAPDH* was used as an internal control. n = 3 biologically independent samples.

f ChIP experiments using antibodies against DPF2, H3K27ac, H3K4me1, FOXP1, and PBRM1 was performed to examine whether the knockdown of *DPF2* affects the binding of DPF2, H3K27ac, H3K4me1, FOXP1, and PBRM1 in the *PD-L1* enhancer regions B and E, and the H3K27ac and H3K4me1 modification in AGS, C666-1, and SNU-719 cells. n = 3 biologically independent samples.

Data are presented as mean ± s.d, and calculated by unpaired two-sided *t*-test in c, e, f. b are calculated by *linear regression*. Source data are provided as a Source Data file.

Supplementary Fig. 12

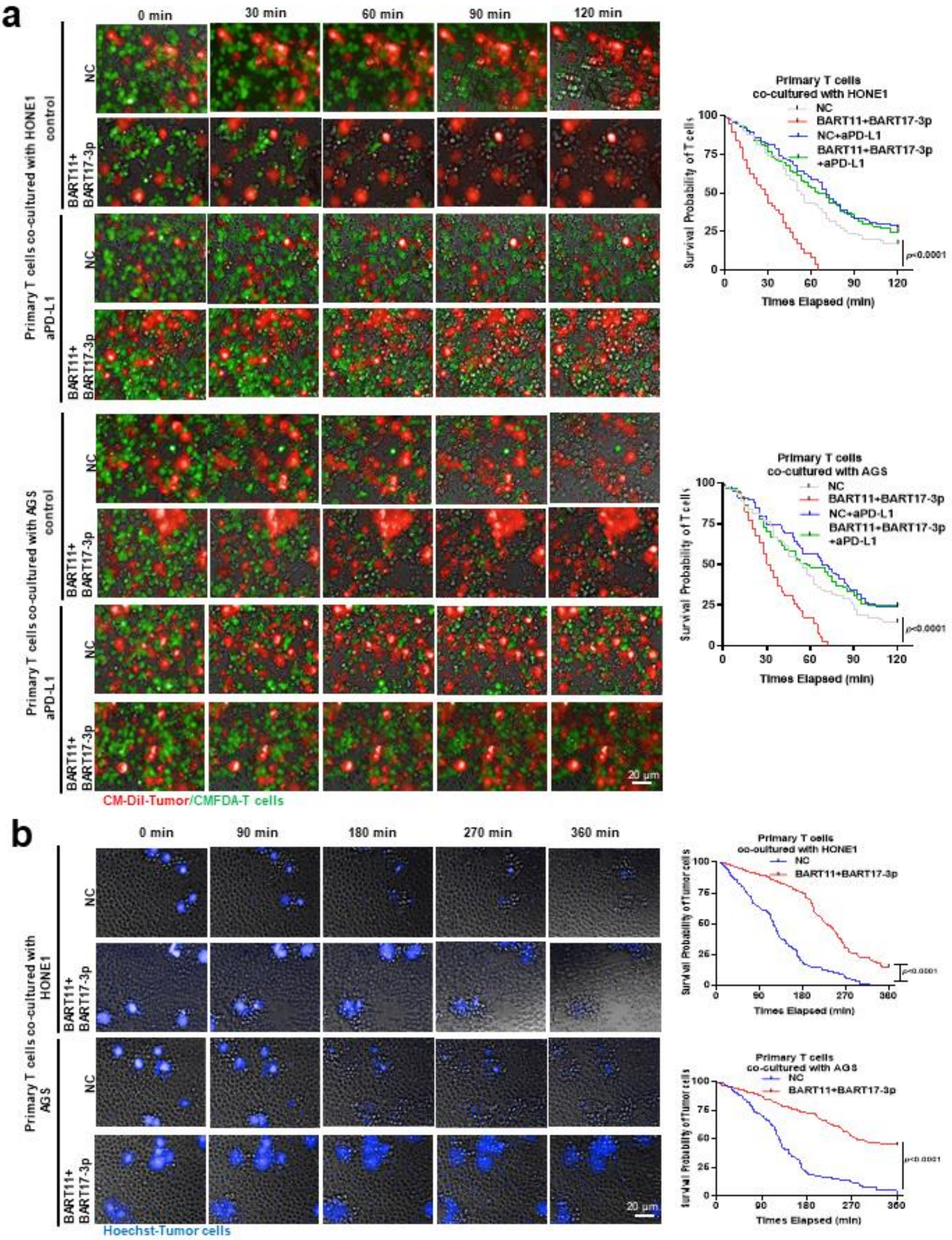

Supplementary Fig. 12 EBV-miR-BART11 and EBV-miR-BART17-3p induce T-cell apoptosis by promoting PD-L1 expression in NPC and gastric carcinoma cells.

a A high-content screening system was used to track the activity status of primary T cells. The primary T cells were co-cultured with HONE1 or AGS cells after overexpression of EBV-miR-BART11 and EBV-miR-BART17-3p in the presence of PD-L1 blocking antibody; living tumor cells: red (CM-DiI), living T cells: green (CMFDA), living and apoptotic cells: bright field. The picture on the left shows the superimposed signals for red fluorescence, green fluorescence, and bright field. The statistical result is shown on the right. Magnification: 400×, scale bars = 20 μm.

b A high-content screening system was used to track the activity status of tumor cells. EBV-negative HONE1 or AGS cells were transfected with EBV-miR-BART11 and EBV-miR-BART17-3p mimics simultaneously and were subsequently co-cultured with primary T cells. Living tumor cells: blue (Hoechst), living and apoptotic cells: bright field. The picture on the left shows the superimposed signals for blue fluorescence and bright field. The statistical graph is shown on the right. Magnification: 400×, scale bars = 20 μm.

a, b are calculated by unpaired two-sided *t*-test. Source data are provided as a Source Data file.

Supplementary Fig. 13

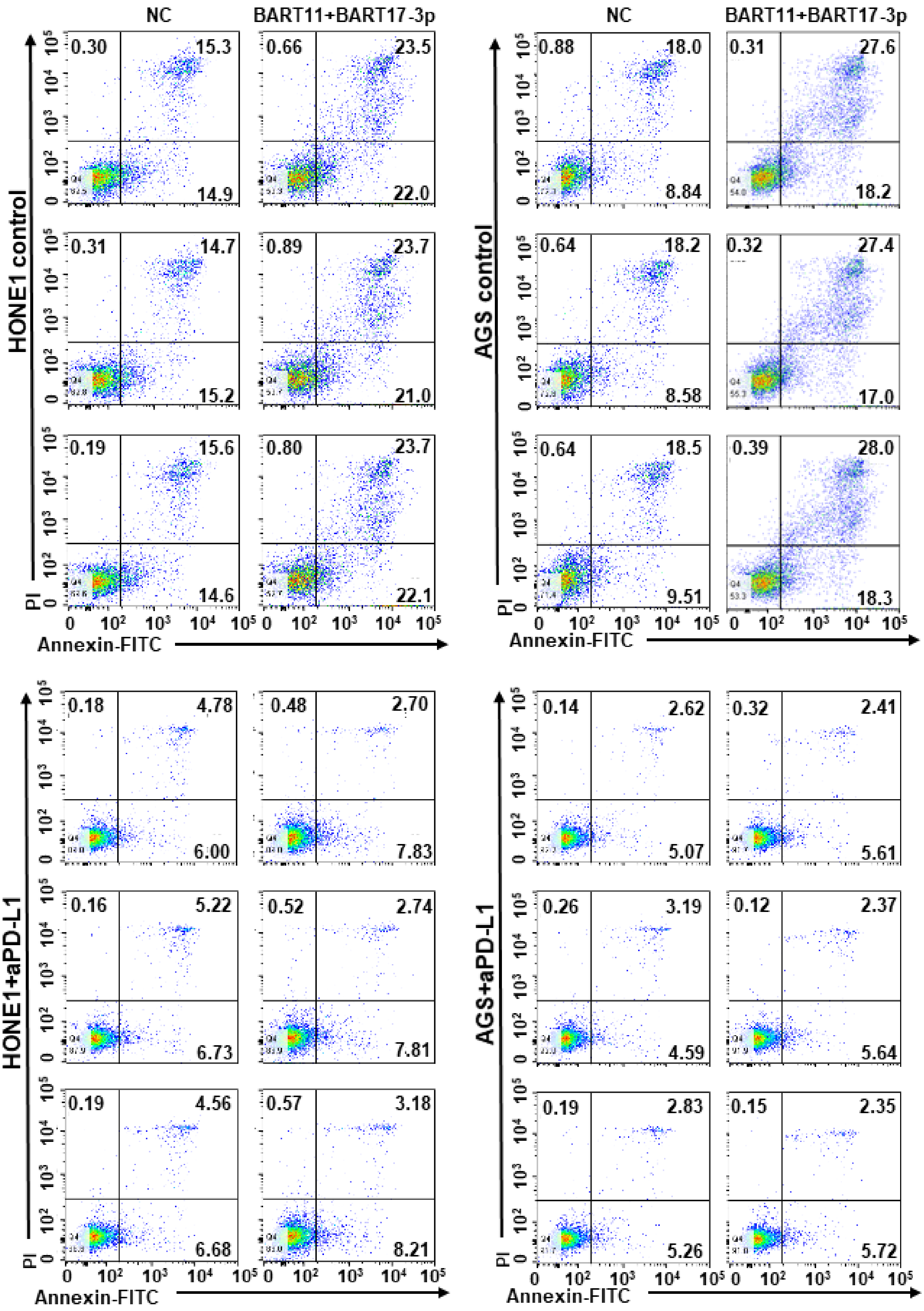

presence of PD-L1 blocking antibody. n = 3 biologically independent samples.

Supplementary Fig. 13 Original flow cytometry results for Fig. 7b. Primary T cells were co-cultured with HONE1 or AGS cells after overexpression of EBV-miR-BART11 and EBV-miR-BART17-3p in the presence of PD-L1 blocking antibody. n = 3 biologically independent samples.

Supplementary Fig. 14

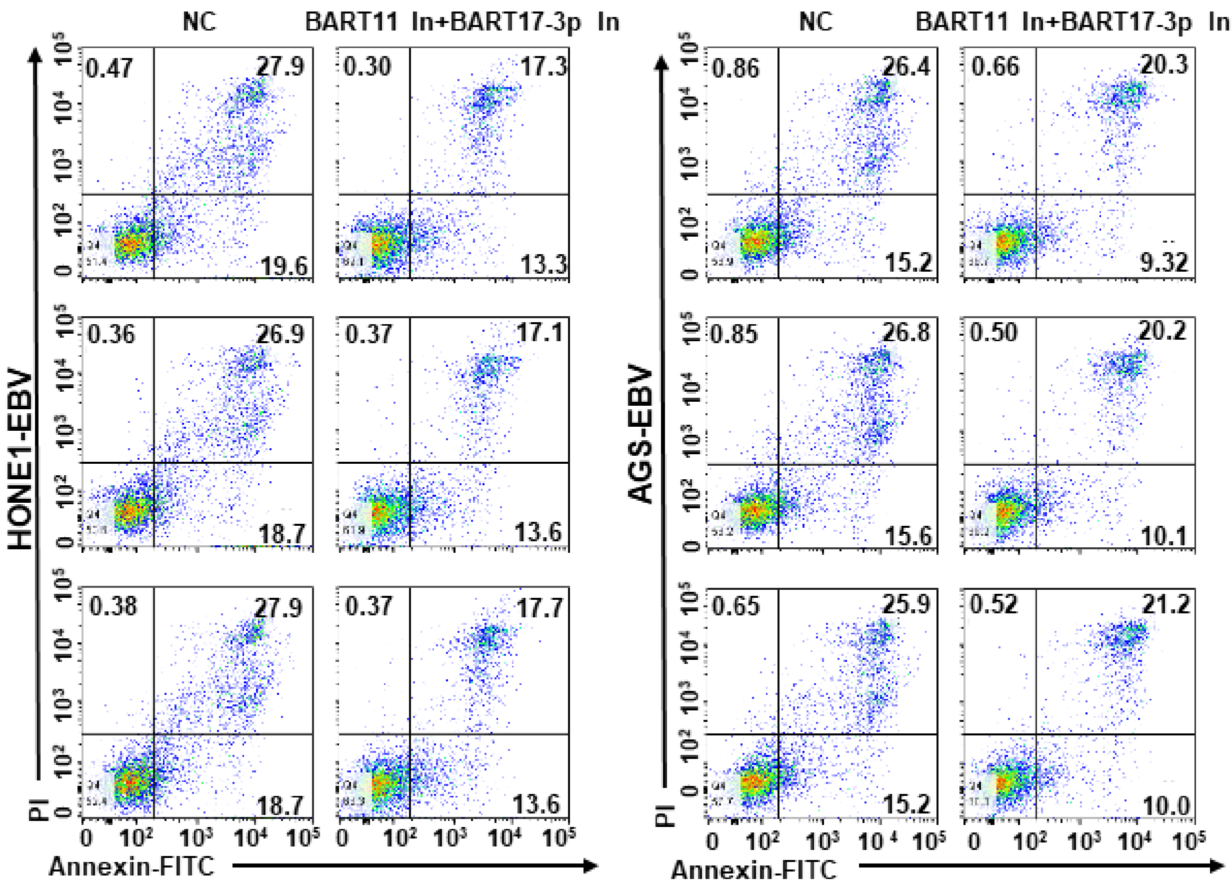

Supplementary Fig. 14 Original flow cytometry results for Fig. 7b. Primary T cells were co-cultured with HONE1-EBV or AGS-EBV after inhibition of EBV-miR-BART11 and EBV-miR-BART17-3p. n = 3 biologically independent samples.

Supplementary Fig. 15

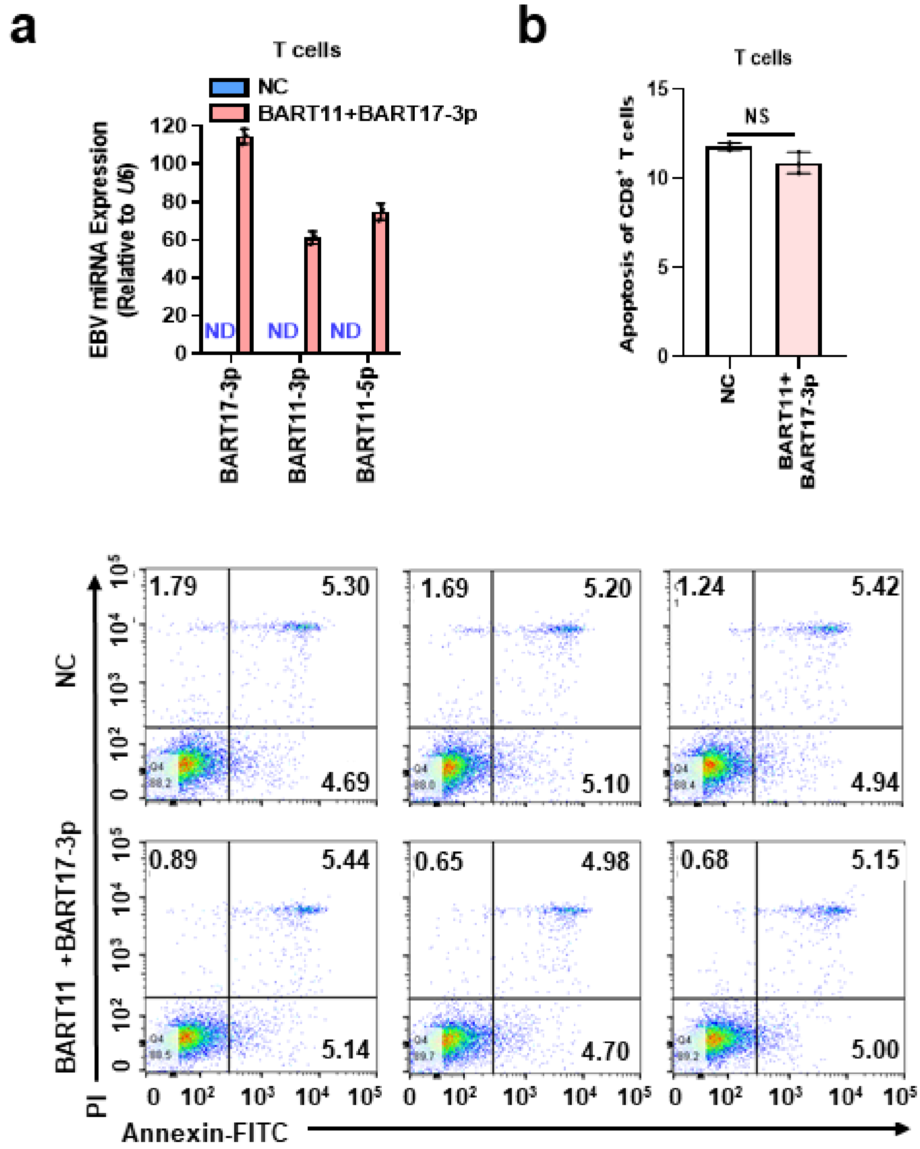

Supplementary Fig. 15 EBV-miR-BART11 and EBV-miR-BART17-3p have no effect on the degree of T-cell apoptosis.

a Primary T cells were transfected with EBV-miR-BART11 and EBV-miR-BART17-3p mimics. qRT-PCR was used to confirm the transfection efficiency. n = 3 biologically independent samples.

b Flow cytometric analysis of T-cell apoptosis in T cells transfected with EBV-miR-BART11 and EBV-miR-BART17-3p mimics. Each group was analyzed using three independent replicates.

a, b are calculated by unpaired two-sided *t*-test. Source data are provided as a Source Data file.

Supplementary Fig. 16

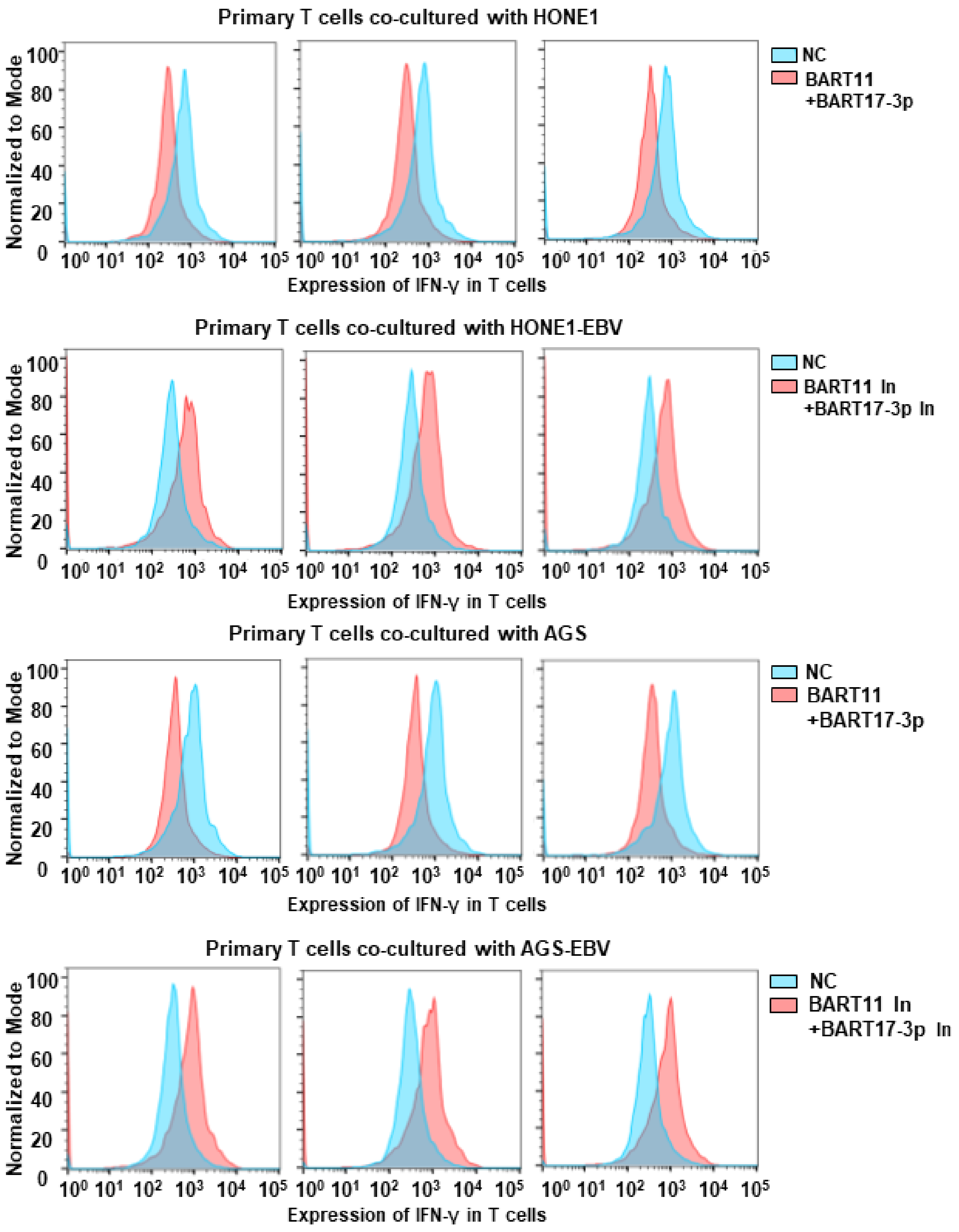

Supplementary Fig. 16 Original flow cytometry results for Fig. 7e for detecting IFN-γ secretion. Primary T cells were co-cultured with HONE1 or AGS cells after overexpression of EBV-miR-BART11 and EBV-miR-BART17-3p, or co-cultured with HONE1-EBV or AGS-EBV cells after inhibition of EBV-miR-BART11 and EBV-miR-BART17-3p. n = 3 biologically independent samples.

Supplementary Fig. 17

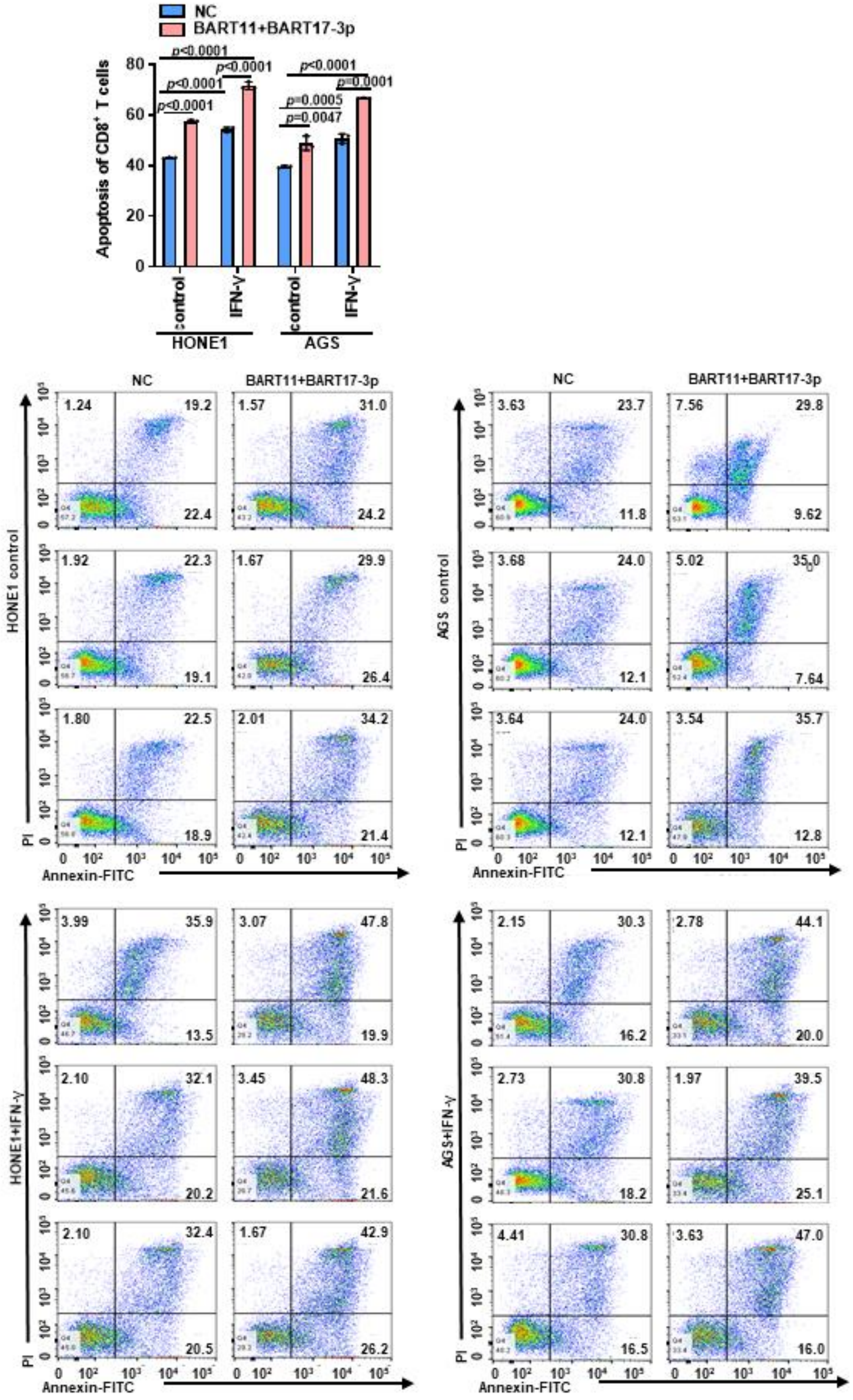

Supplementary Fig. 17 Flow cytometric analysis of T-cell apoptosis in HONE1 and AGS cells treated with IFN-γ (10 ng/mL) and EBV-miR-BART11 and EBV-miR-BART17-3p mimics. Each group was analyzed using three independent replicates. Data are presented as mean ± s.d, and, *p* values are calculated by unpaired two-sided *t*-test in a-f. Source data are provided as a Source Data file.

Supplementary Fig. 18

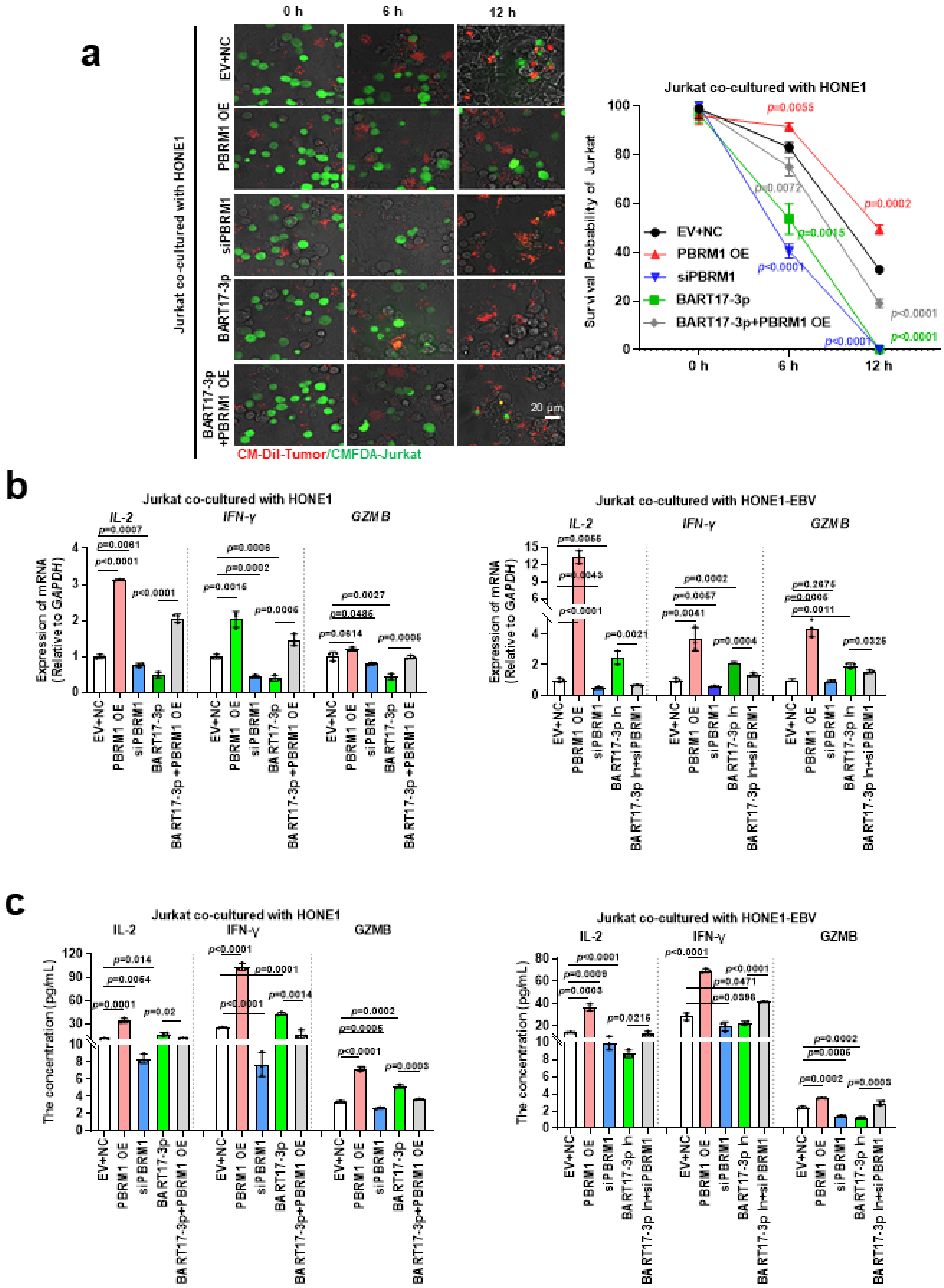

Supplementary Fig. 18 EBV-miR-BART17-3p enhances tumor cells to induce Jurkat T-cell apoptosis by inhibiting PBRM1.

a A confocal fluorescence microscope was used to show the activity status of activated Jurkat T cells. The cells were co-cultured with HONE1 cells transfected with the PBRM1 overexpression vector, siPBRM1 or EBV-miR-BART17-3p mimics, or co-transfected with EBV-miR-BART17-3p mimics and the PBRM1 overexpression vector. Living tumor cells: red (CM-DiI), living T cells: green (CMFDA), living and apoptotic cells: bright field. The picture on the left shows the superimposed signals for red fluorescence, green fluorescence, and bright field. The statistical graph is shown on the right. Magnification: 600×, scale bars = 20 μm. n = 3 biologically independent samples.

b qRT-PCR analysis for the expression of *IL-2, IFN-γ*, and *GZMB* in Jurkat cells. The cells were co-cultured with HONE1 or HONE1-EBV cells after the overexpression or inhibition of PBRM1 and EBV-miR-BART17-3p. *GAPDH* was used as an internal control. n = 3 biologically independent samples.

c ELISA analysis of the secretion of IL-2, IFN-γ, and GZMB proteins of Jurkat cells. The cells were co-cultured with HONE1 or HONE1-EBV cells after the overexpression or inhibition of PBRM1 and EBV-miR-BART17-3p. n = 3 biologically independent samples.

Data are presented as mean ± s.d, and, *p* values are calculated by unpaired two-sided *t*-test in a-c. Source data are provided as a Source Data file.

Supplementary Fig. 19

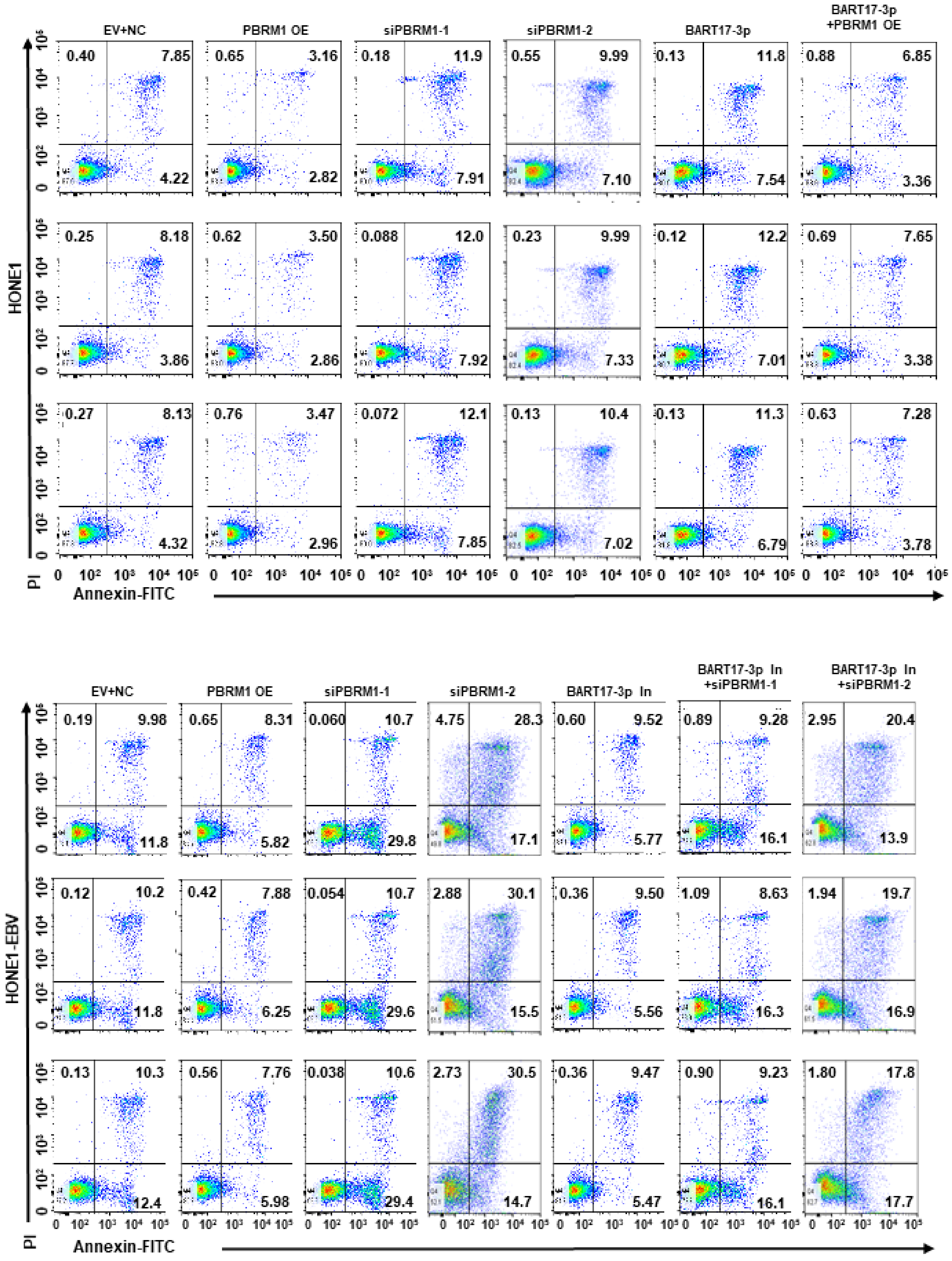

Supplementary Fig. 19 Original flow cytometry results of Fig. 7f. EBV-miR-BART17-3p can induce Jurkat cells apoptosis through inhibiting PBRM1, each group was analyzed using three independent replicates.

Supplementary Fig. 20

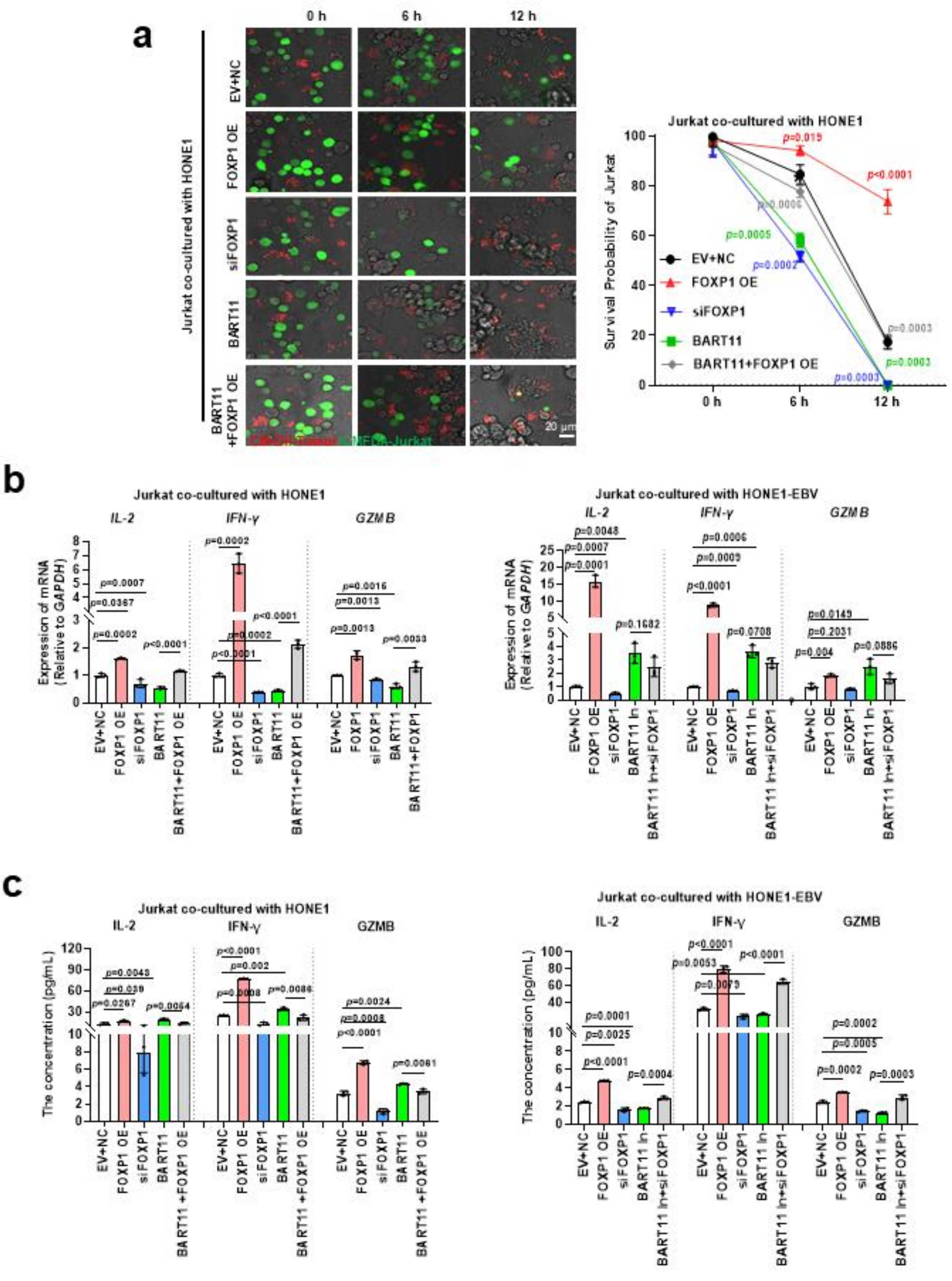

Supplementary Fig. 20 EBV-miR-BART11 enhances tumor cells to induce Jurkat T-cell apoptosis by inhibiting FOXP1.

a Confocal fluorescence microscopy was used to show the activity status of activated Jurkat T cells. The cells were co-cultured with HONE1 or HONE1-EBV cells after the overexpression or inhibition of FOXP1 and EBV-miR-BART11. Living tumor cells: red (CM-DiI), living T cells: green (CMFDA), living and apoptotic cells: bright field. Left picture: superimposed signals for red fluorescence, green fluorescence, and bright field. The statistical graph is shown on the right. Magnification: 600×, scale bars = 20 µm. n = 3 biologically independent samples.

b qRT-PCR analysis for the mRNA levels of *IL-2, IFN-γ,* and *GZMB* in Jurkat cells. The cells were co-cultured with HONE1 or HONE1-EBV cells after the overexpression or inhibition of FOXP1 and EBV-miR-BART11. *GAPDH* was used as an internal control. n = 3 biologically independent samples.

c ELISA analysis of the secretion of IL-2, IFN-γ, and GZMB proteins. Jurkat T cells were co-cultured with HONE1 or HONE1-EBV cells after the overexpression or inhibition of FOXP1 and EBV-miR-BART11. n = 3 biologically independent samples.

Data are presented as mean ± s.d, and *p* values are calculated by unpaired two-sided *t*-test in a-c. Source data are provided as a Source Data file.

Supplementary Fig. 21

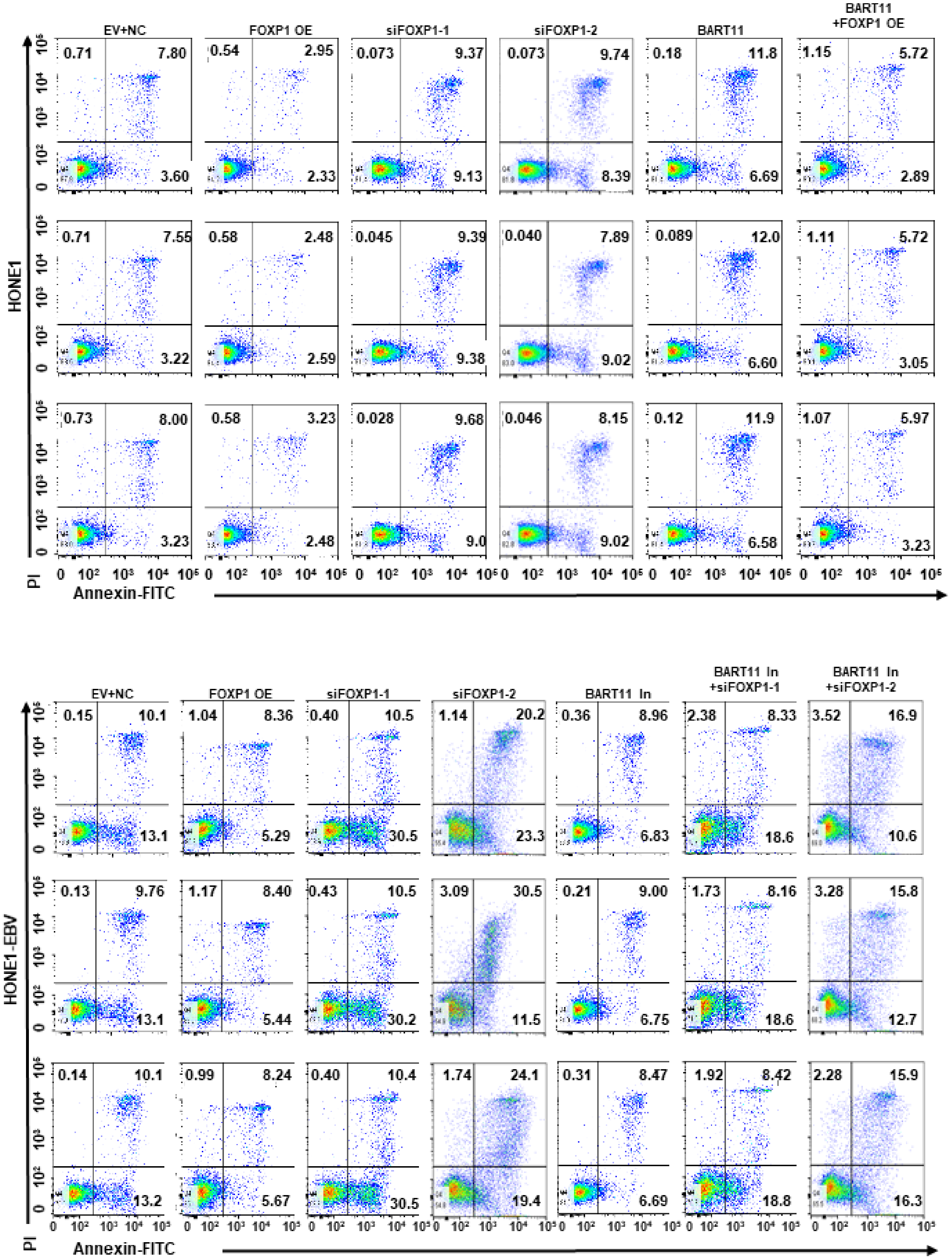

Supplementary Fig. 21 Original flow cytometry results for Fig. 7f. EBV-miR-BART11 can induce Jurkat cells apoptosis by inhibiting FOXP1. Each group was analyzed using three independent replicates.

Supplementary Fig. 22

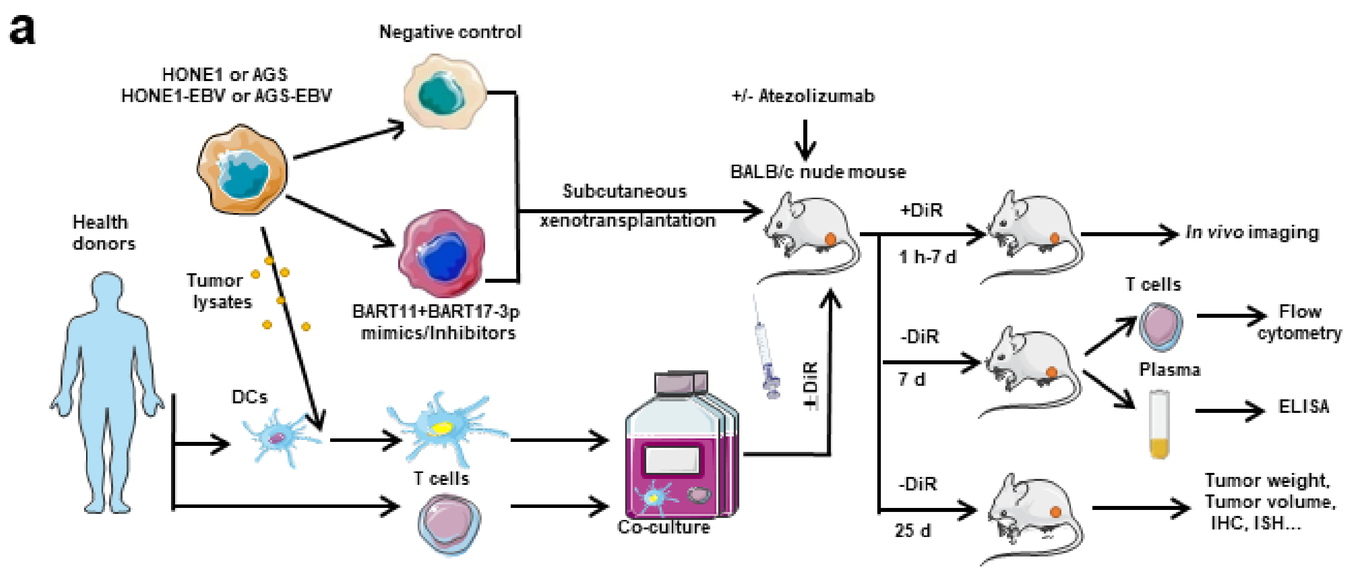

b

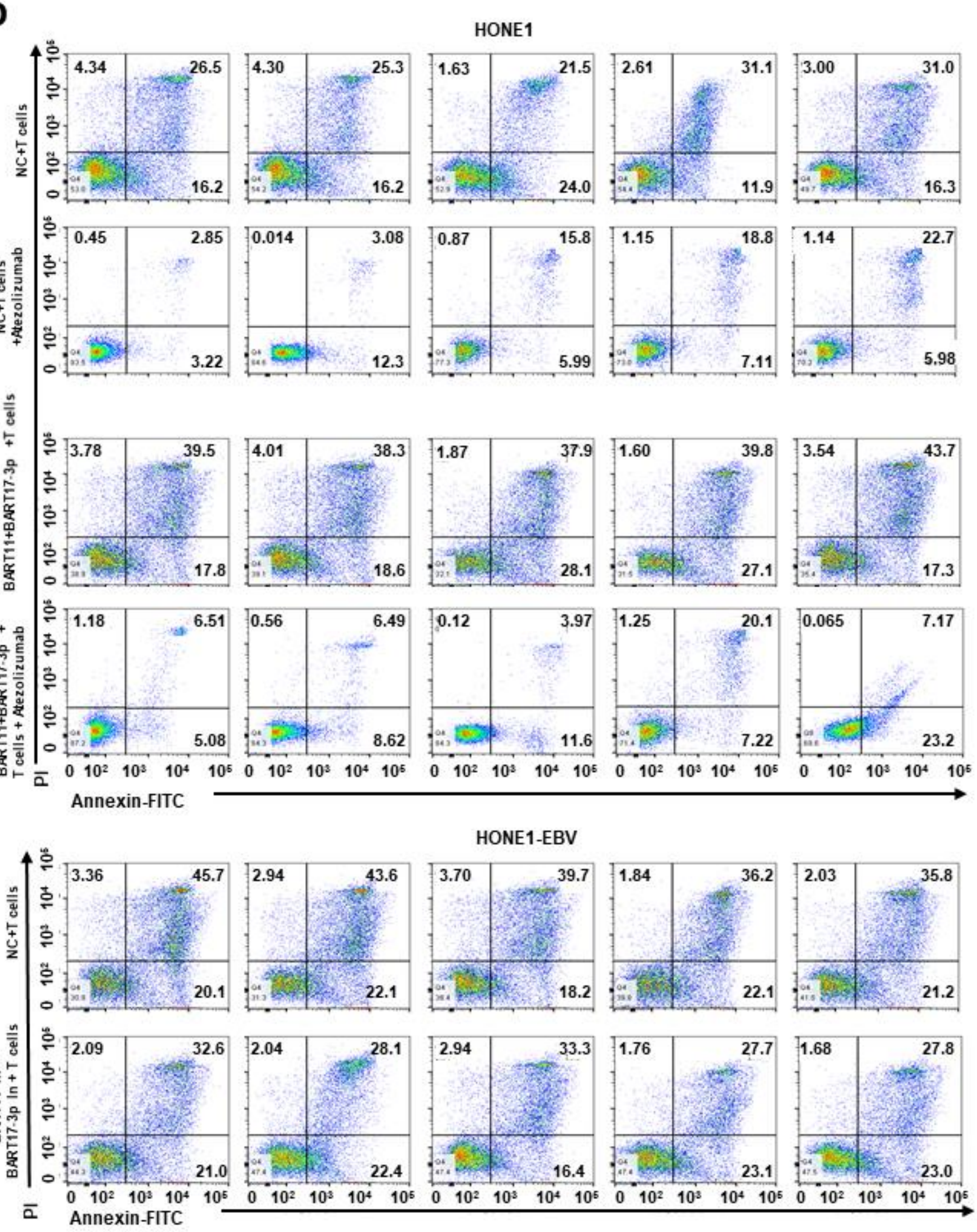
HONE1
NC+T cells
NC+T cells +Atezolizumab
BART11+BART17-3p +T cells
BART11+BART17-3p + T cells + Atezolizumab
4.34 26.5 16.2
4.30 25.3 16.2
1.63 21.5 24.0
2.61 31.1 11.9
3.00 31.0 16.3
0.45 2.85 3.22
0.014 3.08 12.3
0.87 15.8 5.99
1.15 18.8 7.11
1.14 22.7 5.98
3.78 39.5 17.8
4.01 38.3 18.6
1.87 37.9 28.1
1.60 39.8 27.1
3.54 43.7 17.3
1.18 6.51 5.08
0.56 6.49 8.62
0.12 3.97 11.6
1.25 20.1 7.22
0.065 7.17 23.2
PI
Annexin-FITC
HONE1-EBV
NC+T cells
BART11 In + BART17-3p In + T cells
3.36 45.7 20.1
2.94 43.6 22.1
3.70 39.7 18.2
1.84 36.2 22.1
2.03 35.8 21.2
2.09 32.6 21.0
2.04 28.1 22.4
2.94 33.3 16.4
1.76 27.7 23.1
1.68 27.8 23.0
PI
Annexin-FITC

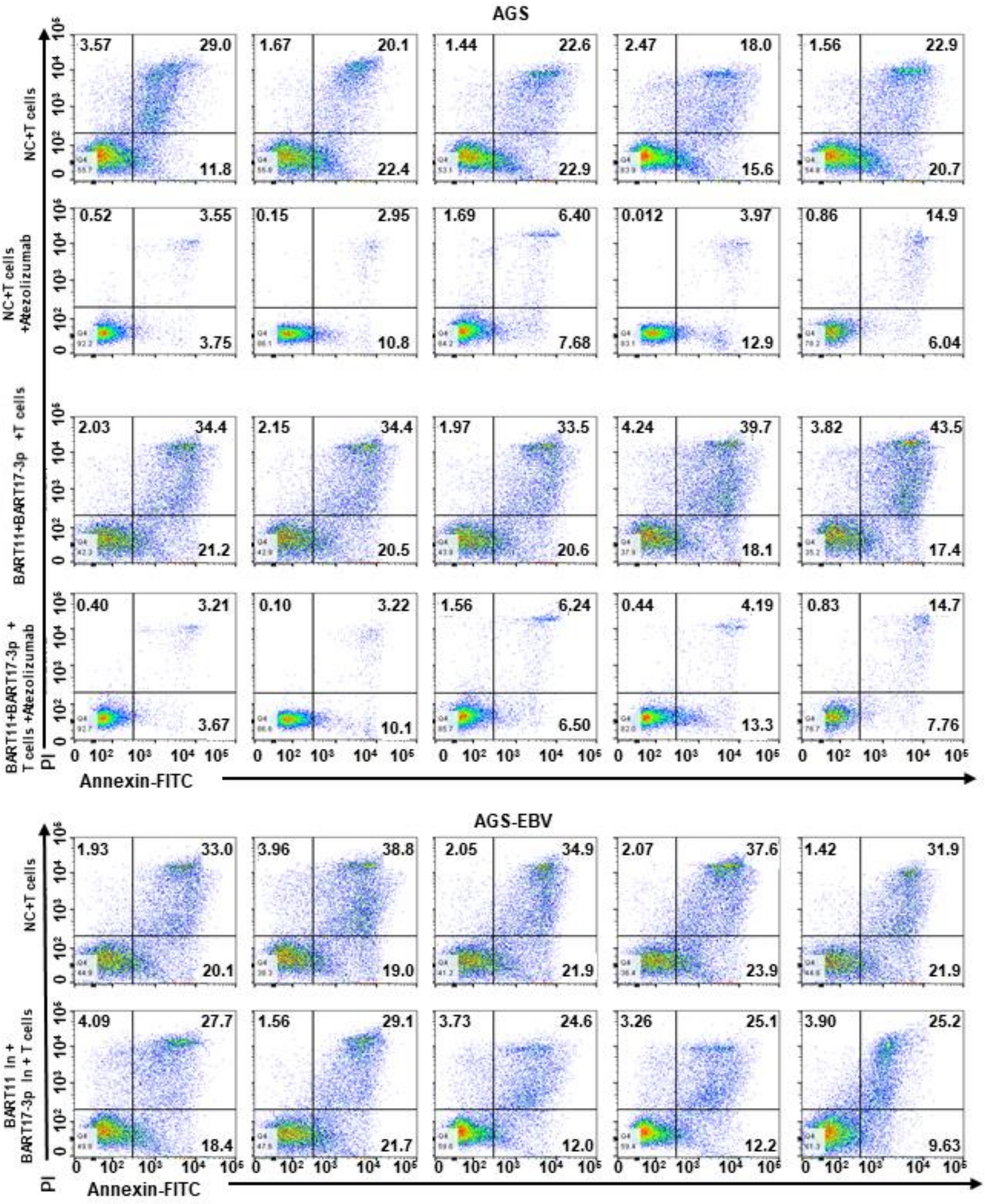
AGS
NC+T cells
3.57 29.0 11.8
1.67 20.1 22.4
1.44 22.6 22.9
2.47 18.0 15.6
1.56 22.9 20.7
NC+T cells +Atezolizumab
0.52 3.55 3.75
0.15 2.95 10.8
1.69 6.40 7.68
0.012 3.97 12.9
0.86 14.9 6.04
BART11+BART17-3p +T cells
2.03 34.4 21.2
2.15 34.4 20.5
1.97 33.5 20.6
4.24 39.7 18.1
3.82 43.5 17.4
BART11+BART17-3p + T cells +Atezolizumab
0.40 3.21 3.67
0.10 3.22 10.1
1.56 6.24 6.50
0.44 4.19 13.3
0.83 14.7 7.76
PI
Annexin-FITC
AGS-EBV
NC+T cells
1.93 33.0 20.1
3.96 38.8 19.0
2.05 34.9 21.9
2.07 37.6 23.9
1.42 31.9 21.9
BART11 In + BART17-3p In + T cells
4.09 27.7 18.4
1.56 29.1 21.7
3.73 24.6 12.0
3.26 25.1 12.2
3.90 25.2 9.63
PI
Annexin-FITC

c

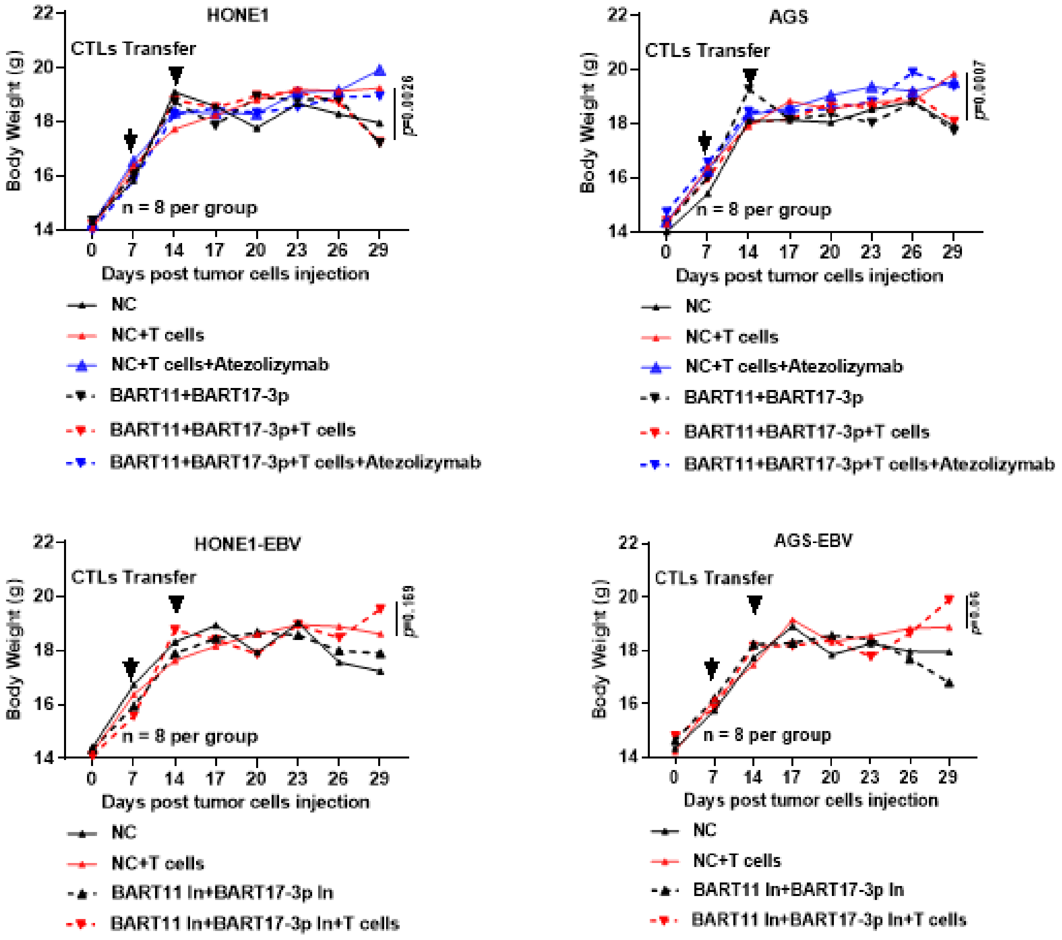
HONE1
AGS
HONE1-EBV
AGS-EBV
CTLs Transfer
Body Weight (g)
n = 8 per group
Days post tumor cells injection
NC
NC+T cells
NC+T cells+Atezolizymab
BART11+BART17-3p
BART11+BART17-3p+T cells
BART11+BART17-3p+T cells+Atezolizymab
BART11 In+BART17-3p In
BART11 In+BART17-3p In+T cells

d

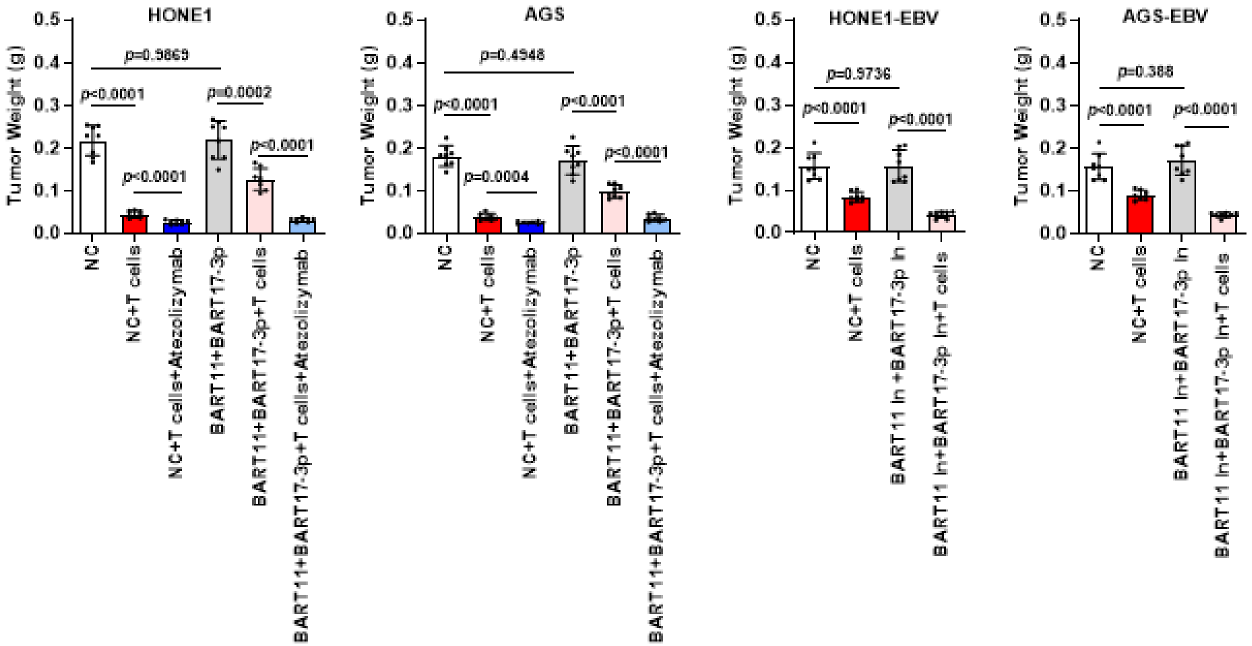
HONE1
AGS
HONE1-EBV
AGS-EBV
Tumor Weight (g)
p=0.9869
p=0.4948
p=0.9736
p=0.388
p<0.0001
p=0.0002
p=0.0004
NC
NC+T cells
NC+T cells+Atezolizymab
BART11+BART17-3p
BART11+BART17-3p+T cells
BART11+BART17-3p+T cells+Atezolizymab
BART11 In+BART17-3p In
BART11 In+BART17-3p In+T cells

e

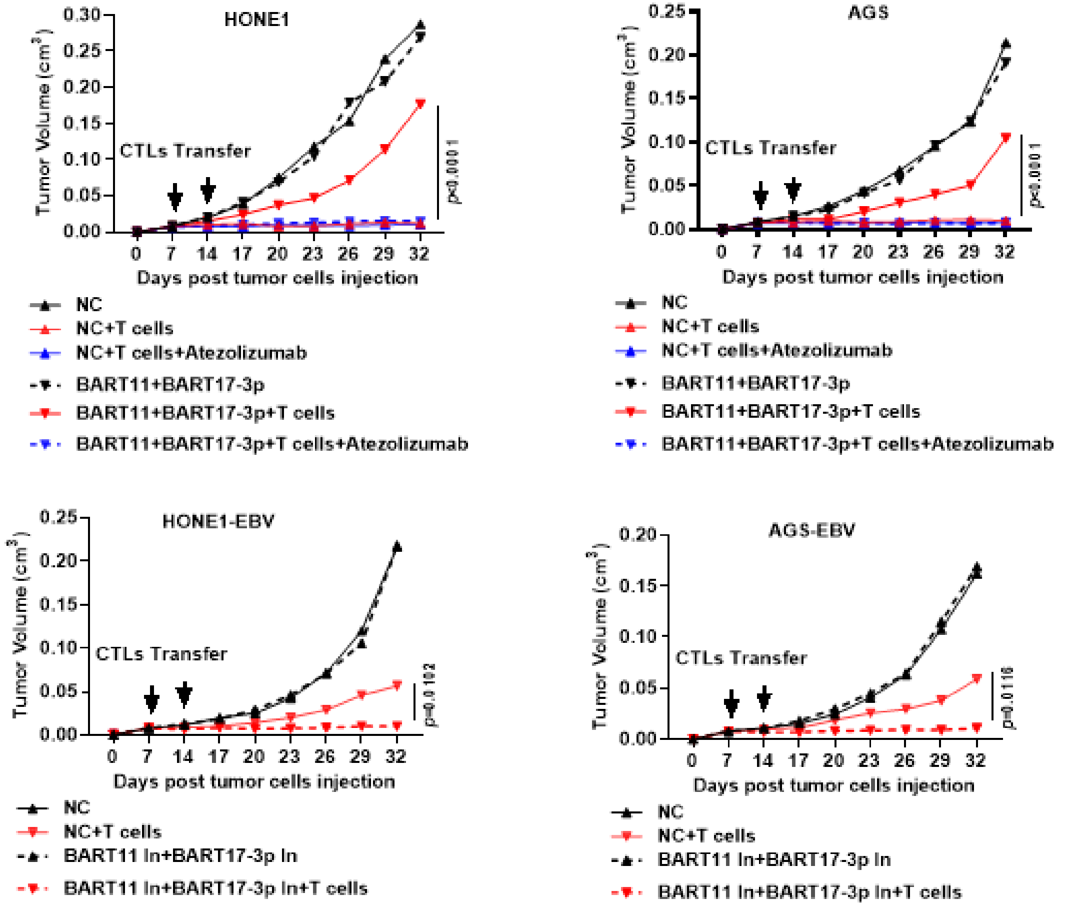
HONE1
CTLs Transfer
Tumor Volume (cm^3)
Days post tumor cells injection
p<0.0001
NC
NC+T cells
NC+T cells+Atezolizumab
BART11+BART17-3p
BART11+BART17-3p+T cells
BART11+BART17-3p+T cells+Atezolizumab
AGS
CTLs Transfer
Tumor Volume (cm^3)
Days post tumor cells injection
p<0.0001
NC
NC+T cells
NC+T cells+Atezolizumab
BART11+BART17-3p
BART11+BART17-3p+T cells
BART11+BART17-3p+T cells+Atezolizumab
HONE1-EBV
CTLs Transfer
Tumor Volume (cm^3)
Days post tumor cells injection
p=0.0102
NC
NC+T cells
BART11 In+BART17-3p In
BART11 In+BART17-3p In+T cells
AGS-EBV
CTLs Transfer
Tumor Volume (cm^3)
Days post tumor cells injection
p=0.0116
NC
NC+T cells
BART11 In+BART17-3p In
BART11 In+BART17-3p In+T cells

f

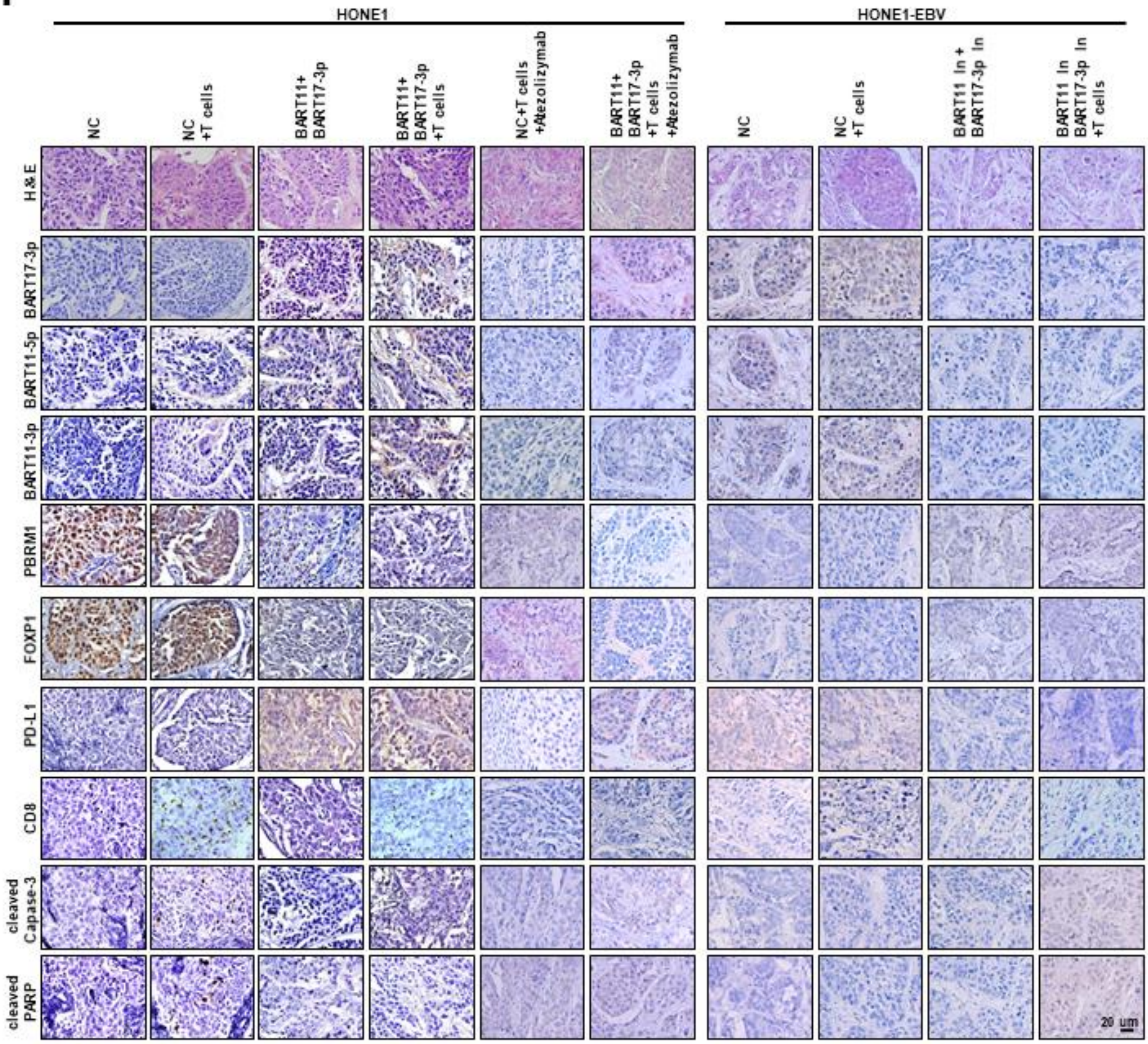
HONE1
HONE1-EBV
NC
NC +T cells
BART11+ BART17-3p
BART11+ BART17-3p +T cells
NC+T cells +Atezolizymab
BART11+ BART17-3p +T cells +Atezolizymab
NC
NC +T cells
BART11 In + BART17-3p In
BART11 In BART17-3p In +T cells
H&E
BART17-3p
BART11-5p
BART11-3p
PBRM1
FOXP1
PD-L1
CD8
cleaved Capase-3
cleaved PARP
20 um

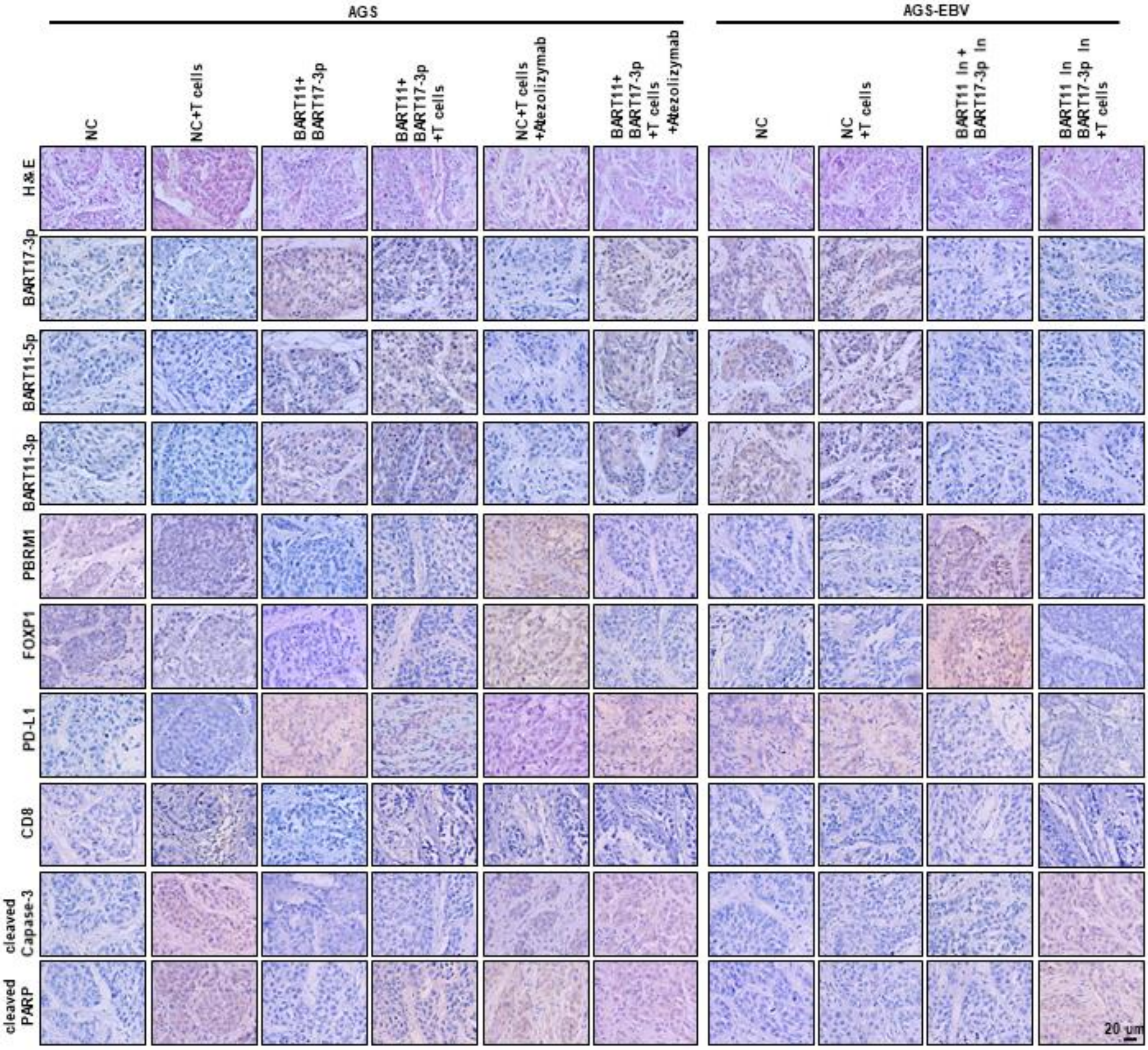
AGS
AGS-EBV
NC
NC+T cells
BART11+ BART17-3p
BART11+ BART17-3p +T cells
NC+T cells +Atezolizymab
BART11+ BART17-3p +T cells +Atezolizymab
NC
NC +T cells
BART11 In + BART17-3p In
BART11 In BART17-3p In +T cells
H&E
BART17-3p
BART11-5p
BART11-3p
PBRM1
FOXP1
PD-L1
CD8
cleaved Capase-3
cleaved PARP
20 um

g

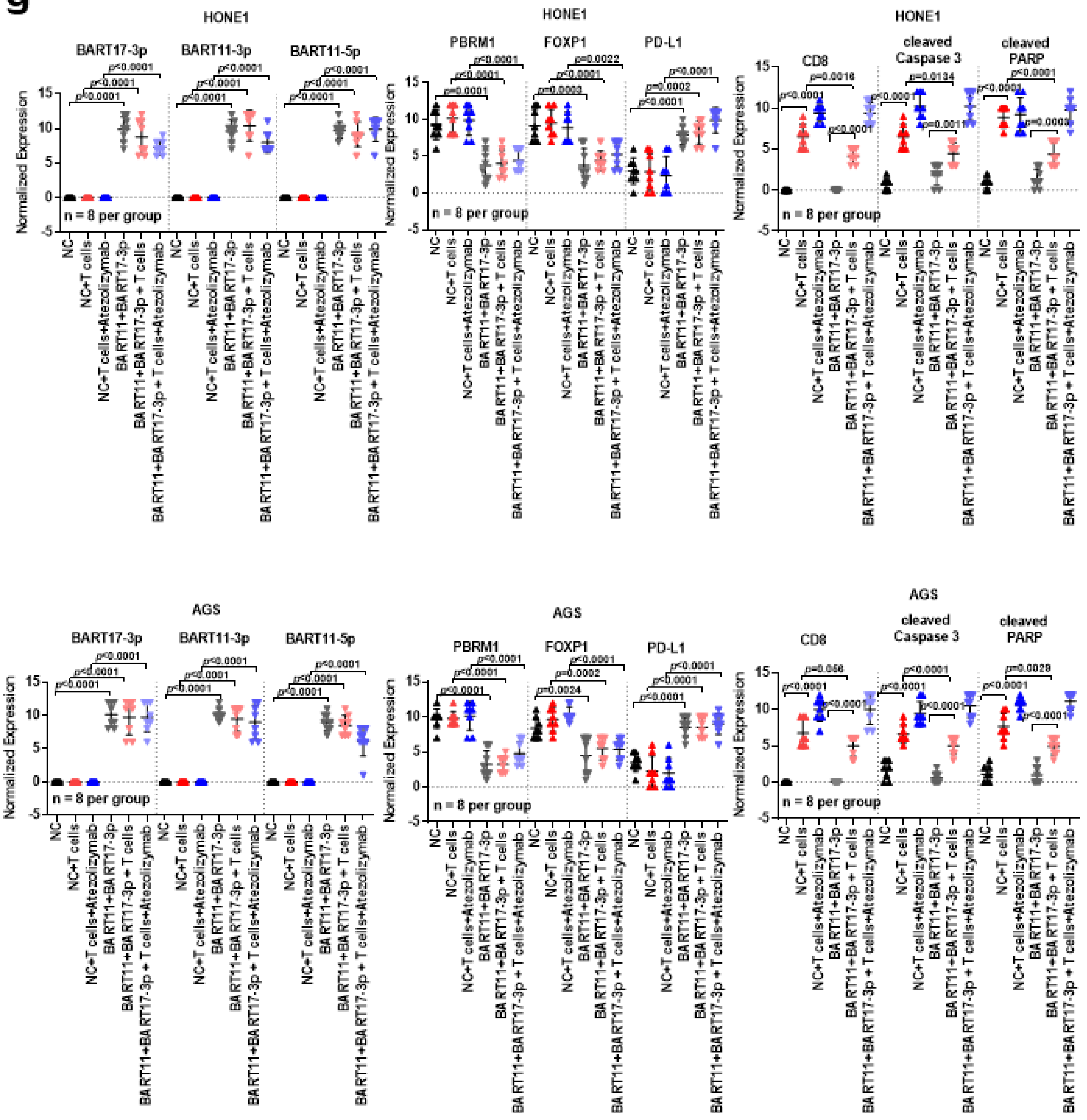

HONE1
BART17-3p
BART11-3p
BART11-5p
PBRM1
FOXP1
PD-L1
CD8
cleaved Caspase 3
cleaved PARP
AGS
Normalized Expression
n = 8 per group
NC
NC+T cells
NC+T cells+Atezolizymab
BART11+BART17-3p
BART11+BART17-3p + T cells
BART11+BART17-3p + T cells+Atezolizymab

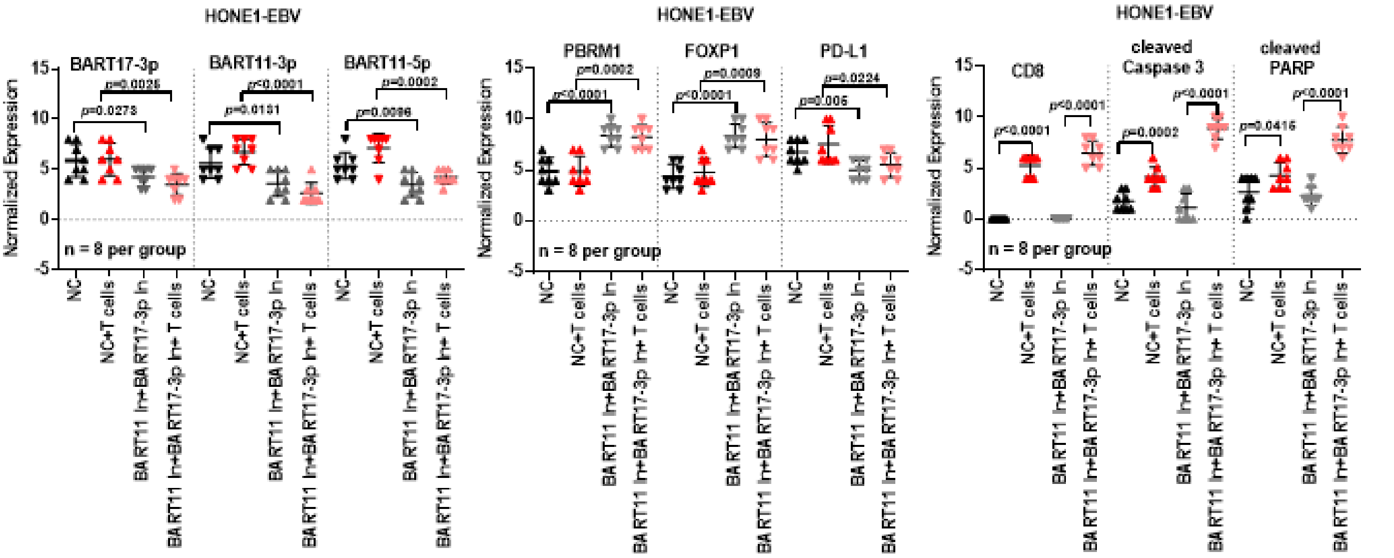
HONE1-EBV
BART17-3p
BART11-3p
BART11-5p
PBRM1
FOXP1
PD-L1
CD8
cleaved Caspase 3
cleaved PARP
Normalized Expression
n = 8 per group
NC
NC+T cells
BART11 In+BART17-3p In
BART11 In+BART17-3p In+ T cells

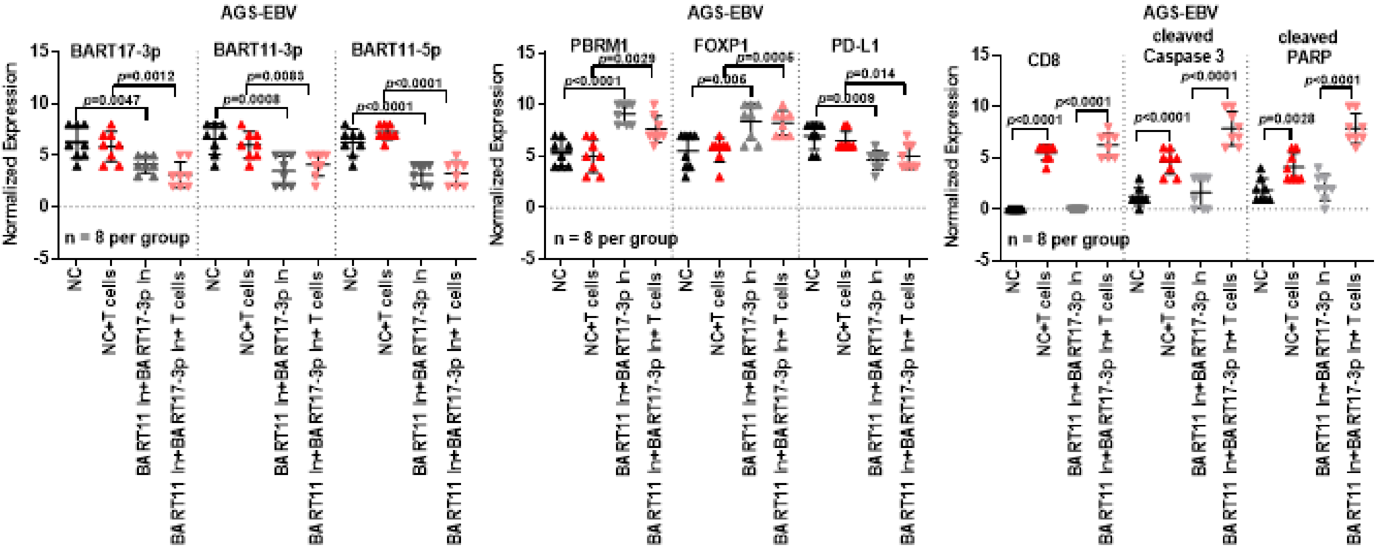
AGS-EBV
BART17-3p
BART11-3p
BART11-5p
PBRM1
FOXP1
PD-L1
CD8
cleaved Caspase 3
cleaved PARP
Normalized Expression
n = 8 per group
NC
NC+T cells
BART11 In+BART17-3p In
BART11 In+BART17-3p In+ T cells

Supplementary Fig. 22 The effects of EBV-miR-BART11 and EBV-miR-BART17-3p on tumor immune escape in xenograft mice models.

a A schematic diagram of the CDX mice models after injection of activated T cells. A density of 5×10^6 HONE1 or AGS cells were transfected with EBV-miR-BART11 and EBV-miR-BART17-3p mimics or negative control, HONE1-EBV or AGS-EBV cells were transfected with EBV-miR-BART11 and EBV-miR-BART17-3p inhibitors or negative control. Transfected cells were injected subcutaneously into the root of the right thigh of the mice. DCs and T cells were prepared and expanded simultaneously. DCs were first co-cultured with tumor cell lysate, and then co-cultured with T cells to present tumor cells specific tumor antigens to T cells, which enables them to produce tumor cells specific T cells. After 7 days, palpable tumors were formed, and 5×10^7 DiR ± T cells were infused into CDX mice through the tail vein. For the DiR-injected mice, the small animal live imaging system was used to observe the accumulation of DiR+T cells at the tumor-forming site. For one set of non-DIR-injected mice, activated T cells were injected into each tumor for another 7 days. Next, the peripheral blood of mice was extracted for flow cytometry, qRT-PCR, and ELISA. In another set, activated T cells were injected into each tumor for another 25 days and the tumor weight and volume were measured each day. The PD-L1 inhibitor (5 mg/kg, Atezolizumab) was injected to block the PD-L1/PD-1 immune checkpoint in mice and identify whether EBV miRNAs can induce tumor growth under PD-L1 inhibition after injection of HONE1 or AGS cells transfected with EBV-miR-BART11 and EBV-miR-BART17-3p mimics. Figure was created by PowerPoint (Microsoft Office 2016) and the materials of this figure were provided by the Servier Medical Art (https://smart.servier.com/) under the CC BY 3.0 license.

b The original flow cytometry results show the degree of T-cell apoptosis in each group after the injection of activated T cells for 14 days. n = 5 per group.

c The body weight of the mice was measured in each group after 25 days of activated T cells administration. n = 8 per group.

d The tumor weights for each group were measured after 25 days of activated T cells injection. n = 8 per group.

e Tumor volumes for each group after 25 days of injection with activated T cells. n = 8 per group.

f Expression of EBV-miR-BART17-3p, EBV-miR-BART11-3p, and EBV-miR-BART11-5p analyzed ascertained via ISH and the expression of PBRM1, FOXP1, PD-L1, CD8, cleaved-Caspase 3, and cleaved-PARP proteins determined via IHC were examined in the CDX nude mice sections. Magnification: 400×; scale bars = 20 μm.

g Statistical results for the expression of EBV-miR-BART17-3p, EBV-miR-BART11-3p, and EBV-miR-BART11-5p by ISH, and that of PBRM1, FOXP1, PD-L1, CD8, cleaved Caspase-3, and cleaved-PARP in the tissue sections were analyzed by IHC.

Data are presented as mean ± s.d, and *p* values are calculated by unpaired two-sided *t*-test in c-e, g. Source data are provided as a Source Data file.

Supplementary Fig. 23

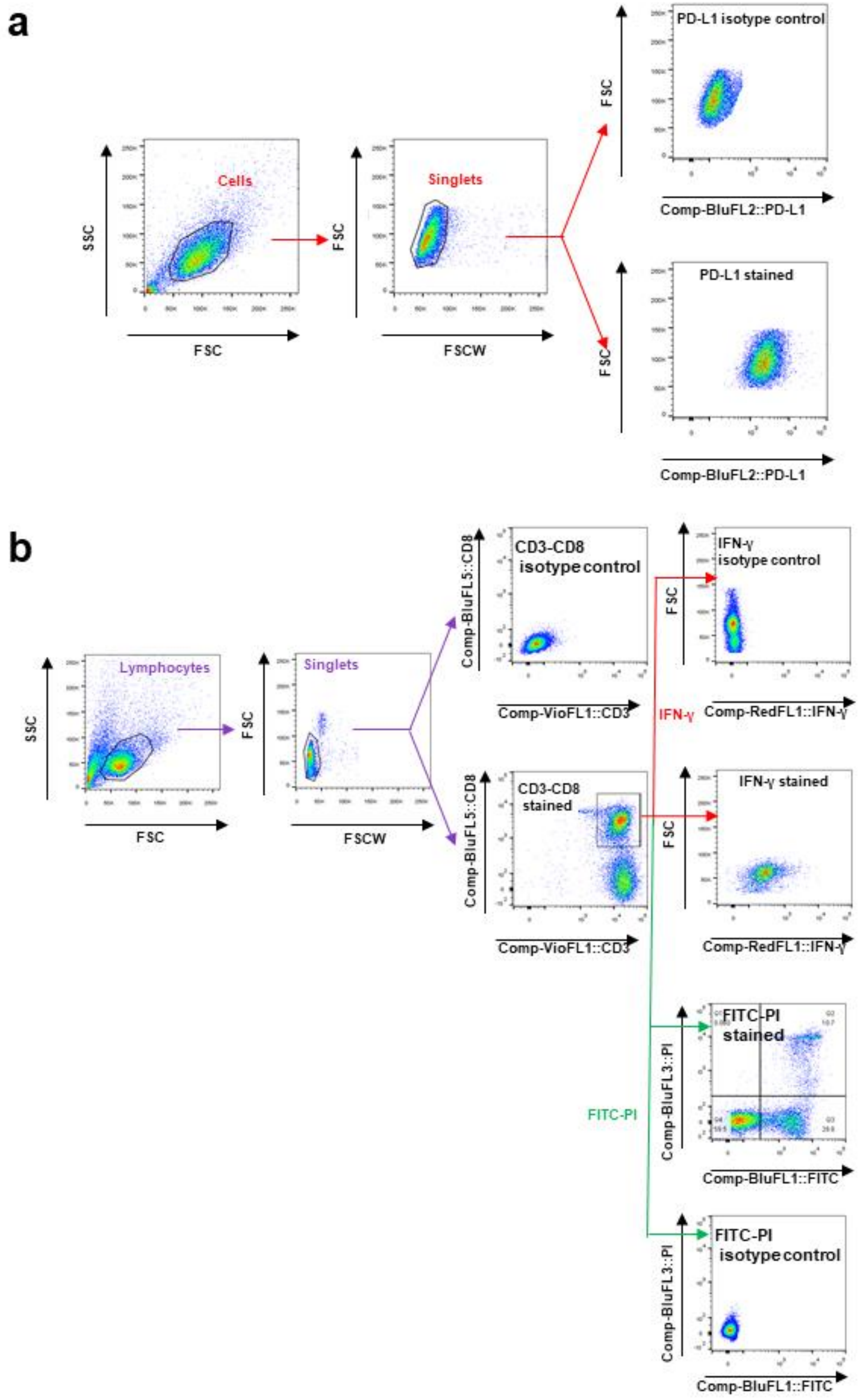

Supplementary Fig. 23 The gating strategy for the detection of PD-L1 expression in tumor cells and the analysis of CD8^{+} T cells.

a FACS gating strategy shown with a representative example for the detection of PD-L1 expression in HONE1 or HONE1-EBV cells after transfected.

b Depicted is the gating path in Co-cultured cells or mouse peripheral blood cells. Depending on the respective experimental part, FITC-PI assay of cell apoptosis was analyzed in the human CD8+ T cells and IFN-γ as a marker for T cell killing function was only assessed in Co-cultured CD8+ T cells with the addition of Brefeldin A.

Supplementary Table 1. Clinicopathological data on 52 paraffin-embedded nasopharyngeal carcinoma (NPC) and 36 nasopharyngeal adjacent epithelium (NPE) biopsies and the expression of PD-L1, FOXP1, PBRM1, EBER, BART11-3p, BART11-5p, and BART17-3p in these samples measured by immunohistochemistry (IHC) and *in situ* hybridization(ISH).

Patient No.	Gender (M=Male F=Female)	Age at Diagnosis	**WHO histological diagnosis**	T stage	N stage	M stage	Clinic stages	IHC score of PD-L1	IHC score of FOXP1	IHC score of PBRM1	ISH score of EBER1	ISH score of BART11-3p	ISH score of BART11-5p	ISH score of BART17-3p
Pat 001			non-tumor nasopharyngeal epithelial tissues	NA	NA	NA	NA	0	12	6	0	0	0	0
Pat 002			non-tumor nasopharyngeal epithelial tissues	NA	NA	NA	NA	0	7.5	7.5	0	0	0	0
Pat 003			non-tumor nasopharyngeal epithelial tissues	NA	NA	NA	NA	0	6	6	0	0	0	0
Pat 004			non-tumor nasopharyngeal epithelial tissues	NA	NA	NA	NA	0	6	7	0	0	0	0
Pat 005			non-tumor nasopharyngeal epithelial tissues	NA	NA	NA	NA	0	6	7.5	2	0	0	0
Pat 006			non-tumor nasopharyngeal epithelial tissues	NA	NA	NA	NA	0	9	5	0	0	0	0
Pat 007			non-tumor nasopharyngeal epithelial tissues	NA	NA	NA	NA	0	4	4	0	0	0	0
Pat 008			non-tumor nasopharyngeal epithelial tissues	NA	NA	NA	NA	0	4	9	0	0	0	0
Pat 009			non-tumor nasopharyngeal epithelial tissues	NA	NA	NA	NA	0	6	1.5	0	0	0	0
Pat 010			non-tumor nasopharyngeal epithelial tissues	NA	NA	NA	NA	0	6	6	0	0	0	0
Pat 011			non-tumor nasopharyngeal epithelial tissues	NA	NA	NA	NA	0	4	7.5	0	0.5	0	0
Pat 012			non-tumor nasopharyngeal epithelial tissues	NA	NA	NA	NA	0	2	2	0	1	2	0
Pat 013			non-tumor nasopharyngeal epithelial tissues	NA	NA	NA	NA	0	3	9	1	1	0	0
Pat 014			non-tumor nasopharyngeal epithelial tissues	NA	NA	NA	NA	0	2	10.5	0	1	0	0
Pat 015			non-tumor nasopharyngeal epithelial tissues	NA	NA	NA	NA	0	2	4	2	2	1	1
Pat 016			non-tumor nasopharyngeal epithelial tissues	NA	NA	NA	NA	0	8	8	2	2	2	2
Pat 017			non-tumor nasopharyngeal epithelial tissues	NA	NA	NA	NA	0	4	9	0	2	2	2
Pat 018	M:24, F:8, NA:4	less than 40 years old:3, 40 years or older:29, NA:4	non-tumor nasopharyngeal epithelial tissues	NA	NA	NA	NA	0	4	9	0	3	2	2
Pat 019			non-tumor nasopharyngeal epithelial tissues	NA	NA	NA	NA	0	6	6	0	2	2	4
Pat 020			non-tumor nasopharyngeal epithelial tissues	NA	NA	NA	NA	0	6	6	0	1	0.5	0.5
Pat 021			non-tumor nasopharyngeal epithelial tissues	NA	NA	NA	NA	0	9	9	1	4	2	0
Pat 022			non-tumor nasopharyngeal epithelial tissues	NA	NA	NA	NA	0	6	12	0	0	0	0
Pat 023			non-tumor nasopharyngeal epithelial tissues	NA	NA	NA	NA	0	12	7.5	0	0	0	0
Pat 024			non-tumor nasopharyngeal epithelial tissues	NA	NA	NA	NA	0	7.5	5.5	0	0	0	0
Pat 025			non-tumor nasopharyngeal epithelial tissues	NA	NA	NA	NA	0	9	6	0	0	0	0
Pat 026			non-tumor nasopharyngeal epithelial tissues	NA	NA	NA	NA	0.5	6	9	2	1	1	1
Pat 027			non-tumor nasopharyngeal epithelial tissues	NA	NA	NA	NA	0.5	6	6	3.5	4	2	2
Pat 028			non-tumor nasopharyngeal epithelial tissues	NA	NA	NA	NA	0.5	3.5	6	0	0	0	0
Pat 029			non-tumor nasopharyngeal epithelial tissues	NA	NA	NA	NA	1	6	6	0	0	0	0
Pat 030			non-tumor nasopharyngeal epithelial tissues	NA	NA	NA	NA	1	4	9	0	1	2	0
Pat 031			non-tumor nasopharyngeal epithelial tissues	NA	NA	NA	NA	1	9	6	3.5	2	2	2
Pat 032			non-tumor nasopharyngeal epithelial tissues	NA	NA	NA	NA	1	6	8	2	2	2	2
Pat 033			non-tumor nasopharyngeal epithelial tissues	NA	NA	NA	NA	1.5	8	8.5	2	2	2	2
Pat 034			non-tumor nasopharyngeal epithelial tissues	NA	NA	NA	NA	2	6	7.5	2	2	2	2
Pat 035			non-tumor nasopharyngeal epithelial tissues	NA	NA	NA	NA	2	9	7.5	0	0	0	0
Pat 036			non-tumor nasopharyngeal epithelial tissues	NA	NA	NA	NA	2	7.5	9	1	2	2	1
Pat 037			nasopharyngeal carcinoma (WHO II)	1	0	0	I	1	6	6	0	0	0	0
Pat 038			nasopharyngeal carcinoma (WHO II)	4	2	0	IV	1	1	5.5	0	2	1.5	2
Pat 039			nasopharyngeal carcinoma (WHO III)	3	0	0	III	2	5.5	7.5	0	2	1.5	4
Pat 040			nasopharyngeal carcinoma (WHO II)	NA	NA	NA	NA	2	2.5	6	2	2	2	1
Pat 041			nasopharyngeal carcinoma (WHO II)	4	2	0	IV	2	6	1.5	4	5	5	2
Pat 042			nasopharyngeal carcinoma (WHO II)	1	1	0	II	2	1.5	3	6	4	4	5
Pat 043			nasopharyngeal carcinoma (WHO II)	NA	NA	NA	NA	2.5	6.5	4	6	5	5	2
Pat 044			nasopharyngeal carcinoma (WHO II)	2	0	0	II	3	6.5	5.5	0	5	5	4
Pat 045			nasopharyngeal carcinoma (WHO II)	3	3	0	IV	3	6	2	0	1	1	2
Pat 046			nasopharyngeal carcinoma (WHO II)	NA	NA	NA	NA	3	8.5	12	5	4	4	2.5
Pat 047			nasopharyngeal carcinoma (WHO III)	NA	NA	NA	NA	3	4	1	6	6	6	5
Pat 048			nasopharyngeal carcinoma (WHO II)	1	1	0	II	3	3.5	4	6.5	4	4	2
Pat 049			nasopharyngeal carcinoma (WHO II)	2	2	0	III	3.5	0.5	2	0	1	1	1
Pat 050			nasopharyngeal carcinoma (WHO II)	3	2	0	III	3.5	9.5	6	0	2	2	1
Pat 051			nasopharyngeal carcinoma (WHO II)	NA	NA	NA	NA	3.5	3.5	5.5	5	2	1.5	1.5
Pat 052			nasopharyngeal carcinoma (WHO II)	2	2	0	III	3.5	7.5	8.5	5	2	2	4
Pat 053			nasopharyngeal carcinoma (WHO II)	3	2	0	III	3.5	5.5	6	9	5	5	4
Pat 054			nasopharyngeal carcinoma (WHO II)	2	2	0	III	4	0	4	0	2	2	2
Pat 055			nasopharyngeal carcinoma (WHO II)	1	1	0	III	4	2.5	6	0	4	2	4
Pat 056			nasopharyngeal carcinoma (WHO II)	1	0	0	I	4	1	4	0	3	3	2
Pat 057			nasopharyngeal carcinoma (WHO II)	1	1	0	II	4	4	6	0	5	3.5	4
Pat 058			nasopharyngeal carcinoma (WHO II)	4	0	0	IV	4	6.5	5.5	4	6	6	3.5
Pat 059			nasopharyngeal carcinoma (WHO II)	1	2	0	III	4	3	4	6	5	4	6
Pat 060			nasopharyngeal carcinoma (WHO II)	2	2	0	III	5	3.5	2.5	4	4	4	4
Pat 061			nasopharyngeal carcinoma (WHO II)	NA	NA	NA	NA	5	9	8	5	4	3.5	4
Pat 062	M:36, F:16	less than 40 years old:6, 40 years or older:46	nasopharyngeal carcinoma (WHO II)	4	0	0	IV	6	0.5	1	0.5	4	4	2
Pat 063			nasopharyngeal carcinoma (WHO II)	3	2	1	IV	6	3.5	2	4	4	4	4
Pat 064			nasopharyngeal carcinoma (WHO II)	3	0	0	III	6	0.5	3	6	9	9	6
Pat 065			nasopharyngeal carcinoma (WHO II)	4	2	0	IV	6	1	4	6	7	7	7
Pat 066			nasopharyngeal carcinoma (WHO II)	NA	NA	NA	NA	6	2.5	2	7	5	6	6
Pat 067			nasopharyngeal carcinoma (WHO II)	4	1	0	IV	6	0.5	1	9	7.5	6	6
Pat 068			nasopharyngeal carcinoma (WHO II)	2	2	0	III	6	1	3	9	6	6	5
Pat 069			nasopharyngeal carcinoma (WHO II)	NA	NA	NA	NA	6	2.5	4	9	7.5	7.5	6
Pat 070			nasopharyngeal carcinoma (WHO II)	3	1	0	III	6	2	4	9	9	6	6
Pat 071			nasopharyngeal carcinoma (WHO II)	4	2	0	IV	6	4	2.5	9	6	7.5	6
Pat 072			nasopharyngeal carcinoma (WHO II)	4	2	0	IV	7	0	4	9	7.5	7.5	7
Pat 073			nasopharyngeal carcinoma (WHO II)	4	0	0	IV	7.5	3.5	2.5	6	4	6	9
Pat 074			nasopharyngeal carcinoma (WHO II)	4	1	0	IV	7.5	2	4	9	10	9	12
Pat 075			nasopharyngeal carcinoma (WHO II)	4	2	0	IV	7.5	0	1	9	7	7	7
Pat 076			nasopharyngeal carcinoma (WHO II)	4	2	0	IV	7.5	4	3	12	10.5	7.5	6
Pat 077			nasopharyngeal carcinoma (WHO II)	NA	NA	1	IV	8	2	2	7	4	4	4
Pat 078			nasopharyngeal carcinoma (WHO II)	2	0	0	II	8	0.5	1	8.6	8	8	6.5
Pat 079			nasopharyngeal carcinoma (WHO II)	3	2	0	III	8	1.5	2	9	9	9	9
Pat 080			nasopharyngeal carcinoma (WHO II)	2	0	0	II	8	1	1.5	10.5	8.5	7.5	8
Pat 081			nasopharyngeal carcinoma (WHO II)	3	2	0	III	9	7.5	8	0	0	0	0
Pat 082			nasopharyngeal carcinoma (WHO II)	4	2	0	IV	9	5	4	6	6	6	6
Pat 083			nasopharyngeal carcinoma (WHO II)	3	0	0	III	9	3.5	1	6	6	6	6
Pat 084			nasopharyngeal carcinoma (WHO II)	1	2	0	III	9	0	5	9	9	6	6
Pat 085			nasopharyngeal carcinoma (WHO II)	3	2	0	III	9	2.5	3.5	10.5	10.5	10.5	10.5
Pat 086			nasopharyngeal carcinoma (WHO II)	2	2	0	II	10	3.5	2	10.5	6	6	6
Pat 087			nasopharyngeal carcinoma (WHO II)	3	1	0	III	12	1	4	10.5	8	9	6
Pat 088			nasopharyngeal carcinoma (WHO II)	NA	NA	NA	NA	12	0	4	12	6	8	8

WHO II = Non keratinized poorly differentiated squamous cell carcinoma
WHO III = Nonkeratinizing undifferentiated carcinoma

Supplementary Table 2. Clinicopathological data on 40 paraffin-embedded gastric adenocarcinoma (GC) and 20 normal normal gastric mucosa biopsies and the expression of PD-L1, FOXP1, PBRM1, EBER, BART11-3p, BART11-5p, and BART17-3p in these samples measured by immunohistochemistry (IHC) and *in situ* hybridization(ISH) .

Patient No.	Gender (M=Male F=Female)	Age at Diagnosis	**WHO histological diagnosis**	T stage	N stage	M stage	Clinic stages	IHC score of PD-L1	IHC score of FOXP1	IHC score of PBRM1	ISH score of EBER1	ISH score of BART11-3p	ISH score of BART11-5p	ISH score of BART17-3p
Pat 001			normal gastric mucosa tissues	NA	NA	NA	NA	0	10	12	0	0	0	0
Pat 002			normal gastric mucosa tissues	NA	NA	NA	NA	0	9	10	0	0	0	0
Pat 003			normal gastric mucosa tissues	NA	NA	NA	NA	0	11	9	0	0	0	0
Pat 004			normal gastric mucosa tissues	NA	NA	NA	NA	0	8	10	0	0	0	0
Pat 005			normal gastric mucosa tissues	NA	NA	NA	NA	1	9	11	2	1	1	3
Pat 006			normal gastric mucosa tissues	NA	NA	NA	NA	0	10	10	0	0	0	0
Pat 007			normal gastric mucosa tissues	NA	NA	NA	NA	0	9	8	0	0	0	0
Pat 008			normal gastric mucosa tissues	NA	NA	NA	NA	0	10	12	0	0	0	0
Pat 009			normal gastric mucosa tissues	NA	NA	NA	NA	0	10	9	0	0	0	0
Pat 010	M:16, F:4	less than 40 years old:1, 40 years or older:19	normal gastric mucosa tissues	NA	NA	NA	NA	0	12	10	0	0	0	0
Pat 011			normal gastric mucosa tissues	NA	NA	NA	NA	1	9	10	0	0	0	0
Pat 012			normal gastric mucosa tissues	NA	NA	NA	NA	0	9	9	0	0	0	0
Pat 013			normal gastric mucosa tissues	NA	NA	NA	NA	0	8	10	1	1	0	1
Pat 014			normal gastric mucosa tissues	NA	NA	NA	NA	0	10	12	0	0	0	0
Pat 015			normal gastric mucosa tissues	NA	NA	NA	NA	1	8	10	0	0	0	0
Pat 016			normal gastric mucosa tissues	NA	NA	NA	NA	2	8	8	2	1	0	3
Pat 017			normal gastric mucosa tissues	NA	NA	NA	NA	0	10	9	0	0	0	0
Pat 018			normal gastric mucosa tissues	NA	NA	NA	NA	0	11	12	0	0	0	0
Pat 019			normal gastric mucosa tissues	NA	NA	NA	NA	0	10	10	0	0	0	0
Pat 020			normal gastric mucosa tissues	NA	NA	NA	NA	0	10	9	0	0	0	0
Pat 021			gastric adenocarcinoma	4a	3b	0	Ⅲc	0	10	10	0	0	0	0
Pat 022			gastric adenocarcinoma	1b	0	0	Ⅰa	1	10	9	0	0	0	0
Pat 023			gastric adenocarcinoma	2	0	0	Ⅰb	2	11	7	0	0	0	0
Pat 024			gastric adenocarcinoma	1b	1	0	Ⅰb	5	8	9	0	0	0	0
Pat 025			gastric adenocarcinoma	1b	0	0	Ⅰa	1	10	9	0	0	0	0
Pat 026			gastric adenocarcinoma	2	0	0	Ⅰb	8	6	6	0	0	0	0
Pat 027			gastric adenocarcinoma	1a	0	0	Ⅰa	2	5	4	0	0	0	0
Pat 028			gastric adenocarcinoma	1a	0	0	Ⅰa	2	7	12	0	0	0	0
Pat 029			gastric adenocarcinoma	2	0	0	Ⅰb	2	8	9	0	0	0	0
Pat 030			gastric adenocarcinoma	3	3b	0	Ⅲc	0	9	12	0	0	0	0
Pat 031			gastric adenocarcinoma	3	1	0	Ⅱb	3	9	8	0	0	0	0
Pat 032			gastric adenocarcinoma	1a	1	0	Ⅰb	3	8	8	0	0	0	0
Pat 033			gastric adenocarcinoma	4a	3a	0	Ⅲb	1	9	10	0	0	0	0
Pat 034			gastric adenocarcinoma	1b	0	0	Ⅰa	1	11	10	0	0	0	0
Pat 035			gastric adenocarcinoma	3	3a	0	Ⅲb	6	9	8	0	0	0	0
Pat 036			gastric adenocarcinoma	2	0	0	Ⅰb	1	10	10	1	7	8	7
Pat 037			gastric adenocarcinoma	2	1	0	Ⅱa	4	8	9	3	3	5	8
Pat 038			gastric adenocarcinoma	2	0	0	Ⅰb	1	12	8	3	5	6	5
Pat 039			gastric adenocarcinoma	2	2	0	Ⅱb	6	5	4	3	2	7	3
Pat 040	M:31, F:9	less than 40 years old:3, 40 years or older:37	gastric adenocarcinoma	3	2	0	Ⅲa	9	7	8	3	3	4	4
Pat 041			gastric adenocarcinoma	1b	0	0	Ⅰa	9	3	4	3	2	3	3
Pat 042			gastric adenocarcinoma	1a	0	0	Ⅰa	8	6	5	3	5	4	4
Pat 043			gastric adenocarcinoma	1a	0	0	Ⅰa	5	8	6	3	4	3	4
Pat 044			gastric adenocarcinoma	1b	0	0	Ⅰa	6	7	6	4	5	3	6
Pat 045			gastric adenocarcinoma	1a	0	0	Ⅰa	7	8	4	4	3	3	2
Pat 046			gastric adenocarcinoma	2	0	0	Ⅰb	8	4	3	4	9	8	8
Pat 047			gastric adenocarcinoma	4a	1	0	Ⅲa	7	5	2	4	2	3	1
Pat 048			gastric adenocarcinoma	3	1	0	Ⅱb	2	10	6	4	6	3	4
Pat 049			gastric adenocarcinoma	1b	0	0	Ⅰa	8	8	5	5	6	4	5
Pat 050			gastric adenocarcinoma	3	0	0	Ⅱb	7	6	8	5	4	4	7
Pat 051			gastric adenocarcinoma	1b	0	0	Ⅰa	5	4	6	5	4	4	5
Pat 052			gastric adenocarcinoma	3	1	0	Ⅱb	9	3	2	9	7	8	10
Pat 053			gastric adenocarcinoma	2	0	0	Ⅰb	9	3	3	9	8	6	10
Pat 054			gastric adenocarcinoma	4a	3b	0	Ⅲc	10	1	4	10	8	7	10
Pat 055			gastric adenocarcinoma	2	0	0	Ⅰb	9	4	6	11	9	10	8
Pat 056			gastric adenocarcinoma	3	1	0	Ⅱb	10	3	2	11	10	8	8
Pat 057			gastric adenocarcinoma	2	3b	0	Ⅲb	10	2	3	12	12	10	10
Pat 058			gastric adenocarcinoma	4a	3a	0	Ⅲb	10	2	4	12	10	9	11
Pat 059			gastric adenocarcinoma	3	0	0	Ⅱa	10	8	3	12	10	11	10
Pat 060			gastric adenocarcinoma	3	1	0	Ⅱb	10	4	3	12	7	6	6

Supplementary Table 3. List of qRT-PCR primers, siRNAs and probes sequences for ISH and EMSA.

siRNA	sense	antisense
siNC	5'-UUCUUCGAACGUGUCACGUTT-3'	5'-ACGUGACACGUUCGGAGAATT-3'
siFOXP1-1	5'-CCACAGAGCUUACCUCAUA-3'	5'-UAUGAGGUAAGCUCUGUGG-3'
siFOXP1-2	5'-GCAUUGGAUGGACUUGUUU-3'	5'-AAACAAGUCCAUCCAAUGC-3'
siPBRM1-1	5'-AGUUAGGAGUUGUCGGAAUAA-3'	5'-UUAUUCCGACAACUCCUAACU-3'
siPBRM1-2	5'-GCAUCUGUCUGCAGCUAAU-3'	5'-AUUAGCUGCAGACAGAUGC-3'
siDPF2-1	5'-GUGCCACAAUUACAAUGCU-3'	5'-AGCAUUGUAAUUGUGGCAC-3'
siDPF2-2	5'-GCUCUCCAUUAAGUGCAUU-3'	5'-AAUGCACUUAAUGGAGAGC-3'

Primers	
PD-L1 -Forward	5'-CAATTTGTGCATGGAGAGGAAG-3'
PD-L1 -Reverse	5'-GTTGTATGGGGCATTGACTTTC-3'
EBER1-Forward	5'-AGGACCTACGCTGCCCTA-3'
EBER1-Reverse	5'-AAAACATGCGGACCACCA-3'
FOXP1-Forward	5'-GCAATACGGGAAGAGCTGAA-3'
FOXP1-Reverse	5'-CAAAATCTGGACTGTGGTTGG-3'
PBRM1-Forward	5'-TAACAAACCACCCCTTAC-3'
PBRM1-Reverse	5'-GCTCGTTCCAATACTTCA-3'
STK40-Forward	5'-TGGAAATAATGCAAAGAG-3'
STK40-Reverse	5'-AGGAGAGACAGCAGTGAG-3'
DPF2-Forward	5'-GGCTGTGGTGGAGAATGT-3'
DPF2-Reverse	5'-AGATGGGAAGGAAAGTCG-3'
IFN-γ-Forward	5'-TCAGCTCTGCATCGTTTTGG-3'
IFN-γ-Reverse	5'-GTTCCATTATCCGCTACATCTGAA-3'
IL-2-Forward	5'-AACTCACCAGGATGCTCACATTTA-3'
IL-2-Reverse	5'-TCCCTGGGTCTTAAGTGAAAGTTT-3'
GZMB-Forward	5'-CTGCTCACTGTTGGGGAA-3'
GZMB-Reverse	5'-TGGGGGATGGGTCTTTTC-3'
U6-Forward	5'-CTCGCTTCGGCAGCACA-3'
U6-Reverse	5'-AACGCTTCACGAATTTGCGT-3'
GAPDH-Forward	5'-GCATTGCCCTCAACGACCAC-3'
GAPDH-Reverse	5'-CCACCACCCTGTTGCTGTAG-3'

Primers for RIP and RNA pull down	
FOXP1 3'UTR(BART11-3p)-Forward	5'-TGACATGTGGGAGGGAGA-3'
FOXP1 3'UTR(BART11-3p)-Reverse	5'-CAGGAAGGCAGTGGTAGG-3'
FOXP1 3'UTR(BART11-5p)-Forward	5'-TTGGGTGCTCTGCGATAA-3'
FOXP1 3'UTR(BART11-5p)-Reverse	5'-AGCCCTCGTTAACTCCCT-3'
PBRM1 3'UTR(BART17-3p)-Forward	5'-TCAAAATGTAAGTTCCTG-3'
PBRM1 3'UTR(BART17-3p)-Reverse	5'-CAAAATCATCAACACCAA-3'

Primers for 3C	
3C-Anchor,A,B,C,D,E,F-Forward	5'-AGTAAGTCTCTTCCTGCG-3'
3C-Anchor-Reverse	5'-TCCTCCATTCCTCTTTTA-3'
3C-A-Reverse	5'-TTTCTATTTCTCCCTCCA-3',
3C-B-Reverse	5'-CCAATGTGCTGTTTCTCA-3'
3C-C-Reverse	5'-TCTGGGGGATTTTTGTTC-3'
3C-D-Reverse	5'-AATAATACCACACATCCA-3'
3C-E-Reverse	5'-GAATGACCCTTCACAATC-3'
3C-F-Reverse	5'-TGACAAGAACAAGAAATG-3'

Primers for ChIP	
ChIP-A-Forward	5'-GGTTAACAACTGATGCAA-3'
ChIP-A-Reverse	5'-AGTCCTCACAATCCAATA-3'
ChIP-B-Forward	5'-AATGAATGGCTGAAGGGT-3'
ChIP-B-Reverse	5'-CTGATGGGAATTGAGGGT-3'
ChIP-C-Forward	5'-CGCTATTCCACATCTACA-3'
ChIP-C-Reverse	5'-GTCTGTTTATGTCACTGGTT-3'
ChIP-D-Forward	5'-CACTGCTCTAAGAGTTGC-3'
ChIP-D-Reverse	5'-CAGGCCTTTGGTGAAATT-3'
ChIP-E-Forward	5'-ACTGACCTAACACGAAAA-3'
ChIP-E-Reverse	5'-GGTTCTCACAATGACTTT-3'

Primers for the luciferese activities assay of PBRM1 3'UTR	
PBRM1 WT-Forward	5'-ACTAGTAACGGGGGGACTGAGGGGAACCTGTAGGTTTAAAACAGTATGTTTGTC-3'
PBRM1 WT-Reverse	5'-AAGCCTGACAAACATACTGTTTTAAACCTACAGGTTCCCCTCAGTCCCCCCGTT-3'
PBRM1 MT-Forward	5'-ACTAGTAACGGGGGGACCTGTAGGTTTAAAACAGTATGTTTGTC-3'
PBRM1 MT-Reverse	5'-AAGCCTGACAAACATACTGTTTTAAACCTACAGGTCCCCCCGTT-3'

Primers for the luciferese activities assay of PD-L1 enhancer	
PD-L1 TRS1(-313bp-+87bp)-Forward	5’-GGGGTACCCCCCCATTCACTAACCCAAA-3’
PD-L1 TRS1(-313bp-+87bp)-Reverse	5’-CCGCTCGAGCGGACAACGCTCCCTACCTGC-3’
PD-L1 TRS2(-1,567bp-+87bp)-Forward	5’-GGGGTACCCCCCCATTCACTAACCCAAA-3’
PD-L1 TRS2(-1,567bp-+87bp)-Reverse	5’-CCGCTCGAGCGGACAACGCTCCCTACCTGC-3’
PD-L1 TRS3(-1,940bp-+87bp)-Forward	5’-GGGGTACCCCCCTGTTTTTCAATCTCCG-3’
PD-L1 TRS3(-1,940bp-+87bp)--Reverse	5’-CCGCTCGAGCGGACAACGCTCCCTACCTGC-3’
PD-L1 TRS4(-1,940bp--1,567bp)-Forward	5’-CATGATGAACTAGCAGATCATAAAGGTTCAGATGTTGGCTTGTTGTAAATTTCTTTTTT-3’
PD-L1 TRS4(-1,940bp--1,567bp)-Reverse	5’-ATTTACAACAAGCCAACATCTGAACCTTTATGATCTGCTAGTTCATCATGACTCTTGAG-3’
B-Forward	5’-CATGATGAACTAGCAGATCATAAAGGTTCAGATGTTGGCTTGTTGTAAATTTCTTTTTT-3’
B-Reverse	5’-ATTTACAACAAGCCAACATCTGAACCTTTATGATCTGCTAGTTCATCATGACTCTTGAG-3’
B-MT-Forward	5’-GGTAGACCCTGAACACTGCTTTCATCCCCACCCCCACCCCTACCCATCCCCAGTTTAAA-3’
B-MT-Reverse	5’-TGCCCTCTGATATTTTCATTTAATGGGGGCGGGGGAGCCCAATGGTCAAGACCCAGGGC-3’
E-Forward	5’-CGCGGATCCGCGATTCCCTAATGCTAATCCAC-3’
E-Reverse	5’-ACGCGTCGACGTCGGCCATAGCGGCCGCGGAATATCCCAAAGCCTTCTCGTC-3’
E-MT-Forward	5’-CATATTGGGGAACCACTGACCTAACCATCCCCACGTCGTCAGCTGTCTCAACTGTAT-3′
E-MT-Reverse	5’-TTTCAATACAGTTGAGACAGCTGACGACGTGGGGATGGTTAGGTCAGTGGTTCCCCA-3′

The probes for ISH and FISH	
EBER1	5’-AGACACCGTCCTCACCACCCGGGACTTGTA-3’
BART11-3p	5’-GGCAGTCAGCCTGGTGTGCGT-3’
BART11-5p	5’-CAACTAGCGCACCAAACTGTCTGA-3’
BART17-3p	5’-ACTAAGGGGACACCAGGCATACA-3’

The probes for EMSA	
B-WT	5’-GACCCTGAACACTGCTTTCATAAAACAAAAACAAAATACCCATCCCCAGT-3’
B-MT	5’-GACCCTGAACACTGCTTTCATCCCCACCCCCACCCCTACCCATCCCCAGT-3’
E-WT	5’-GAACCACTGACCTAACACGAAAACATGAGTCAGCTGTCTCAACTGTATTG-3’
E-MT	5’-GAACCACTGACCTAACCATCCCCACGTCGTCAGCTGTCTCAACTGTATTG-3’
non-specific probe	5’-TGTTTCAGGTTCCCCAGGGGATTCTAACCTGCAGCCATATTGGGGAACCA-3’

Supplementary Table 4. List of primary antibodies for immunohistochemistry, western blotting, immunofluorescence, flow cytometry, immunoprecipitation and ChIP.

Antibody	Catalog Number	Clone Number	Application	Dilution/Working concentration	Company
PD-L1 (E1L3N®) XP® Rabbit Monoconal antibody	13684S	E1L3N	Western Blotting	1:1000	Cell Signaling Technology
PD-L1/CD274 Mouse Monoconal Antibody	66248-1-lg	2B11D11	Western Blotting	1:1000	Proteintech Group, Inc
			IF	1:100	
PD-L1 Rabbit Monoconal antibody	ab213524	EPR19759	IHC	1:200	abcam
			IF	1:200	
PD-L1 Rabbit Monoconal antibody	RMA-0732	MXR003	IHC	1:1	MXB biotechnologies
Atezolizumab/MPDL3280A	M6101	1380723-44-3	Blocking	Cell line:5ug/ml, Mice:5mg/kg	AbMole
PE Mouse Anti-Human CD274 Monoconal antibody	557924	MIH1	FACS	1:10	BD Pharmingen
FoxP1（D35D10）XP® Rabbit Monoconal antibody	4402S	D35D10	Western Blotting	1:1000	Cell Signaling Technology
			IHC	1:100	
			IP	1:100	
			ChIP	1:100	
FOXP1 Mouse Monoconal antibody	sc-398811	A-2	IF	1:50	Santa cruz
			Western Blotting	1:500	
			IP	1:50	
BAF180/ PBRM1 Rabbit Polyclonal antibody	382286		Western Blotting	1:2000	ZENBIO
			IHC	1:200	
			IF	1:200	
PBRM1/BAF180 (E9X2Z) Rabbit Monoconal antibody	89123	E9X2Z	IP	1:50	Cell Signaling Technology
			ChIP	1:50	
AGO2 Rabbit Polyclonal antibody	10686-1-AP		RIP	1:100	Proteintech Group, Inc
			Western Blotting	1:1000	
Histone H3K27Ac Mouse Monoclonal Antibody	39085	MABI 0309	ChIP	1:100	ACTIVE MOTIF
	61634		ChIP	1:100	ACTIVE MOTIF
BAF57/ SMARCE1 Rabbit Polyclonal antibody	383214		IP	1:50	ZENBIO
			Western Blotting	1:1000	
Beta Actin Mouse Monoconal antibody	66009-1-lg	2D4H5	IP	1:200	Proteintech Group, Inc
			Western Blotting	1:5000	
SMARCA4/BRG1 Rabbit Polyclonal antibody	21634-1-AP		IP	1:100	Proteintech Group, Inc
			Western Blotting	1:1000	
DPF2 Rabbit Polyclonal antibody	12111-1-AP		IP	1:100	Proteintech Group, Inc
			Western Blotting	1:1000	
DPF2 Rabbit Polyclonal antibody	ab128149		ChIP	1:50	abcam
BV421 Mouse Anti-Human CD3 Monoconal antibody	563798	SK7	FACS	1:10	BD Pharmingen
APC Mouse Anti-Human IFN-γ Monoconal antibody	554702	B27	FACS	1:10	BD Pharmingen
PE-Cy^{TM}7 Mouse Anti-Human CD8 Monoconal antibody	557750	RPA-T8	FACS	1:10	BD Pharmingen
CD8 Rabbit Monoconal antibody	RMA-0514	SP16	IHC	1:1	MXB biotechnologies
Cleaved PARP(Asp214)(D64E10) XP® Rabbit Monoconal antibody	5625T	D64E10	IHC	1:50	Cell Signaling Technology
Cleaved Capase-3(Asp175)(5A1E) XP® Rabbit Monoconal antibody	9664T	5A1E	IHC	1:200	Cell Signaling Technology
GAPDH Rabbit Polyclonal antibody	10494-1-AP		Western Blotting	1:5000	Proteintech Group, Inc
Normal Mouse IgG Polyclonal Antibody	12-371		IP	1:200	Millipore
Normal Rabbit IgG Polyclonal Antibody	12-370		IP	1:200	Millipore
