# Supplementary material for: EBV miRNAs BART11 and BART17-3p promote immune escape through the enhancer-mediated transcription of PD-L1
Source: Nat Commun. 2022 Feb 14;13:866. doi: 10.1038/s41467-022-28479-2 (PMC8844414; doi:10.1038/s41467-022-28479-2)
Supplement: Supplementary file 3 — Description of Additional Supplementary Files [file 41467_2022_28479_MOESM3_ESM.pdf]

Description of Additional Supplementary Files

Supplementary Data 1: The Mass spectrometry identification of proteins pulled down by FOXP1.

Supplementary Data 2: Clinicopathological data of 82 NPC tissues and 31 non-neoplastic nasopharyngeal epithelial tissue samples for qRT-PCR.

Supplementary Movie 1: The activity status of primary T cells co-cultured with HONE1 transfected with negative control.

Supplementary Movie 2: The activity status of primary T cells co-cultured with HONE1 overexpressing EBV-miR-BART11 and EBV-miR-BART17-3p.

Supplementary Movie 3: The activity status of primary T cells co-cultured with AGS transfected with negative control.

Supplementary Movie 4: The activity status of primary T cells co-cultured with AGS overexpressing EBV-miR-BART11 and EBV-miR-BART17-3p.
