# Supplementary material for: EBV miRNAs BART11 and BART17-3p promote immune escape through the enhancer-mediated transcription of PD-L1
Source: Nat Commun. 2022 Feb 14;13:866. doi: 10.1038/s41467-022-28479-2 (PMC8844414; doi:10.1038/s41467-022-28479-2)
Supplement: Supplementary file 10 — Reporting Summary [file 41467_2022_28479_MOESM10_ESM.pdf]

nature research

Corresponding author(s): Zhaoyang Zeng, Wei Xiong

Last updated by author(s): Dec 1, 2021

Reporting Summary

Nature Research wishes to improve the reproducibility of the work that we publish. This form provides structure for consistency and transparency in reporting. For further information on Nature Research policies, see our Editorial Policies and the Editorial Policy Checklist.

Statistics

For all statistical analyses, confirm that the following items are present in the figure legend, table legend, main text, or Methods section.

n/a	Confirmed	
☐	☒	The exact sample size (*n*) for each experimental group/condition, given as a discrete number and unit of measurement
☐	☒	A statement on whether measurements were taken from distinct samples or whether the same sample was measured repeatedly
☐	☒	The statistical test(s) used AND whether they are one- or two-sided<br>*Only common tests should be described solely by name; describe more complex techniques in the Methods section.*
☒	☐	A description of all covariates tested
☐	☒	A description of any assumptions or corrections, such as tests of normality and adjustment for multiple comparisons
☐	☒	A full description of the statistical parameters including central tendency (e.g. means) or other basic estimates (e.g. regression coefficient) AND variation (e.g. standard deviation) or associated estimates of uncertainty (e.g. confidence intervals)
☐	☒	For null hypothesis testing, the test statistic (e.g. *F*, *t*, *r*) with confidence intervals, effect sizes, degrees of freedom and *P* value noted<br>*Give P values as exact values whenever suitable.*
☒	☐	For Bayesian analysis, information on the choice of priors and Markov chain Monte Carlo settings
☒	☐	For hierarchical and complex designs, identification of the appropriate level for tests and full reporting of outcomes
☐	☒	Estimates of effect sizes (e.g. Cohen's *d*, Pearson's *r*), indicating how they were calculated

Our web collection on statistics for biologists contains articles on many of the points above.

Software and code

Policy information about availability of computer code

Data collection	FlowJo CE software (Treestar), used for FACS<br>Volocity software, version 6.1.1 (PerkinElmer), used for IF<br>SageCaptureTM software (SAGECREATION), used for Western Blotting, EMSA<br>Quantity One software (Bio-Rad) ,used for Qrt-pcr products Gel Electrophoresis<br>CFX Manager TM software, version 3.1 (Bio-Rad), used for Qrt-pcr<br>SoftMax® Pro 7 software, version 7.1.0 (MolecularDevices), used for ELISA and Luciferase reporter assay<br>Harmony software, version 4.9 (PerkinElmer), used for the high-content screening system<br>Bruker software MI SE 700 (Bruker Corporation), used for the small animal in vivo imaging system<br>JASPAR CORE database, the 9th release (2022), used for finding FOXP1 binding sites
Data analysis	GraphPad Prism version 8 was used for data analysis.<br>FACS data was analyzed by with FlowJo version 10 (Treestar).<br>MS data was analyzed by the Proteome Discoverer 1.4 software (Thermo Fisher) and the files were imported and used to search the UniProtKB/Swiss-Prot database.<br>ChIP-Seq data visualization has been carried out using UCSC browser.

For manuscripts utilizing custom algorithms or software that are central to the research but not yet described in published literature, software must be made available to editors and reviewers. We strongly encourage code deposition in a community repository (e.g. GitHub). See the Nature Research guidelines for submitting code & software for further information.

Data

Policy information about availability of data

All manuscripts must include a data availability statement. This statement should provide the following information, where applicable:

- Accession codes, unique identifiers, or web links for publicly available datasets
- A list of figures that have associated raw data
- A description of any restrictions on data availability

Data referenced in this study are available in The Cancer Genome Atlas (https://tcga-data.nci.nih.gov/docs/publications/stad_2014/) Gene Expression Omnibus with the accession code GSE12452, GSE65801, GSE51575, GSE32960, GSE36682, GSE64634 and GSE95749 (https://www.ncbi.nlm.nih.gov/geo/) JASPAR CORE database, the 9th release (2022) (https://jaspar.genereg.net/) ENCODE database displayed in UCSC Browser (http://genome.ucsc.edu/cgi-bin/hgTracks?db=hg19&lastVirtModeType=default&lastVirtModeExtraState=&virtModeType=default&virtMode=0&nonVirtPosition=&position=chr9%3A5430000%2D5500000&hgsid=1226038355_xQJAKzHP0remahAa3CmCprv7ihQT). The mass spectrometry proteomics data generated in this study are provided in the Supplementary Table 1. Source data are provided with this paper.

Field-specific reporting

Please select the one below that is the best fit for your research. If you are not sure, read the appropriate sections before making your selection.

[x] Life sciences [ ] Behavioural & social sciences [ ] Ecological, evolutionary & environmental sciences

For a reference copy of the document with all sections, see nature.com/documents/nr-reporting-summary-flat.pdf

Life sciences study design

All studies must disclose on these points even when the disclosure is negative.

Sample size	No Sample size calculation was performed. Sample size was determined according to previous published literature. References: Ge, J.S., et al. Epstein-Barr virus-encoded circular RNA circBART2.2 promotes immune escape of nasopharyngeal carcinoma by regulating PD-L1. Cancer Research 81, 5074-5088 (2021).
Data exclusions	No data was excluded for all in vitro experiments. We did not perform any pre-established exclusions for in vivo experiments. In in vivo survival studies, we excluded small number of animals died due to other than tumor-related condition.
Replication	As reported in the figure legends, in vitro experiments were performed at least three times with similar results, the findings were reliably reproduced.
Randomization	Mice were randomly allocated to experimental groups by the animal care taker upon delivery.
Blinding	Due to the need for T cells therapy and PD-L1 inhibitor therapy on some mice during the research experiment, the number of personnel available for these studies is limited, so blinding was not performed. It is not relevant since tumor measurements were performed by person blinded to which animal was being measured and analyses were done objectively by automatic devices (FACS, plate reader etc.).

Reporting for specific materials, systems and methods

We require information from authors about some types of materials, experimental systems and methods used in many studies. Here, indicate whether each material, system or method listed is relevant to your study. If you are not sure if a list item applies to your research, read the appropriate section before selecting a response.

Materials & experimental systems

n/a	Involved in the study	
[ ]	[x]	Antibodies
[ ]	[x]	Eukaryotic cell lines
[x]	[ ]	Palaeontology and archaeology
[ ]	[x]	Animals and other organisms
[ ]	[x]	Human research participants
[x]	[ ]	Clinical data
[x]	[ ]	Dual use research of concern

Methods

n/a	Involved in the study	
[x]	[ ]	ChIP-seq
[ ]	[x]	Flow cytometry
[x]	[ ]	MRI-based neuroimaging

Antibodies

Antibodies used	PD-L1 (E1L3N®) XP® Rabbit Monoconal antibody, Cat#13684S, Clone E1L3N, Western Blotting 1:1000, Cell Signaling Technology<br>PD-L1/CD274 Mouse Monoconal Antibody, Cat#66248-1-Ig , Clone 2B11D11, Western Blotting 1:1000, IF 1:100, Proteintech Group, Inc

PD-L1 Rabbit Monoconal antibody, Cat#ab213524 , Clone EPR19759 , IHC 1:200 , IF 1:200 , abcam
PD-L1 Rabbit Monoconal antibody, Cat#RMA-0732, Clone MXR003, IHC 1:1, MXB biotechnologies
Atezolizumab/MPDL3280A, Cat#M6101, Clone 1380723-44-3, Blocking Cell line:5ug/ml, Mice:5mg/kg, AbMole
PE Mouse Anti-Human CD274 Monoconal antibody, Cat#557924 , Clone MIH1, FACS 1:10, BD Pharmingen
FoxP1(D35D10) XP® Rabbit Monoconal antibody, Cat#4402S, Clone D35D10, Western Blotting 1:1000, IHC 1:100, IP 1:100, ChIP 1:100, Cell Signaling Technology
FOXP1 Mouse Monoconal antibody, Cat#sc-398811, Clone A-2, IF 1:50, Western Blotting 1:500, IP 1:50, Santa cruz
BAF180/ PBRM1 Rabbit Polyclonal antibody, Cat#382286, Western Blotting 1:2000, IHC 1:200, IF 1:200, ZENBIO
PBRM1/BAF180 (E9X2Z) Rabbit Monoconal antibody, Cat#89123, Clone E9X2Z, IP 1:50, ChIP 1:50, Cell Signaling Technology
AGO2 Rabbit Polyclonal antibody, Cat#10686-1-AP, RIP 1:100, Western Blotting 1:1000, Proteintech Group, Inc
Histone H3K27Ac Mouse Monoclonal Antibody, Cat#39085, Clone MABI 0309, ChIP 1:100, ACTIVE MOTIF
Histone H3K4me1 Mouse Polyclonal Antibody, Cat#61634, ChIP 1:100, ACTIVE MOTIF
BAF57/ SMARCE1 Rabbit Polyclonal antibody, Cat#383214 , IP 1:50, Western Blotting 1:1000, ZENBIO
Beta Actin Mouse Monoconal antibody, Cat#66009-1-Ig, Clone 2D4H5, IP 1:200, Western Blotting 1:5000, Proteintech Group, Inc
SMARCA4/BRG1 Rabbit Polyclonal antibody, Cat#21634-1-AP, IP 1:100, Western Blotting 1:1000, Proteintech Group, Inc
DPF2 Rabbit Polyclonal antibody, Cat#12111-1-AP, IP 1:100, Western Blotting 1:1000, Proteintech Group, Inc
DPF2 Rabbit Polyclonal antibody, Cat#ab128149, ChIP 1:50, abcam
BV421 Mouse Anti-Human CD3 Monoconal antibody, Cat#563798, Clone SK7, FACS 1:10, BD Pharmingen
APC Mouse Anti-Human IFN-γ Monoconal antibody, Cat#554702, Clone B27, FACS 1:10, BD Pharmingen
PE-CyTM7 Mouse Anti-Human CD8 Monoconal antibody, Cat#557750, Clone RPA-T8, FACS 1:10, BD Pharmingen
CD8 Rabbit Monoconal antibody, Cat#RMA-0514, Clone SP16, IHC 1:1, MXB biotechnologies
Cleaved PARP(Asp214)(D64E10) XP® Rabbit Monoconal antibody, Cat#5625T Clone D64E10, IHC 1:50, Cell Signaling Technology
Cleaved Capase-3(Asp175)(5A1E) XP® Rabbit Monoconal antibody, Cat#9664T, Clone 5A1E , IHC 1:200, Cell Signaling Technology
GAPDH Rabbit Polyclonal antibody, Cat#10494-1-AP, Western Blotting 1:5000, Proteintech Group, Inc
Normal Mouse IgG Polyclonal Antibody, Cat#12-371, IP 1:200, Millipore
Normal Rabbit IgG Polyclonal Antibody, Cat#12-370 , IP 1:200, Millipore

Validation

All antibodies are commercially available and were validated by the manufacturer as follows:
PD-L1 (E1L3N®) XP® Rabbit Monoconal antibody, Cat#13684S, Cell Signaling Technology, validation from the manufacturer's website: Product was tested in Western Blotting. The manufacturer states that the antibody was used in 355 publications. (https://www.cellsignal.cn/products/primary-antibodies/pd-l1-e1l3n-xp-rabbit-mab/13684?site-search-type=Products&N=4294956287&Ntt=13684s&fromPage=plp&_requestid=1155074)
PD-L1/CD274 Mouse Monoconal Antibody, Cat#66248-1-Ig, Proteintech Group, Inc: The antibody was used in 50 publications for Western Blot and 17 publications for IF listed on the manufacturer's website. (https://www.ptglab.com/products/PD-L1-CD274-Antibody-66248-1-Ig.htm)
PD-L1 Rabbit Monoconal antibody, Cat#ab213524 , abcam: The manufacturer states that this antibody can be used for IHC and IF, and was used in 36 publications. (https://www.abcam.cn/pd-l1-antibody-epr19759-ab213524.html)
PD-L1 Rabbit Monoconal antibody, Cat#RMA-0732, MXB biotechnologies, validation from the manufacturer's website (http://www.maxim.com.cn/sitecn/xpsd/7384.html)
Atezolizumab/MPDL3280A, AbMole, validation from the manufacturer's website (http://www.abmole.cn/products/atezolizumab.html), and was used for blocking in 1 publication.
PE Mouse Anti-Human CD274 Monoconal antibody, Cat#557924 , BD Pharmingen: The manufacturer states that product routinely tested in FACS. (https://www.bdbiosciences.com/en-us/products/reagents/flow-cytometry-reagents/research-reagents/single-color-antibodies-ruo/pe-mouse-anti-human-cd274.557924)
FoxP1(D35D10) XP® Rabbit Monoconal antibody, Cat#4402S, Cell Signaling Technology: The manufacturer states that product can be used in Western Blot, IP, IHC, ChIP, The antibody was used in 10 publications listed on the manufacturer's website. (https://www.cellsignal.cn/products/primary-antibodies/foxp1-d35d10-xp-rabbit-mab/4402?site-search-type=Products&N=4294956287&Ntt=4402s&fromPage=plp&_requestid=1178781)
FOXP1 Mouse Monoconal antibody, Cat#sc-398811, Santa cruz: The manufacturer states that product can be used in Western Blot, IP, IF, The antibody was used in 2 publications. (https://datasheets.scbt.com/sc-398811.pdf)
BAF180/ PBRM1 Rabbit Polyclonal antibody, Cat#382286, ZENBIO: The manufacturer states that product can be used in Western Blot, IP, IHC. (http://www.zen-bio.cn/prod_view.aspx?TypeId=136&Id=381380&FId=t3:136:3)
PBRM1/BAF180 (E9X2Z) Rabbit Monoconal antibody, Cat#89123, Cell Signaling Technology: The manufacturer states that product was tested in IP, ChIP. (https://www.cellsignal.cn/products/primary-antibodies/pbrm1-baf180-e9x2z-rabbit-mab/89123?site-search-type=Products&N=4294956287&Ntt=89123&fromPage=plp&_requestid=1180309)
AGO2 Rabbit Polyclonal antibody, Cat#10686-1-AP, Proteintech Group, Inc: The antibody was used in 19 publications for Western Blot and 16 publications for RIP listed on the manufacturer's website. (https://www.ptglab.com/products/EIF2C2-Antibody-10686-1-AP.htm)
Histone H3K27Ac Mouse Monoclonal Antibody, Cat#39085, ACTIVE MOTIF: The manufacturer states that product was used in numerous publications for ChIP. (https://www.activemotif.com/catalog/details/39685/histone-h3-acetyl-lys27-antibody-mab-clone-mabi-0309)
Histone H3K4me1 Mouse Polyclonal Antibody, Cat#61634, ACTIVE MOTIF: The manufacturer states that product was tested in ChIP. (https://www.activemotif.com/catalog/details/61633/histone-h3k4me1-antibody-pab)
BAF57/ SMARCE1 Rabbit Polyclonal antibody, Cat#383214 , ZENBIO: The manufacturer states that product was tested in IP, Western Blot. (http://www.zen-bio.cn/prod_view.aspx?TypeId=136&Id=368612&FId=t3:136:3)
Beta Actin Mouse Monoconal antibody, Cat#66009-1-Ig, Proteintech Group, Inc: The manufacturer states that product was tested in IP, Western Blot, and was used in 2372 publications listed on the manufacturer's website. (https://www.ptgcn.com/products/Pan-Actin-Antibody-66009-1-Ig.htm)
SMARCA4/BRG1 Rabbit Polyclonal antibody, Cat#21634-1-AP, Proteintech Group, Inc: The manufacturer states that product was tested in IP, Western Blot, and was used in 18 publications listed on the manufacturer's website. (https://www.ptgcn.com/products/SMARCA4-Antibody-21634-1-AP.htm)

DPF2 Rabbit Polyclonal antibody, Cat#12111-1-AP, Proteintech Group, Inc: The manufacturer states that product was tested in IP, Western Blot, and was used in 5 publications listed on the manufacturer's website. (https://www.ptgcn.com/products/DPF2-Antibody-12111-1-AP.htm)
DPF2 Rabbit Polyclonal antibody, Cat#ab128149, abcam: Product was used for ChIP in 1 review listed on the manufacturer's website. (https://www.abcam.cn/dpf2req-antibody-ab128149.html?productWallTab=Abreviews)
BV421 Mouse Anti-Human CD3 Monoconal antibody, Cat#563798, BD Pharmingen: The manufacturer states that this antibody is quality control tested by FACS. (https://www.bdbiosciences.com/en-us/products/reagents/flow-cytometry-reagents/research-reagents/single-color-antibodies-ruo/bv421-mouse-anti-human-cd3.563798)
APC Mouse Anti-Human IFN-γ Monoconal antibody, Cat#554702, BD Pharmingen: The manufacturer states that this antibody is quality control tested by FACS. (https://www.bdbiosciences.com/en-us/products/reagents/flow-cytometry-reagents/research-reagents/single-color-antibodies-ruo/apc-mouse-anti-human-ifn.554702)
PE-CyTM7 Mouse Anti-Human CD8 Monoconal antibody, Cat#557750, BD Pharmingen: The manufacturer states that this antibody is routinely tested by FACS. (https://www.bdbiosciences.com/en-us/products/reagents/flow-cytometry-reagents/research-reagents/single-color-antibodies-ruo/pe-cy-7-mouse-anti-human-cd8.557750)
CD8 Rabbit Monoconal antibody, Cat#RMA-0514, MXB biotechnologies, validation from the manufacturer's website: http://www.maxim.com.cn/sitecn/dklkthdklkt/7013.html
Cleaved PARP(Asp214)(D64E10) XP® Rabbit Monoconal antibody, Cat#5625T,Cell Signaling Technology: The manufacturer states that product was tested in IHC, and was used in 15 publications for IHC listed on the manufacturer's website. (https://www.cellsignal.cn/products/primary-antibodies/cleaved-parp-asp214-d64e10-xp-rabbit-mab/5625?site-search-type=Products&N=4294956287&Ntt=5625t&fromPage=plp&_requestid=1186948)
Cleaved Capase-3(Asp175)(5A1E) XP® Rabbit Monoconal antibody, Cat#9664T, Cell Signaling Technology: The manufacturer states that product was tested in IHC, and was used in 329 publications for IHC listed on the manufacturer's website. (https://www.cellsignal.cn/products/primary-antibodies/cleaved-caspase-3-asp175-5a1e-rabbit-mab/9664?site-search-type=Products&N=4294956287&Ntt=9664t&fromPage=plp&_requestid=1187120)
GAPDH Rabbit Polyclonal antibody, Cat#10494-1-AP, Proteintech Group, Inc: The manufacturer states that product was tested in Western Blot, and was used in 2831 publications for Western Blot listed on the manufacturer's website. (https://www.ptgcn.com/products/GAPDH-Antibody-10494-1-AP.htm)
Normal Mouse IgG Polyclonal Antibody, Cat#12-371, Millipore: The manufacturer states that product can used for IP. (https://www.sigmaaldrich.cn/CN/zh/product/mm/12371?context=product)
Normal Rabbit IgG Polyclonal Antibody, Cat#12-370 , IP 1:200, Millipore: The manufacturer states that product can used for IP. (https://www.sigmaaldrich.cn/CN/zh/product/mm/12370?context=product)

Eukaryotic cell lines

Policy information about cell lines

Cell line source(s)	Cell lines including the human EBV-negative immortalized normal nasopharyngeal epithelial cell line NP69, the EBV-positive NPC cell line C666-1, the EBV-negative NPC cell lines HNE2, and CNE2, the EBV-positive human Burkitt lymphoma Akata cells, EBV-transformed marmoset leukocyte B95-8 cells, and the human T lymphocyte leukemia cell line Jurkat were obtained from Cancer Research Institute, Central South University. The EBV-negative NPC cell line HONE1, and the EBV-positive HONE1-EBV was a generous gift from Professor George Sai Wah Tsao, University of Hong Kong and Professor Xin Li, Southern Medical University. HONE-1-EBV cell line was constructed by team of Professor George Sai Wah Tsao, University of Hong Kong. The method of establishing HONE-1-EBV by introducing the EBV genome into the respective parental cell line HONE-1 was the same as described by Lo et al. The EBV-negative GC cell line AGS, and the EBV-positive AGS-EBV cell line was constructed and donated by Professor TAKADA, HOKKAIDO University; Professor Lunquan Sun from Xiangya Hospital of Central South University. The EBV-positive GC cell line SNU-719, normal gastric epithelial cell line GES-1 were purchased fromZhongqiaoxinzhou Biotech. References: Lo, A.K.F., et al. Epstein-Barr virus infection alters cellular signal cascades in human nasopharyngeal epithelial cells. Neoplasia 3, 173-180 (2006). YOSHIYAMA,H., et al. Epstein-Barr virus infection of human gastric carcinoma cells implication of the existence of a new virus receptor different from CD21. J virol 71, 5688-5691 (1997).
Authentication	Cells were not recently authenticated.
Mycoplasma contamination	All cell lines in our laboratory are routinely tested for mycoplasma contamination and cells used in this study are negative for mycoplasma.
Commonly misidentified lines (See ICLAC register)	No commonly misidentified cell lines used in this study.

Animals and other organisms

Policy information about studies involving animals; ARRIVE guidelines recommended for reporting animal research

Laboratory animals	Mice (BALB/c Nude, 4 weeks old, female) were obtained from the Laboratory Animal Center of Central South University and used as a host for tumor formation and adoptive T cell treatment. The tumors derived from these mice were used for gene expression analysis and T cell profiling. All mice were maintained under SPF conditions in a controlled environment of 20–22 °C, with a 12/12 h light/dark cycle, 50–70% humidity, and food and water provided ad libitum.
Wild animals	The study did not involve wild animals.
Field-collected samples	The study did not involve samples collected from field.
Ethics oversight	All procedures were approved by the Ethics Committee of Central South University.

Note that full information on the approval of the study protocol must also be provided in the manuscript.

Human research participants

Policy information about studies involving human research participants

Population characteristics	We have ensured the manuscript including supplementary tables do not contain 3 or more indentifiers of the study participants.
Recruitment	The patients and health donors were recruited by Cancer Hospital of Central South University and the Second Xiangya Hospital of Central South University, and informed consent.
Ethics oversight	The tissue samples informed consent of the patients and health donors were obtained as authorized by the Ethics Committee of Central South University before use.

Note that full information on the approval of the study protocol must also be provided in the manuscript.

Flow Cytometry

Plots

Confirm that:

- [x] The axis labels state the marker and fluorochrome used (e.g. CD4-FITC).
- [x] The axis scales are clearly visible. Include numbers along axes only for bottom left plot of group (a 'group' is an analysis of identical markers).
- [x] All plots are contour plots with outliers or pseudocolor plots.
- [x] A numerical value for number of cells or percentage (with statistics) is provided.

Methodology

Sample preparation	T cells were expanded in vitro by adding CD3/CD28 MACSiBead (Miltenyi) and 15 ng/ml IL-2, 5 ng/ml IL-7, and 10 ng/ml IL-15 (Sino Biological) to PBMCs for 8 days. To generate tumor-specific T cells, the prepared dendritic cells and the expanded T cells were co-cultured at a 1:5 ratio for 5 days in the medium supplemented with IL-2, IL-7, and IL-15. The transfected tumor cells and activated human primary T cells were co-cultured in a 24-well plate for 3 h in a 1:10 ratio. Anti-CD3 and anti-CD8 were added for 20 minutes for surface staining. The cells were then washed and resuspended in 1 ml of freshly prepared solution at 4°C. After being washed with Wash buffer, the cells were stained with anti-IFN-γ 30 min. For cell apoptosis analysis, cells were treated by Apoptosis Detection Kit (BD Pharmingen). Briefly, cells were collected and rinsed with 1x binding buffer, and then stained with Annexin V and PI in binding buffer at 4°C for 10 mins and directly run on a flow cytometer.
Instrument	All samples were acquired on the DxP AthenaTM flow cytometer (Cytek).
Software	All data were analyzed with FlowJo V10 (Treestar).
Cell population abundance	The abundance of the cell population was analyzed and monitored by Flowjo while sorting.
Gating strategy	FSC/SSC gating was based on the cellular specific surface markers. Cell viability marker was applied to ensure the accuracy of each gating.

- [x] Tick this box to confirm that a figure exemplifying the gating strategy is provided in the Supplementary Information.
